# CircPTK2 Suppresses the Progression of Gastric Cancer by Targeting the MiR-196a-3p/AATK Axis

**DOI:** 10.3389/fonc.2021.706415

**Published:** 2021-09-15

**Authors:** Ling Gao, Tingting Xia, Mingde Qin, Xiaofeng Xue, Linhua Jiang, Xinguo Zhu

**Affiliations:** ^1^Department of General Surgery, The First Affiliated Hospital of Soochow University, Suzhou, China; ^2^Department of Gastroenterology, The First Affiliated Hospital of Soochow University, Suzhou, China; ^3^Department of the Stem Cell and Biomedical Material Key Laboratory of Jiangsu Province (the State Key Laboratory Incubation Base), Soochow University, Suzhou, China

**Keywords:** gastric cancer, circPTK2, miR-196a-3p, AATK, proliferation

## Abstract

**Background:**

Gastric cancer is a type of malignant tumor with high morbidity and mortality. It has been shown that circular RNAs (circRNAs) exert critical roles in gastric cancer progression *via* working as microRNA (miRNA) sponges to regulate gene expression. However, the role and potential molecular mechanism of circRNAs in gastric cancer remain largely unknown.

**Methods:**

CircPTK2 (hsa_circ_0005273) was identified by bioinformatics analysis and validated by RT-qPCR assay. Bioinformatics prediction, dual-luciferase reporter, and RNA pull-down assays were used to determine the interaction between circPTK2, miR-196a-3p, and apoptosis-associated tyrosine kinase 1 (AATK).

**Results:**

The level of circPTK2 was markedly downregulated in gastric cancer tissues and gastric cancer cells. Upregulation of circPTK2 significantly suppressed the proliferation, migration, and invasion of gastric cancer cells, while circPTK2 knockdown exhibited opposite effects. Mechanically, circPTK2 could competitively bind to miR-196a-3p and prevent miR-196a-3p to reduce the expression of AATK. In addition, overexpression of circPTK2 inhibited tumorigenesis in a xenograft mouse model of gastric cancer.

**Conclusion:**

Collectively, circPTK2 functions as a tumor suppressor to suppress gastric cancer cell proliferation, migration, and invasion through regulating the miR-196a-3p/AATK axis, suggesting that circPTK2 may serve as a novel therapeutic target for gastric cancer.

## Introduction

Gastric cancer is the fifth most common malignancy worldwide and is the third leading cause of cancer-related deaths (1–3). Although the current clinical diagnosis and treatment for gastric cancer are continuously improving, the 5-year survival rate of patients with gastric cancer is less than 30% (4, 5). Gastric cancer is a complex cellular network (6, 7). Currently, the etiology and pathogenesis are not yet clear, which brings corresponding difficulties to its early diagnosis and treatment (6, 7).

Circular RNAs (circRNAs) are a class of non-coding RNAs derived from back-spliced exons (8, 9). Unlike linear RNA, circRNAs are covalently closed continuous loops that lack 5′ (cap) and 3′ (polyadenylation) ends (10). Meanwhile, circRNAs are found to be relatively stable and evolutionally conserved in the cytoplasm (10, 11). CircRNAs have been found to be implicated in the progression of cancers (12, 13). Significantly, circRNAs are aberrantly expressed in multiform types of cancer, including gastric cancer (14, 15). However, the biological function of circRNAs in gastric cancer remains largely unclear.

MicroRNAs (miRNAs) are a kind of non-coding RNAs with 19–25 nucleotides in length (16, 17). It has been shown that miRNA can regulate gene expression at the post-transcriptional level (18, 19). Considerable studies reported that circRNAs could exhibit their biological roles through acting as “miRNA sponges” to regulate gene expressions (20, 21). For instance, Zhang et al. reported that circNRIP1 could promote gastric cancer progression through sponging miR-149-5p (22). Luo et al. found that circCCDC9 could inhibit the proliferation of gastric cancer cells *via* targeting the miR-6792-3p/CAV1 axis (23).

In this study, we screened differentially expressed circRNAs (DEcircRNAs) between gastric cancer tissues and normal tissues and found that circPTK2 was significantly downregulated in gastric cancer tissues. In addition, circPTK2 could inhibit the proliferation, migration, and invasion of gastric cancer cells through functioning as a miRNA sponge to upregulate the expression of the tumor-suppressor gene AATK. These data indicated that circPTK2 may be used as a potential target in gastric cancer therapy.

## Materials and Methods

### Identification of Differentially Expressed CircRNAs

Gastric cancer-related datasets (GSE93541, GSE89143, and GSE78092) were downloaded from the GEO database. For the GSE93541 dataset, R language was utilized to analyze the DEcircRNAs in plasma samples from gastric cancer patients and healthy controls. For the GSE89143 and GSE78092 datasets, R language was utilized to analyze the DEcircRNAs between gastric cancer tissues and adjacent normal tissues. The threshold value of differentially expressed genes was set at two times of different multiple and *p <*0.05. The intersection of DEcircRNAs from three datasets was performed using the Venn diagram package.

### Specimen Collection

Gastric cancer tissues and matched adjacent normal tissues were obtained from the First Affiliated Hospital of Soochow University. Written consent was obtained from each patient with gastric cancer. All samples were frozen in liquid nitrogen and stored at −80°C. This study was approved by the Ethics Committee of The First Affiliated Hospital of Soochow University.

### Cell Culture

Human gastric epithelial cell line GES-1 and human AGS, MKN45, and SNU-5 cell lines were obtained from ATCC (Manassas, VA, USA). Cells were maintained in DMEM medium (Thermo Fisher Scientific, Waltham, MA, USA) containing 10% fetal bovine serum (FBS) and cultured in a 5% CO_2_ incubator at 37°C.

### Cell Transfection

MiR-196a-3p mimics and miR-196a-3p inhibitor were designed and synthesized by RiboBio (Guangzhou, China). After that, AGS and MKN45 cells were transfected with miR-196a-3p mimics or miR-196a-3p inhibitor using the Lipofectamine 2000 kit (Thermo Fisher Scientific).

Human circPTK2 or AATK cDNA was synthesized and cloned into pcDNA3.1 vector. After that, AGS and MKN45 cells were transfected with the pcDNA3.1 control plasmid, pcDNA3.1-circPTK2 (circPTK2-OE) or pcDNA3.1 AATK (AATK-OE) using Lipofectamine 2000, followed by selection with G418.

Lentivirus-containing short hairpin RNA (shRNA) targeting circPTK2 or AATK plasmids was purchased from Hanbio (Shanghai, China). After that, 293T cells were transfected with the abovementioned lentiviral plasmids and were transduced into 293T cells to package lentivirus to infect AGS and MKN45 cells. Subsequently, the infected cells were selected by 2 µg/ml of puromycin.

### RT-PCR and RT-qPCR

A TRIzol reagent (Thermo Fisher Scientific) was used to extract total RNA. Meanwhile, genomic DNA (gDNA) was isolated using the Genomic DNA Isolation Kit (Sangon Biotech, Shanghai, China). After that, cDNA was synthesized using EntiLink™ 1st Strand cDNA Synthesis Kit (ELK Biotechnology). In addition, qPCR was performed using the EnTurbo™ SYBR Green PCR SuperMix on a Verse flow cytometry system (BD Biosciences, NJ, USA). The DreamTaq DNA Polymerase (Thermo Fisher Scientific) was used for PCR. Then, the cDNA and gDNA PCR products were analyzed by 2% agarose gel electrophoresis. β-Actin and U6 were used as internal controls. The primers are listed in **Table 1**.

**Table 1:** Primer sequences.

Name		Primer sequences
U6	Forward	5′-CTCGCTTCGGCAGCACAT-3′
	Reverse	5′-AACGCTTCACGAATTTGCGT-3′
MiR-196a-3p	Forward	5′-CGGCAACAAGAAACUGCCUGAG-3′
	Reverse	5′-CAGGCAGUUUCUUGUUGCCGUU-3′
Actin	Forward	5′-GTCCACCGCAAATGCTTCTA-3′
	Reverse	5′-TGCTGTCACCTTCACCGTTC-3′
CircPTK2	Forward	5′-GAAAGATTTCTGCCCAGCAGA-3′
	Reverse	5′-GTGATTCCATGTGAACCAGGG-3′
AATK	Forward	5′-ATGCTGGCCTGCCTGTGTTGT-3′
	Reverse	5′-AGGGGCAGGACATACACATCGG-3′

### Actinomycin D and RNase R Treatment

AGS and MKN45 cells were incubated with actinomycin D (2 μg/ml; Sigma) for 0, 6, 12, 18, and 24 h to assess the stability of circPTK2 and its linear isoform. In addition, total RNA (10 μg) was treated with RNase R (5 U/μg; Epicenter Technologies) for 30 min at 37°C, then the level of circPTK2 was detected using RT-qPCR assay.

### Cell Viability Assay

Cell viability was measured using a Cell Counting Kit-8 (CCK-8, Dojindo Laboratories, Kumamoto, Japan). Transfected AGS and MKN45 cells (5 × 10^3^ cells/well) were seeded onto 96-well plates and cultured for the indicated times. Later on, 10 µl of CCK-8 reagent was added into each well, and cells were incubated for another 2 h. Subsequently, the absorbance was measured at a wavelength of 450 nm.

### Colony Formation Assay

Transfected AGS and MKN45 cells (5 × 10^3^ cells/well) were plated onto six-well plates. After 2 weeks of incubation, cells were fixed with 4% paraformaldehyde for 20 min and then stained with 0.1% crystal violet at room temperature. After that, cell colonies were imaged and counted using a light microscope.

### Transwell Assay

Transfected AGS and MKN45 cells were suspended in 200 μl serum-free medium and placed into the upper chambers (Corning, NY, USA). Later on, the lower chambers were loaded with DMEM medium (600 μl) containing 10% FBS. After 24 h of incubation, the cells on the lower surface were fixed with 4% formaldehyde, and then stained with 0.1% crystal violet solution. After that, the stained cells were imaged using a light microscope. Transwell chambers that were coated with Matrigel (BD Biosciences) were used for the cell invasion assay.

### Animal Study

BALB/c nude mice (5–6 weeks old) were purchased from the Jingda Experimental Animal Co., Ltd. (Changsha, China). This study was approved by the First Affiliated Hospital of Soochow University and conducted according to institutional guidelines. Animals were divided into eight groups (six mice per group): group I (AGS cell)—shRNA NC, circPTK2 shRNA2, OE NC, and circPTK2 OE groups; group II (MKN45 cell)—shRNA NC, circPTK2 shRNA2, OE NC, and circPTK2 OE groups. AGS or MKN45 cells (5 × 10^6^ cells/mouse) were subcutaneously injected into the right flank of each mouse. The size of the tumor was measured every 5 days. The tumor volume was calculated by the formula: (length × width^2^)/2. At the end of the experiment, the tumor was removed, and tumor weight was measured.

### Luciferase Reporter Assay

The sequences including miR-196a-3p binding sites in the circPTK2 3′ UTRs and AATK 3′ UTR were cloned into the luciferase reporter vector pGL6-miR (Beyotime). After that, AGS or MKN45 cells were co-transfected with the luciferase plasmids and miR-196a-3p mimics for 48 h. Later on, the firefly and Renilla luciferase activities were measured by a dual-luciferase reporter assay system (Promega, Madison, USA).

### RNA Pull-Down Assay

The biotinylated circPTK2 or biotinylated miR-196a-3p probe was incubated with streptavidin magnetic beads (Thermo Fisher Scientific) for 2 h at room temperature. Later on, AGS or MKN45 cells were incubated with the magnetic beads at 4°C overnight. After that, the complex was pulled down and analyzed by RT-qPCR assay.

### Immunohistochemistry

The tumor tissues were fixed in 4% paraformaldehyde and then embedded in paraffin. Later on, tissues were sectioned (5 μm thick) and then stained with primary antibody specific for AATK (Abcam) overnight at 4°C. Images were captured by a fluorescence microscope.

### Western Blot Assay

Protein concentration was determined by the BCA kit (Pierce, Rockford, USA). After that, equal amounts of proteins (30 μg) were separated by 10% SDS-PAGE and transferred onto a PVDF membrane. Later on, the membrane was incubated with primary antibodies against STK39 (1:1,000, Abcam), AATK (1:1,000, Abcam), p-STK39 (1:1,000, Abcam), p-p38 (1:1,000, Abcam), p38 (1:1,000, Abcam), Bax (1:1,000, Abcam), Bcl-2 (1:1,000, Abcam), cleaved caspase 3 (1:1,000, Abcam), CD81 (1:1,000, Abcam), CD63 (1:1,000, Abcam), and GAPDH (1:1,000, Abcam) at 4°C overnight. Then, the membrane was incubated with horseradish peroxidase (HRP)-labeled secondary antibodies at room temperature and then visualized using the enhanced chemiluminescence reagent (Thermo Fisher Scientific).

### Co-Immunoprecipitation

Cells were transfected with pcDNA3.1-AATK or pcDNA3.1-STK39 plasmids. After that, the transfected cells were lysed using RIPA buffer, and then the cell lysates were treated with anti-AATK, anti-STK39, or anti-IgG antibodies. Later on, the samples were incubated with protein A and G Sepharose beads for 4 h at 4°C. Then, the protein binding complex was isolated and subjected to Western blot assay.

### Statistical Analysis

Data were presented as mean ± standard deviation (SD). Student’s *t*-test was applied to determine the statistical significance between two groups. Differences between three or more groups were analyzed by one-way analysis of variance (ANOVA) and Tukey’s tests. *p <*0.05 was considered statistically significant. All data were repeated in triplicate.

## Results

### Differential Expression of CircRNAs in Gastric Cancer

**Figure 1:**
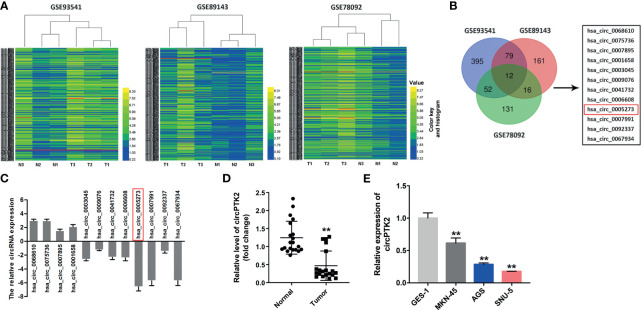
Differential expression of circular RNAs (circRNAs) in gastric cancer. **(A)** The heatmaps of the DEcircRNA profiles in gastric cancer and compared normal tissues in GSE93541, GSE89143, and GSE78092. CircRNAs in yellow indicate overexpression; circRNAs in blue indicate reduced expression. **(B)** Venn diagram of overlapping DEcircRNAs from intersection of GSE93541, GSE89143, and GSE78092 datasets. **(C)** The expressions of circRNAs in tumor tissues (*n* = 5) and normal tissues (*n* = 5) were detected with RT-qPCR. **(D)** RT-qPCR analysis of circPTK2 level in tumor tissues (*n* = 20) and normal tissues (*n* = 20). **(E)** RT-qPCR analysis of circPTK2 level in AGS, MKN45, and SNU-5 cells. **p < 0.01. The significance between two or more groups was analyzed by Student’s *t*-test or one-way ANOVA, respectively.

To identify DEcircRNAs in gastric cancer, R language was performed to analyze the circRNA expression profiles from three gastric cancer-related datasets (GSE93541, GSE89143, and GSE78092). As shown in **Figure 1A**, the heatmap showed that 538, 268, and 211 DEcircRNAs were identified in the GSE93541, GSE89143, and GSE78092 datasets, respectively. Using a Venn diagram, 12 overlapping DEcircRNAs (4 were upregulated, while 8 were downregulated) were identified in these three datasets (**Figure 1B**). To verify these results, all of these circRNAs were chosen for further confirmation in gastric cancer tissues and normal tissues using RT-qPCR (**Figure 1C**). RT-qPCR results showed that hsa_circ_0005273 (circPTK2) significantly decreased in gastric cancer tissues compared with that in normal tissues and exhibited the most significant difference between gastric cancer tissues and normal tissues (**Figures 1C, D**). Meanwhile, 95% of the total 20 of patients expressed a lower level of circPTK2 in gastric cancer tissues compared with normal tissues (**Figure 1D**). As shown in **Figure 1E**, circPTK2 expression was markedly decreased in MKN45, AGS, and SNU-5 cells compared with GES-1 cells. Thus, hsa_circ_0005273 (circPTK2) was chosen for further experiments.

**Figure 2:**
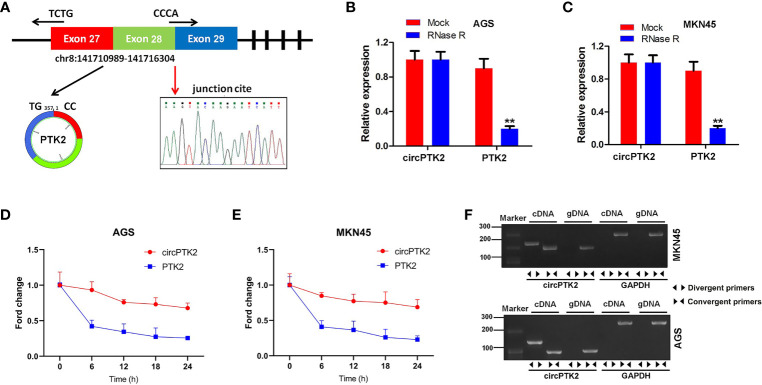
CircPTK2 is a stable circRNA from PTK2. **(A)** Schematic diagram showed the formation of circPTK2. Sanger sequencing showed the joint site of circPTK2 (red arrow). **(B, C)** The expression of linear and circRNA linear was detected with RT-qPCR, after RNase R treatment. **p < 0.05 *vs.* Mock. **(D, E)** After actinomycin treatment, the half-lives of linear and circRNAs were detected. **(F)** The existence of circPTK2 was detected in AGS and MKN45 cells by RT-qPCR with divergent or convergent primers and confirmed by gel electrophoresis.

Furthermore, we found that circPTK2 is derived from exons 27, 28, and 29 of the PTK2 gene (**Figure 2A**). In addition, Sanger sequencing verified the head-to-tail splicing in the RT-qPCR product of circPTK2 (**Figure 2A**). Next, RNase R digestion assay showed that the linear form of PTK2 was markedly decreased under the RNase R treatment, while the circular isoform was resistant to RNase R digestion, suggesting that circPTK2 harbors a loop structure (**Figures 2B, C**). Meanwhile, the stability of circPTK2 in AGS and MKN45 cells was detected using the actinomycin D assay. The data showed that the linear PTK2 mRNA transcript was less stable than circPTK2 transcript in AGS and MKN45 cells under treatment with actinomycin D (**Figures 2D, E**). Furthermore, to confirm the existence of circPTK2, we designed convergent primers to amplify PTK2 mRNA and divergent primers to amplify circPTK2. The results of PCR showed that circPTK2 was only amplified by cDNA templates from AGS and MKN45 cells using divergent primers (**Figure 2F**). To sum up, circPTK2 is decreased in gastric cancer tissues and is a stable circRNA from PTK2.

### Overexpression of CircPTK2 Inhibits Gastric Cancer Cell Proliferation and Tumor Growth

**Figure 3:**
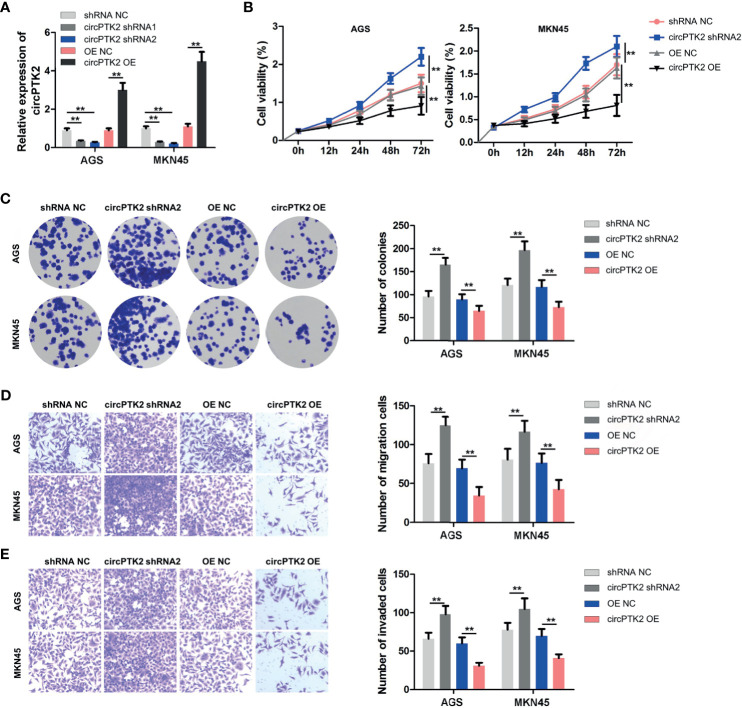
Overexpression of circPTK2 inhibits gastric cancer cell proliferation, migration, and invasion. **(A)** RT-qPCR analysis of circPTK2 level in AGS and MKN45 cells treated with circPTK2 shRNA1, circPTK2 shRNA2, or circPTK2-OE. **(B)** AGS and MKN45 cells were treated with circPTK2 shRNA2 or circPTK2-OE. Cell viability was measured by CCK-8 assay. **(C)** Cell proliferation was determined by colony formation staining assay. **(D)** Cell migration and **(E)** cell invasion were measured by transwell assays. **p < 0.01. The significance between the four groups was analyzed by one-way ANOVA.

**Figure 4:**
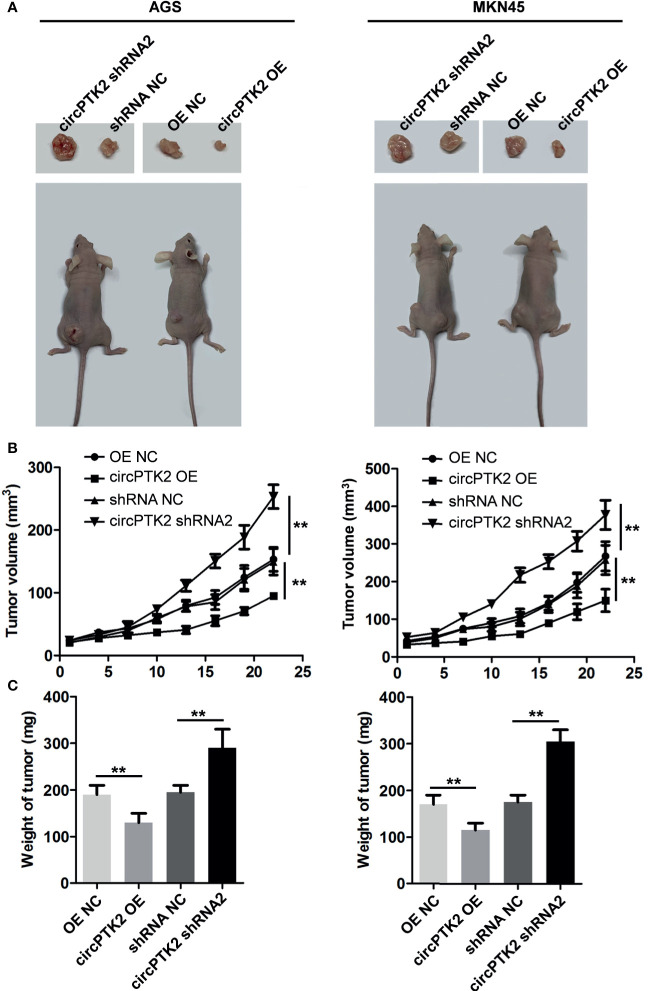
Overexpression of circPTK2 inhibits gastric cancer cell growth *in vivo*. **(A–C)** Tumor volume and tumor weight of xenograft tumors. **p < 0.01. The significance between the four groups was analyzed by one-way ANOVA.

To explore the biological role of circPTK2 in gastric cancer cells, we used shRNAs to downregulate the level of circPTK2 in gastric cancer cells (**Figure 3A**). Meanwhile, we established circPTK2 stably overexpressing gastric cancer cells *via* transfecting with circPTK2 OE plasmids (**Figure 3A**). Additionally, downregulation of circPTK2 notably promoted the viability, proliferation, migration, and invasion of AGS, MKN45, and SNU-5 cells, while circPTK2 overexpression exhibited opposite effects (**Figures 3B–E** and **Supplementary Figures S1A–C**). We further investigated the effect of circPTK2 on tumor growth *in vivo*. As shown in **Figures 4A–C**, silencing of circPTK2 markedly increased the tumor volume weight in mouse xenografts, whereas overexpression of circPTK2 obviously inhibited the tumor growth of AGS and MKN45 cells. Collectively, circPTK2 may play a tumor-suppressive role in gastric cancer *in vitro* and *in vivo*.

### CircPTK2 Acts as the Sponge of MiR-196a-3p

**Figure 5:**
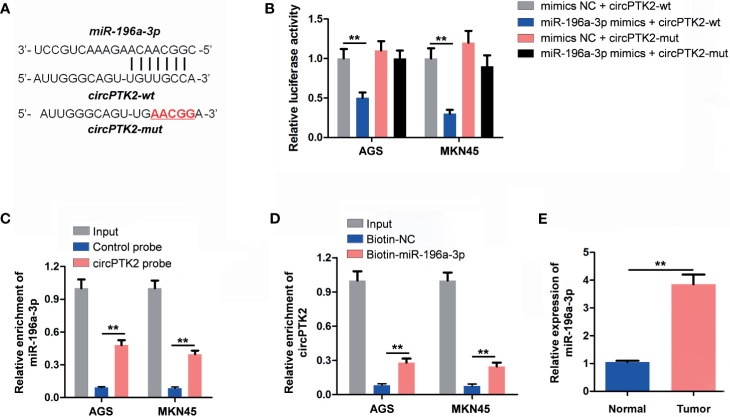
CircPTK2 acts as the sponge of miR-196a-3p. **(A)** Schematic diagram of binding sites between circPTK2 and miR-196a-3p. **(B)** Luciferase activity analysis in AGS and MKN45 cells co-transfected with the circPTK2-wt/mut vectors together with miR-196a-3p mimics or mimics control. **(C, D)** RNA pull-down assay was performed in AGS and MKN45 cells to verify the binding between circPTK2 and miR-196a-3p. **(E)** RT-qPCR analysis of miR-196a-3p level in tumor tissues and in normal tissue. **p < 0.01. The significance between two or more groups was analyzed by Student’s *t*-test or one-way ANOVA, respectively.

It has been shown that circRNAs can regulate gene expression *via* acting as miRNA sponges (24). Thus, we predicted the potential miRNAs binding to circPTK2 using the CircInteractome dataset. The data showed that miR-196a-3p functioned as the target of circPTK2 with complementary binding sites (**Figure 5A**). In addition, miR-196a-3p mimics notably reduced the luciferase activity in AGS and MKN45 cells co-transfected with circPTK2-WT; however, miR-196a-3p mimics had no effect on luciferase activity in AGS and MKN45 cells co-transfected with circPTK2-MT, suggesting that miR-196a-3p is a direct binding target of circPTK2 (**Figure 5B**). The RNA pull-down assay results showed that miR-196a-3p was pulled down by biotin-labeled circPTK2 probe in both AGS and MKN45 cells and circPTK2 was pulled down by biotin-labeled miR-196a-3p probe, indicating that miR-196a-3p directly interacted with circPTK2 (**Figures 5C, D**). Meanwhile, RT-qPCR results showed that the level miR-196a-3p was significantly increased in gastric cancer tissues (**Figure 5E**). To sum up, circPTK2 could act as a miRNA sponge for miR-196a-3p in gastric cancer.

### AATK Is a Direct Binding Target of MiR−196a-3p

**Figure 6:**
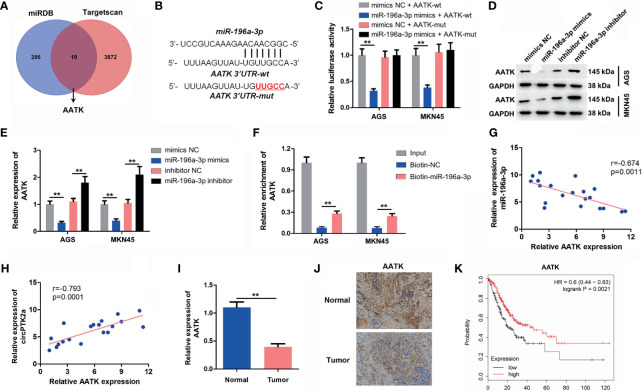
AATK is a direct binding target of miR−196a-3p. **(A)** Screen of the candidate genes that target miR-196a-3p predicted by miRDB and TargetScan. **(B)** Schematic diagram of binding sites between AATK and miR-196a-3p. **(C)** Luciferase activity analysis in AGS and MKN45 cells co-transfected with the AATK-wt/mut vectors together with miR-196a-3p mimics or mimics control. **(D, E)** Western blot analysis of AATK expression in AGS and MKN45 cells transfected with miR-196a-3p mimics or miR-196a-3p inhibitor. **(F)** RNA pull-down assay was performed in AGS and MKN45 cells to verify the binding between AATK and miR-196a-3p. **(G, H)** The Pearson’s correlation coefficients showed the correlation between AATK and miR-196a-3p or between AATK and circPTK2 in gastric cancer tissues. **(I)** RT-qPCR and **(J)** IHC analysis of AATK level in tumor tissues and in normal tissue. **(K)** Survival analysis of the correlation between AATK levels and survival rates in gastric cancer patients. **p < 0.01. The significance between two or more groups was analyzed by Student’s *t*-test or one-way ANOVA, respectively.

Two datasets miRDB and TargetScan were used to predict the potential binding targets of miR-196a-3p, and it was found that AATK might be a potential target of miR-196a-3p (**Figures 6A, B**). Additionally, miR-196a-3p mimics decreased the luciferase activity in cells co-transfected with AATK-WT (**Figure 6C**). In addition, miR-196a-3p mimics significantly downregulated the expression of AATK in AGS and MKN45 cells, while the miR-196a-3p inhibitor displayed opposite results (**Figures 6D, E**). Moreover, RNA pull-down assay showed that AATK was pulled down by biotin-labeled miR-196a-3p probe, indicating that miR-196a-3p directly interacted with AATK (**Figure 6F**). Moreover, AATK expression was negatively correlated with the expression of miR-196a-3p (*r* = −0.674, *p* < 0.05), and its expression was positively correlated with circPTK2 expression (*r* = −0.793, *p* < 0.05) (**Figures 6G, H**). Furthermore, the expression of AATK was notably downregulated in gastric cancer tissues (**Figures 6I, J**). Regarding prognosis, Kaplan–Meier curves showed that low AATK expression correlated with poor survival rate of patients of gastric cancer (**Figure 6K**). Collectively, AATK is a direct target gene of miR-196a-3p and is downregulated in gastric cancer tissues.

### Knockdown of AATK Reverses the Tumor-Suppressing Effect of CircPTK2

**Figure 7:**
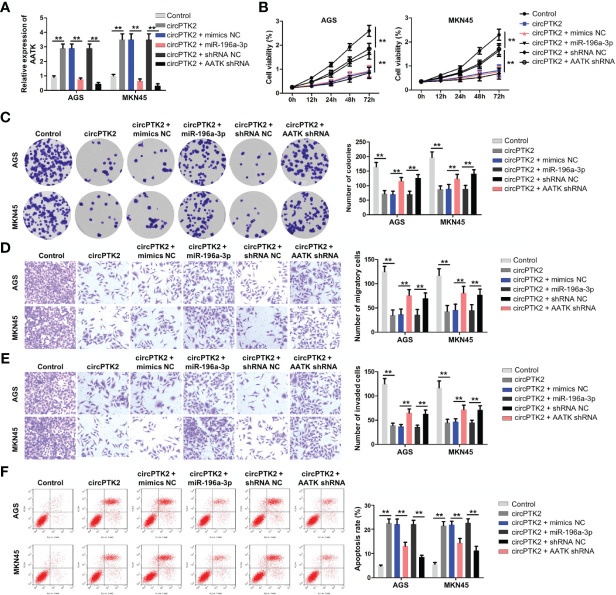
Knockdown of AATK reverses the tumor-suppressing effect of circPTK2. AGS and MKN45 cells were treated with circPTK2, circPTK2 plus miR-196a-3p mimics, or circPTK2 plus AATK shRNA. **(A)** RT-qPCR was used to detect the level of AATK in AGS and MKN45 cells. **(B)** Cell viability was measured by CCK-8 assay. **(C)** Cell proliferation was determined by colony formation staining assay. **(D)** Cell migration and **(E)** cell invasion were measured by transwell assays. **(F)** Cell apoptosis was measured by flow cytometry. **p < 0.01. The significance between five groups was analyzed by one-way ANOVA.

Next, to further confirm the interaction among circPTK2, miR-196a-3p, and AATK, rescue experiments were performed. We found that upregulation of circPTK2 notably increased the expression of AATK in AGS and MKN45 cells; however, these phenomena were reversed by miR-196a-3p mimics or by AATK knockdown (**Figure 7A**). In addition, the data of CCK-8 and colony formation showed that overexpression of circPTK2 significantly inhibited the viability and proliferation of AGS and MKN45 cells; however, these changes were reversed by miR-196a-3p mimics or AATK knockdown (**Figures 7B, C**). Meanwhile, upregulation of circPTK2 markedly suppressed the migration and invasion and triggered the apoptosis of AGS and MKN45 cells; however, these changes were reversed by miR-196a-3p mimics or AATK knockdown (**Figures 7D–F**). These data indicated that circPTK2 inhibited gastric cancer tumorigenesis by sponging miR-196a-3p, thus increasing AATK expression.

### Interaction of AATK With STK39 in Gastric Cancer

**Figure 8:**
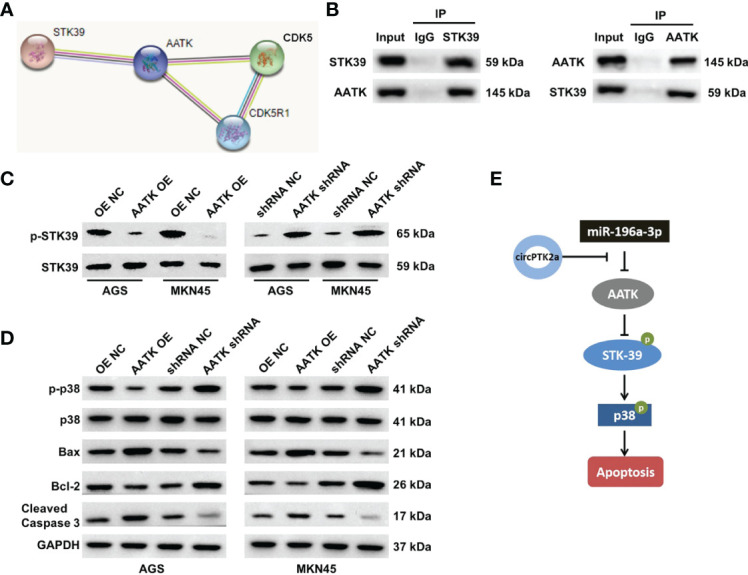
AATK interacts with STK39 and modulates the phosphorylation of p38. **(A)** Protein–protein interaction network of AATK. **(B)** Co-IP was performed to evaluate the interaction between AATK and STK39. **(C)** Western blot analysis of p-STK39 and STK39 protein expressions in AGS and MKN45 cells transfected with AATK-OE or AATK shRNA. **(D)** Western blot analysis of p-p38, p38, Bax, Bcl-2, and cleaved caspase 3 protein expressions in AGS and MKN45 cells transfected with AATK-OE or AATK shRNA. **(E)** The potential mechanism by which circPTK2 regulated the progression of gastric cancer was presented.

We further elucidated the anti-tumor mechanism of circPTK2 in gastric cancer. The protein–protein interaction network showed that AATK might interact with STK39, CDK5, and CDK5R1, respectively (**Figure 8A**). Serine/threonine kinase 39 (STK39) has been found to function as a tumor oncogene in human cancers (25, 26). In addition, Zhao et al. found that downregulation of STK39 could induce the apoptosis of renal cell carcinoma cells *via* inactivating the p38 signaling pathway (27). Thus, among the candidates for AATK-interacting proteins, STK39 was selected for further study. To explore the protein interactions between AATK and STK39, co-immunoprecipitation (co-IP) experiments were performed. The results showed that AATK could bind with STK39 in gastric cancer cell contexts (**Figure 8B**). Moreover, overexpression of AATK reduced the expressions of p-STK39, p-p38, and Bcl-2 and increased the expressions of Bax and cleaved caspase 3 in AGS and MKN45 cells (**Figures 8C, D**). Collectively, circPTK2 could induce the apoptosis of gastric cancer cells by regulating the miR-196a-3p/AATK/STK39/p39 pathways (**Figure 8E**).

## Discussion

CircRNAs have been identified as novel non-coding RNAs, which play key roles in tumor progression and affect the hallmarks of cancer (22, 28). However, the functions of circRNAs in gastric cancer remain largely unclear. In this study, we identified a cancer-associated circRNA, circPTK2, originating from exons 27, 28, and 29 of its host gene PTK2, and found that circPTK2 was significantly downregulated in gastric cancer tissues. In addition, upregulation of circPTK2 could inhibit gastric cancer cell proliferation, migration, and invasion by targeting the miR-196a-3p/AATK axis. In contrast, Yu et al. found that circPTK2 could promote gastric cancer cell proliferation by sponging miR-139-3p (29). These results suggested that circPTK2 might function as an oncogene or tumor-suppressor gene in gastric cancer.

Recently, circRNAs have been proven to exert various biological functions *via* acting as miRNA sponges (30). To determine whether circPTK2 could regulate gastric cancer progression *via* sponging miRNAs, the CircInteractome dataset was used to predict the potential miRNAs. We found that miR-196a-3p might be sponged by circPTK2, which was verified by luciferase reporter assay and RNA pull-down assay. Rescue experiments revealed that overexpression of miR-196a-3p significantly attenuated the anti-cancer role of circPTK2 in gastric cancer, suggesting that circPTK2 regulated the progression of gastric cancer *via* sponging miR-196a-3p. Besides, circRNAs could sponge miRNAs and prevent them from interacting with target mRNA, which in turn upregulate target gene expression (31). In this study, we found that AATK is a downstream target gene of miR-196a-3p. Ma et al. showed that the expression of AATK was downregulated in metastatic melanoma cells, and overexpression of AATK could suppress the migration and trigger the apoptosis of melanoma cells (32). In agreement with a previous study, we found that AATK was downregulated in gastric cancer cells. In addition, overexpression of circPTK2 markedly upregulated the expression of AATK in gastric cancer cells, suggesting that circPTK2 could interact with miR-196a-3p and function as a miRNA sponge to regulate AATK expression.

Importantly, the association of AATK with the development of gastric cancer has not been described. Bioinformatics analysis indicated that AATK might interact with STK39 (also known as SPAK), which was verified by co-IP assay. Evidence has shown that STK39 could promote cervical cancer progression *via* the NF-κB/p38 MAPK/MMP2 pathway (33). Our data indicated that overexpression of AATK notably downregulated the expressions of p-STK39 and p-p38 in gastric cancer cells, suggesting that AATK might inhibit gastric cancer progression *via* inactivating the STK39/p38 signaling pathway. These data indicated that circPTK2 can sponge miR-196a-3p and upregulate miR-196a-3p targeting gene AATK, thereby inactivating the STK39/p38 signaling pathway.

## Conclusion

In summary, we found that circPTK2 might serve as a tumor-suppressive circRNA. Mechanistically, circPTK2 could suppress the proliferation, migration, and invasion of gastric cancer cells through directly binding to miR-196a-3p and subsequently decrease the inhibiting ability of miR-196a-3p on AATK. These data indicated that exosomal circPTK2 may be a therapeutic target in gastric cancer.

## Data Availability Statement

The raw data supporting the conclusions of this article will be made available by the authors, without undue reservation.

## Ethics Statement

The studies involving human participants were reviewed and approved by the First Affiliated Hospital of Soochow University. The patients/participants provided their written informed consent to participate in this study. The animal study was reviewed and approved by the First Affiliated Hospital of Soochow University and conducted according to institutional guidelines.

## Author Contributions

LG and TX made major contributions to the conception, design, and manuscript drafting of this study. MQ, XX, and LJ were responsible for data acquisition, data analysis, data interpretation, and manuscript revision. XZ made substantial contributions to the conception and design of the study and revised the manuscript. All authors agreed to be accountable for all aspects of the work. All authors contributed to the article and approved the submitted version.

## Funding

This study received funding from the National Natural Science Foundation of China (Grant No. 81974375).

## Conflict of Interest

The authors declare that the research was conducted in the absence of any commercial or financial relationships that could be construed as a potential conflict of interest.

## Publisher’s Note

All claims expressed in this article are solely those of the authors and do not necessarily represent those of their affiliated organizations, or those of the publisher, the editors and the reviewers. Any product that may be evaluated in this article, or claim that may be made by its manufacturer, is not guaranteed or endorsed by the publisher.

## Supplementary Material

Supplementary Figure 1 Overexpression of circPTK2 inhibits SNU-5 cell proliferation, migration and invasion. **(A)** SNU-5 cells were treated with circPTK2 shRNA2 or circPTK2-OE. Cell viability was measured by CCK-8 assay. **(B)** Cell proliferation was determined by colony formation staining assay. **(C)** Cell migration and cell invasion were measured by transwell assays. ***p* < 0.01. The significance between four groups was analyzed by one-way ANOVA.

## References

[1] Kang YK Cho H. Perioperative FLOT: New Standard for Gastric Cancer? Lancet (2019) 393(10184):1914–6. doi: 10.1016/s0140-6736(18)33189-1 30982685

[2] Yang HK Berlth F. Gastric Cancer Surgery: The Importance of Technique and Not Only the Extent of Lymph Node Dissection. Lancet Oncol (2019) 20(3):329–31. doi: 10.1016/s1470-2045(19)30073-7 30842044

[3] McCaw ZR Kim DH Tian L Fu H Wei LJ. Trifluridine/tipiracil in Metastatic Gastric Cancer. Lancet Oncol (2019) 20(1):e8. doi: 10.1016/s1470-2045(18)30908-2 30614483 PMC7017585

[4] Zeraati H Amiri Z. Estimating Postoperative Survival of Gastric Cancer Patients and Factors Affecting it in Iran: Based on a TNM-7 Staging System. Acta Med Iran (2016) 54(2):114–8. 26997598

[5] Katai H Mizusawa J Katayama H Morita S Yamada T Bando E. Survival Outcomes After Laparoscopy-Assisted Distal Gastrectomy *Versus* Open Distal Gastrectomy With Nodal Dissection for Clinical Stage IA or IB Gastric Cancer (JCOG0912): A Multicentre, Non-Inferiority, Phase 3 Randomised Controlled Trial. Lancet Gastroenterol Hepatol (2020) 5(2):142–51. doi: 10.1016/s2468-1253(19)30332-2 31757656

[6] Roviello G Generali D. Pertuzumab Therapy for HER2-Positive Metastatic Gastric or Gastro-Oesophageal Junction Cancer. Lancet Oncol (2018) 19(10):1270–2. doi: 10.1016/s1470-2045(18)30512-6 30217673

[7] Song Z Wu Y Yang J Yang D Fang X. Progress in the Treatment of Advanced Gastric Cancer. Tumour Biol (2017) 39(7):1010428317714626. doi: 10.1177/1010428317714626 28671042

[8] Patop IL Wüst S Kadener S. Past, Present, and Future of circRNAs. EMBO J (2019) 38(16):e100836. doi: 10.15252/embj.2018100836 31343080 PMC6694216

[9] Li X Yang L Chen LL. The Biogenesis, Functions, and Challenges of Circular RNAs. Mol Cell (2018) 71(3):428–42. doi: 10.1016/j.molcel.2018.06.034 30057200

[10] Greene J Baird AM Brady L Lim M Gray SG McDermott R. Circular RNAs: Biogenesis, Function and Role in Human Diseases. Front Mol Biosci (2017) 4:38. doi: 10.3389/fmolb.2017.00038 28634583 PMC5459888

[11] Li J Yang J Zhou P Le Y Zhou C Wang S. Circular RNAs in Cancer: Novel Insights Into Origins, Properties, Functions and Implications. Am J Cancer Res (2015) 5(2):472–80. PMC4396047 25973291

[12] Kristensen LS Hansen TB Venø MT Kjems J. Circular RNAs in Cancer: Opportunities and Challenges in the Field. Oncogene (2018) 37(5):555–65. doi: 10.1038/onc.2017.361 PMC5799710 28991235

[13] Verduci L Strano S Yarden Y Blandino G. The circRNA-microRNA Code: Emerging Implications for Cancer Diagnosis and Treatment. Mol Oncol (2019) 13(4):669–80. doi: 10.1002/1878-0261.12468 PMC6441890 30719845

[14] Li R Jiang J Shi H Qian H Zhang X Xu W. CircRNA: A Rising Star in Gastric Cancer. Cell Mol Life Sci (2020) 77(9):1661–80. doi: 10.1007/s00018-019-03345-5 PMC11104848 31659415

[15] Zeng K Chen X Xu M Liu X Hu X Xu T. CircHIPK3 Promotes Colorectal Cancer Growth and Metastasis by Sponging miR-7. Cell Death Dis (2018) 9(4):417. doi: 10.1038/s41419-018-0454-8 29549306 PMC5856798

[16] Qadir MI Faheem A. miRNA: A Diagnostic and Therapeutic Tool for Pancreatic Cancer. Crit Rev Eukaryot Gene Expr (2017) 27(3):197–204. doi: 10.1615/CritRevEukaryotGeneExpr.2017019494 29199604

[17] García-Sancha N Corchado-Cobos R Pérez-Losada J Cañueto J. MicroRNA Dysregulation in Cutaneous Squamous Cell Carcinoma. Int J Mol Sci (2019) 20(9):2181. doi: 10.3390/ijms20092181 PMC6540078 31052530

[18] Pfeffer SR Yang CH Pfeffer LM. The Role of miR-21 in Cancer. Drug Dev Res (2015) 76(6):270–7. doi: 10.1002/ddr.21257 26082192

[19] Ali Syeda Z Langden SSS Munkhzul C Lee M Song SJ. Regulatory Mechanism of MicroRNA Expression in Cancer. Int J Mol Sci (2020) 21(5):1723. doi: 10.3390/ijms21051723 PMC7084905 32138313

[20] Dong W Bi J Liu H Yan D He Q Zhou Q. Circular RNA ACVR2A Suppresses Bladder Cancer Cells Proliferation and Metastasis Through miR-626/EYA4 Axis. Mol Cancer (2019) 18(1):95. doi: 10.1186/s12943-019-1025-z 31101108 PMC6524247

[21] Lu Q Liu T Feng H Yang R Zhao X Chen W. Circular RNA Circslc8a1 Acts as a Sponge of miR-130b/miR-494 in Suppressing Bladder Cancer Progression *via* Regulating PTEN. Mol Cancer (2019) 18(1):111. doi: 10.1186/s12943-019-1040-0 31228937 PMC6588875

[22] Zhang X Wang S Wang H Cao J Huang X Chen Z. Circular RNA Circnrip1 Acts as a microRNA-149-5p Sponge to Promote Gastric Cancer Progression *via* the AKT1/mTOR Pathway. Mol Cancer (2019) 18(1):20. doi: 10.1186/s12943-018-0935-5 30717751 PMC6360801

[23] Luo Z Rong Z Zhang J Zhu Z Yu Z Li T. Circular RNA Circccdc9 Acts as a miR-6792-3p Sponge to Suppress the Progression of Gastric Cancer Through Regulating CAV1 Expression. Mol Cancer (2020) 19(1):86. doi: 10.1186/s12943-020-01203-8 32386516 PMC7210689

[24] Dudekula DB Panda AC Grammatikakis I De S Abdelmohsen K Gorospe M. CircInteractome: A Web Tool for Exploring Circular RNAs and Their Interacting Proteins and microRNAs. RNA Biol (2016) 13(1):34–42. doi: 10.1080/15476286.2015.1128065 26669964 PMC4829301

[25] Li Z Zhu W Xiong L Yu X Chen X Lin Q. Role of High Expression Levels of STK39 in the Growth, Migration and Invasion of Non-Small Cell Type Lung Cancer Cells. Oncotarget (2016) 7(38):61366–77. doi: 10.18632/oncotarget.11351 PMC5308657 27542260

[26] Huang T Zhou Y Cao Y Tao J Zhou ZH Hang DH. STK39, Overexpressed in Osteosarcoma, Regulates Osteosarcoma Cell Invasion and Proliferation. Oncol Lett (2017) 14(4):4599–604. doi: 10.3892/ol.2017.6728 PMC5592870 28943960

[27] Zhao Q Zhu Y Liu L Wang H Jiang S Hu X. STK39 Blockage by RNA Interference Inhibits the Proliferation and Induces the Apoptosis of Renal Cell Carcinoma. Onco Targets Ther (2018) 11:1511–9. doi: 10.2147/ott.S153806 PMC5860137 29588603

[28] Shan C Zhang Y Hao X Gao J Chen X Wang K. Biogenesis, Functions and Clinical Significance of circRNAs in Gastric Cancer. Mol Cancer (2019) 18(1):136. doi: 10.1186/s12943-019-1069-0 31519189 PMC6743094

[29] Yu D Zhang C. Circular RNA PTK2 Accelerates Cell Proliferation and Inhibits Cell Apoptosis in Gastric Carcinoma *via* miR-139-3p. Dig Dis Sci (2021) 66(5):1499–509. doi: 10.1007/s10620-020-06358-4 32504353

[30] Panda AC. Circular RNAs Act as miRNA Sponges. Adv Exp Med Biol (2018) 1087:67–79. doi: 10.1007/978-981-13-1426-1_6 30259358

[31] Zheng Q Bao C Guo W Li S Chen J Chen B. Circular RNA Profiling Reveals an Abundant Circhipk3 That Regulates Cell Growth by Sponging Multiple miRNAs. Nat Commun (2016) 7:11215. doi: 10.1038/ncomms11215 27050392 PMC4823868

[32] Ma S Rubin BP. Apoptosis-Associated Tyrosine Kinase 1 Inhibits Growth and Migration and Promotes Apoptosis in Melanoma. Lab Invest (2014) 94(4):430–8. doi: 10.1038/labinvest.2014.13 24589855

[33] Chiu MH Liu HS Wu YH Shen MR Chou CY. SPAK Mediates KCC3-Enhanced Cervical Cancer Tumorigenesis. FEBS J (2014) 281(10):2353–65. doi: 10.1111/febs.12787 24655550

