# Supplementary figures and images for: CircPTK2 Suppresses the Progression of Gastric Cancer by Targeting the MiR-196a-3p/AATK Axis (part 1 of 2)
Source: Front Oncol. 2021 Sep 15;11:706415. doi: 10.3389/fonc.2021.706415 (PMC8479173; doi:10.3389/fonc.2021.706415)

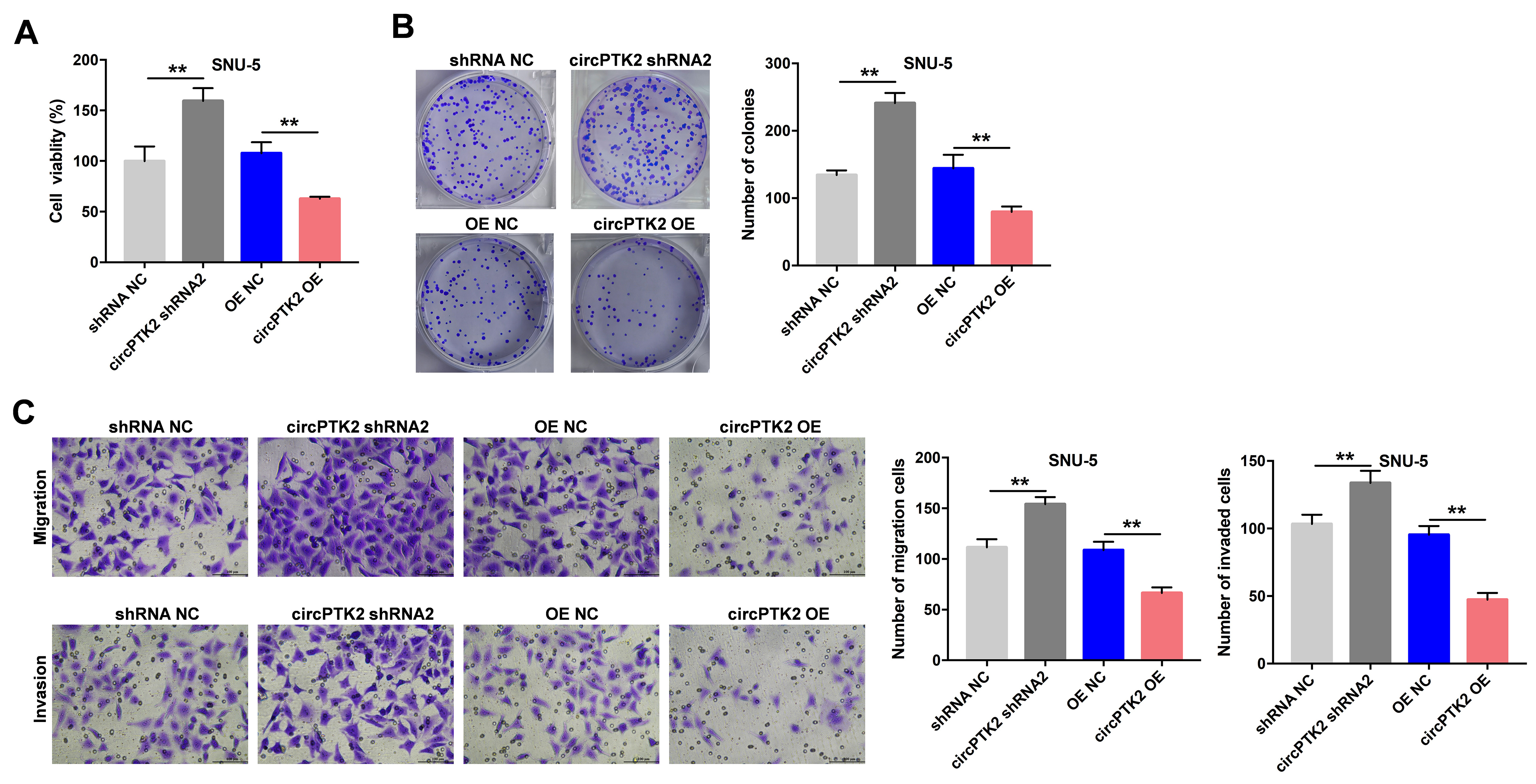

Supplement: Supplementary Figure 1 — Overexpression of circPTK2 inhibits SNU-5 cell proliferation, migration and invasion. (A) SNU-5 cells were treated with circPTK2 shRNA2 or circPTK2-OE. Cell viability was measured by CCK-8 assay. (B) Cell proliferation was determined by colony formation staining assay. (C) Cell migration and cell invasion were measured by transwell assays. **p < 0.01. The significance between four groups was analyzed by one-way ANOVA. [file Image_1.jpeg]

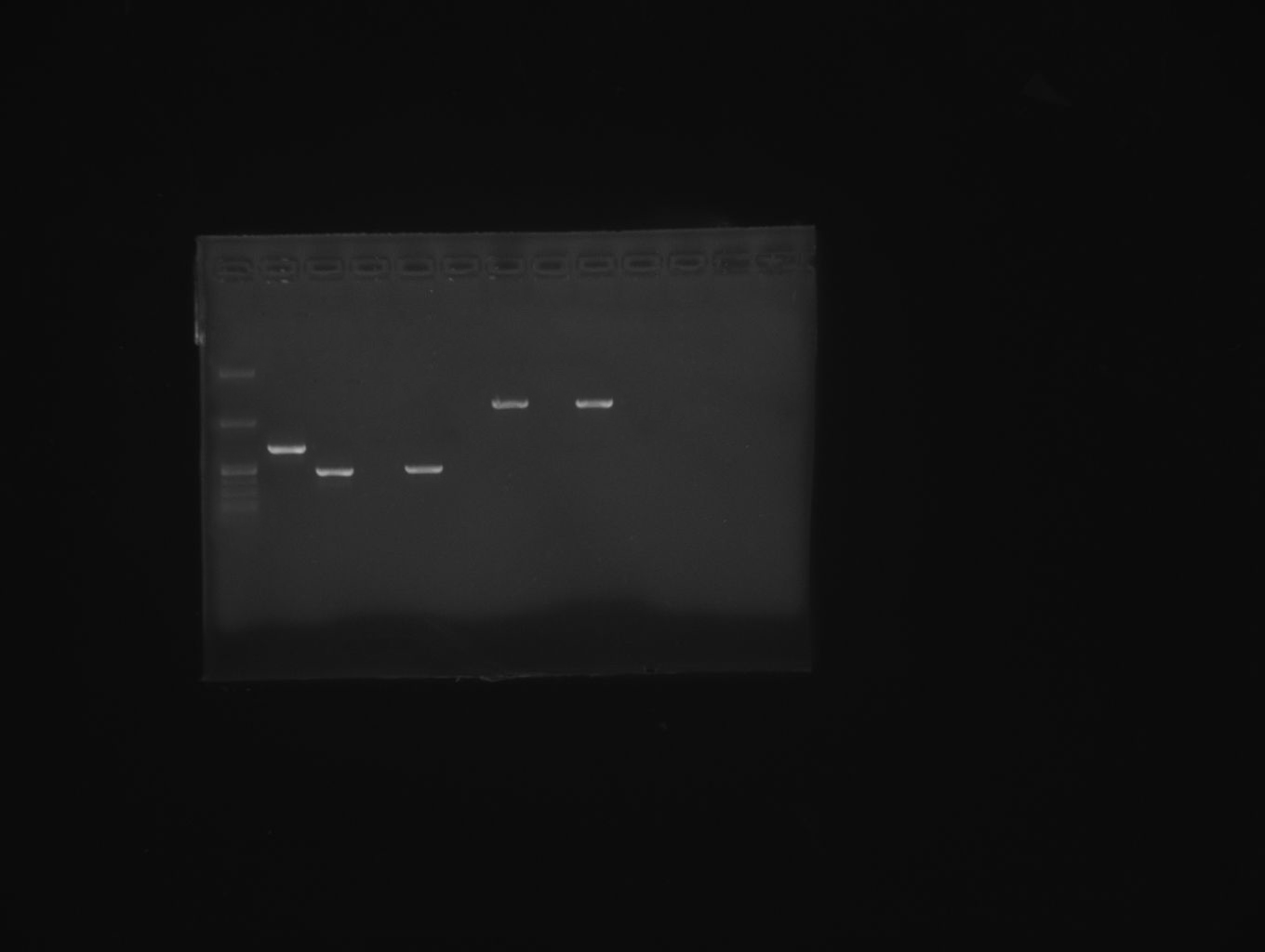

Supplement: Supplementary file 2 [file DataSheet_1.zip › Fig 2/Figure 2F/circPTK2--AGS.tif]

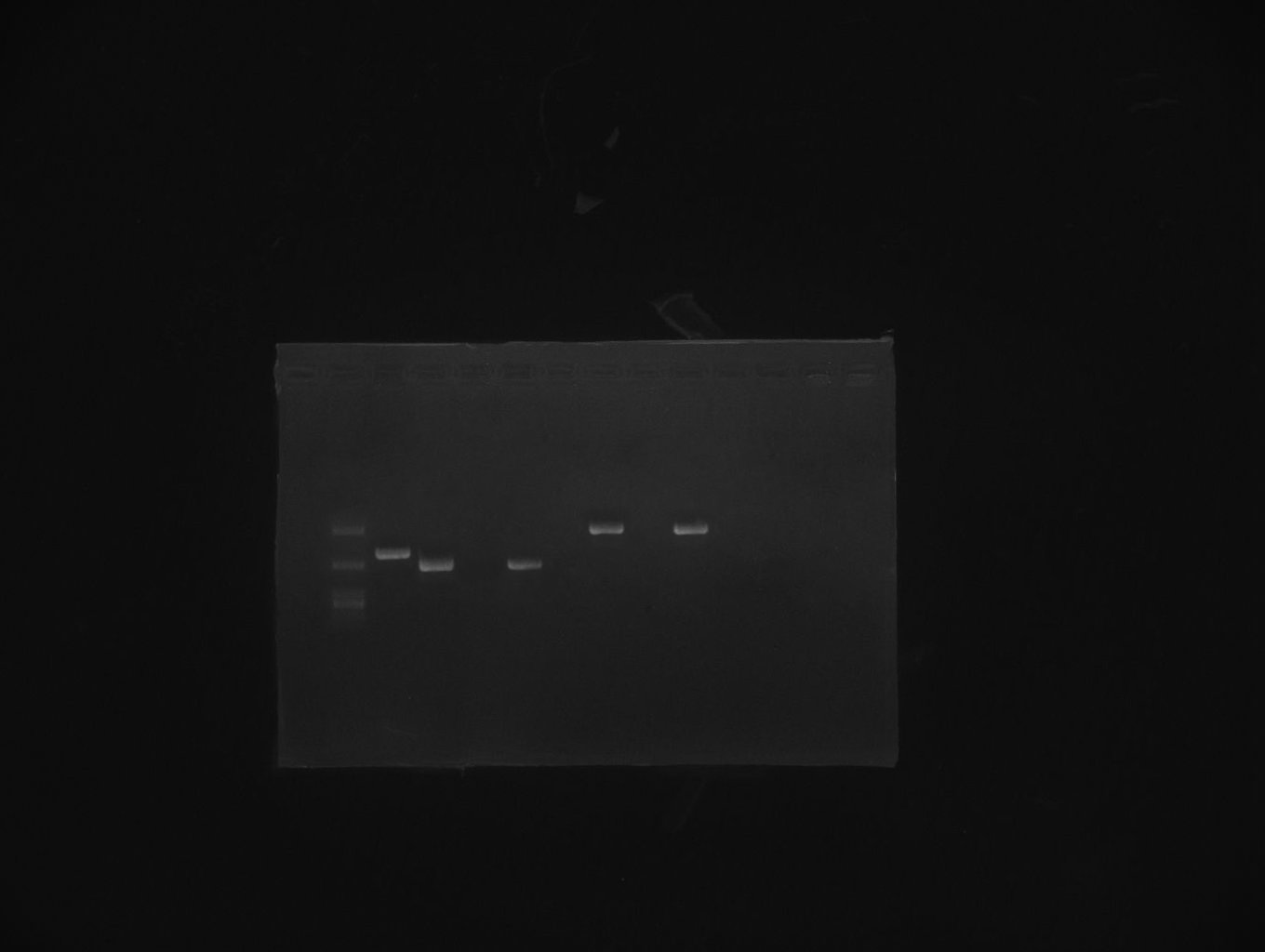

Supplement: Supplementary file 2 [file DataSheet_1.zip › Fig 2/Figure 2F/circPTK2--MKN45.tif]

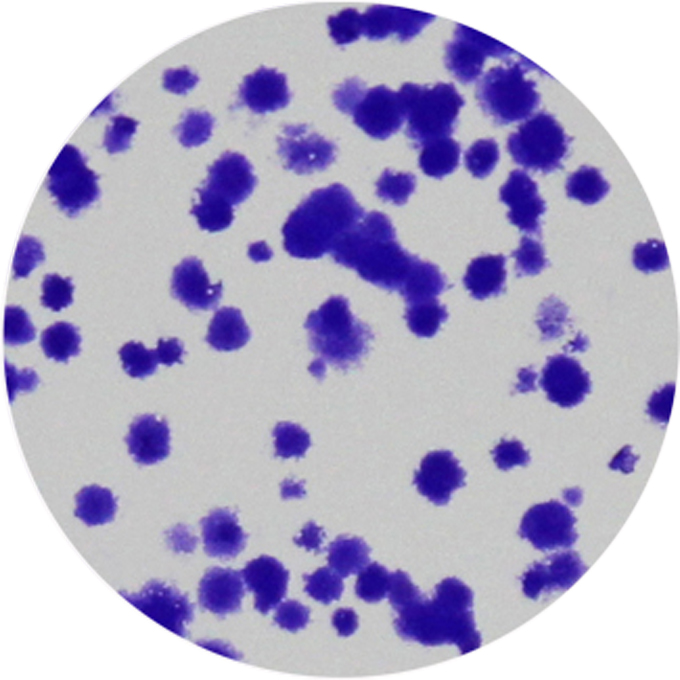

Supplement: Supplementary file 3 [file DataSheet_2.zip › Fig 3/Figure 3C colony formation/OE NC-AGS.jpg]

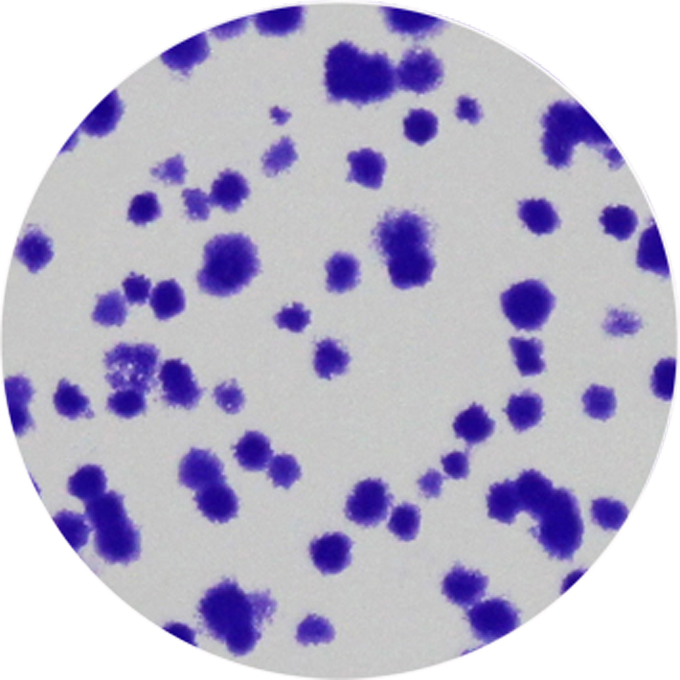

Supplement: Supplementary file 3 [file DataSheet_2.zip › Fig 3/Figure 3C colony formation/OE NC-MKN45.jpg]

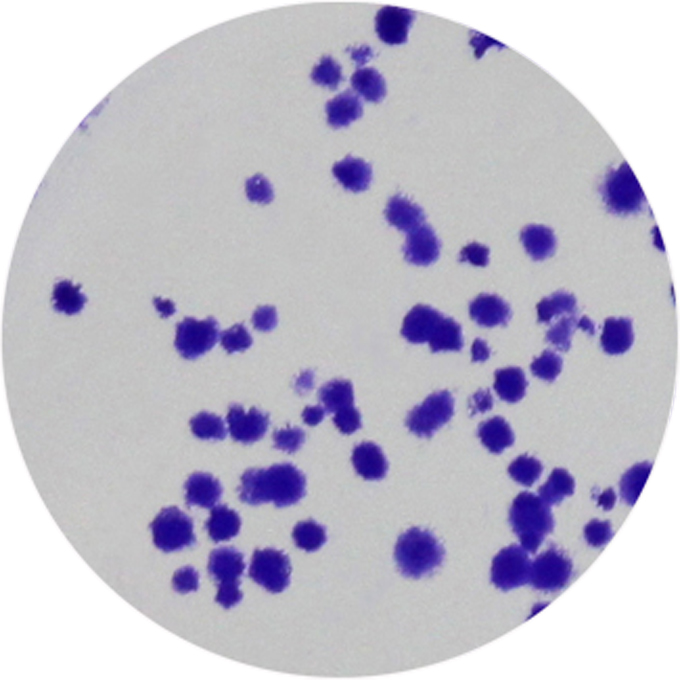

Supplement: Supplementary file 3 [file DataSheet_2.zip › Fig 3/Figure 3C colony formation/circPTK2 OE-AGS.jpg]

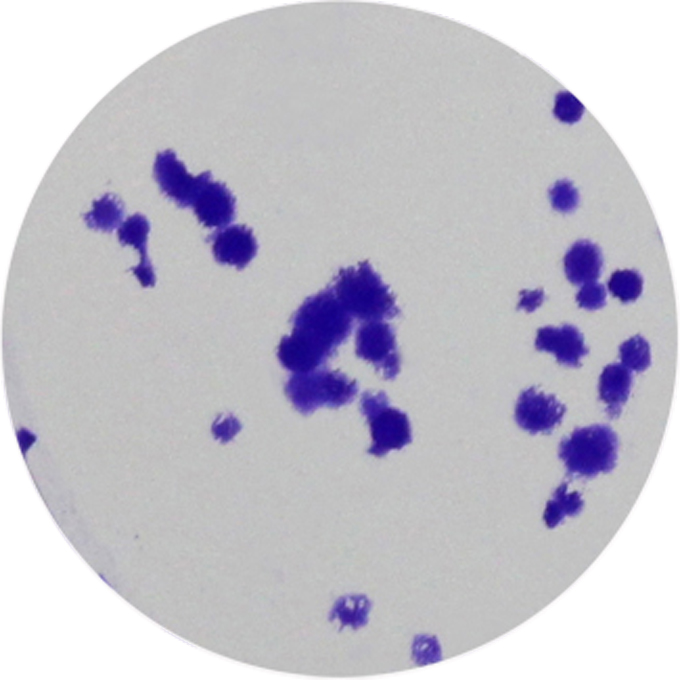

Supplement: Supplementary file 3 [file DataSheet_2.zip › Fig 3/Figure 3C colony formation/circPTK2 OE-MKN45.jpg]

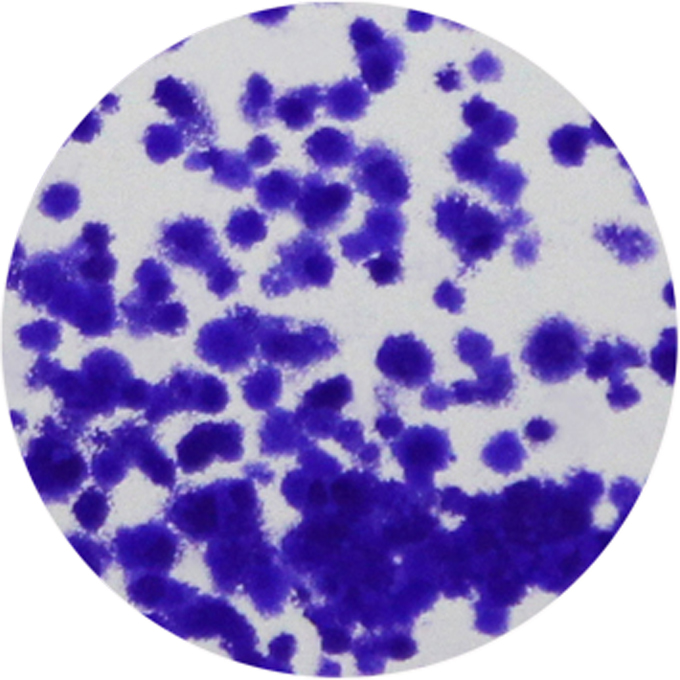

Supplement: Supplementary file 3 [file DataSheet_2.zip › Fig 3/Figure 3C colony formation/circPTK2 shRNA2-AGS.jpg]

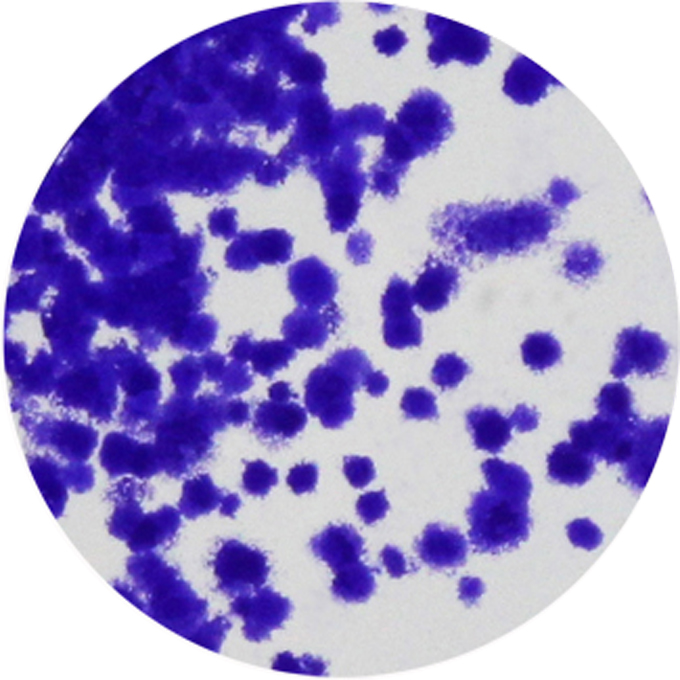

Supplement: Supplementary file 3 [file DataSheet_2.zip › Fig 3/Figure 3C colony formation/circPTK2 shRNA2-MKN45.jpg]

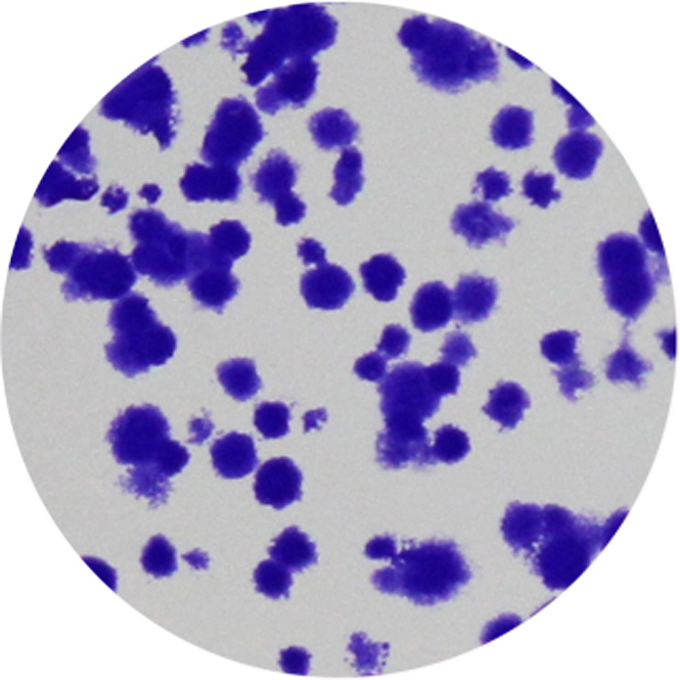

Supplement: Supplementary file 3 [file DataSheet_2.zip › Fig 3/Figure 3C colony formation/shRNA NC-AGS.jpg]

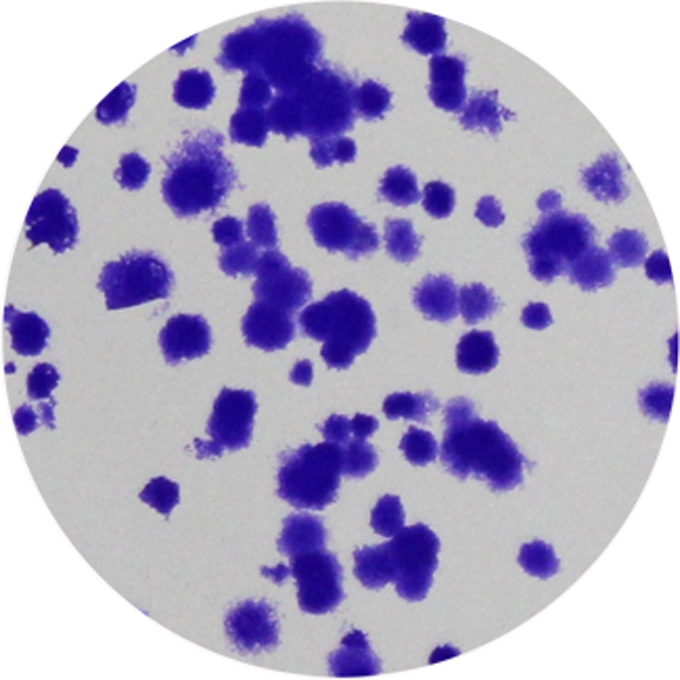

Supplement: Supplementary file 3 [file DataSheet_2.zip › Fig 3/Figure 3C colony formation/shRNA NC-MKN45.jpg]

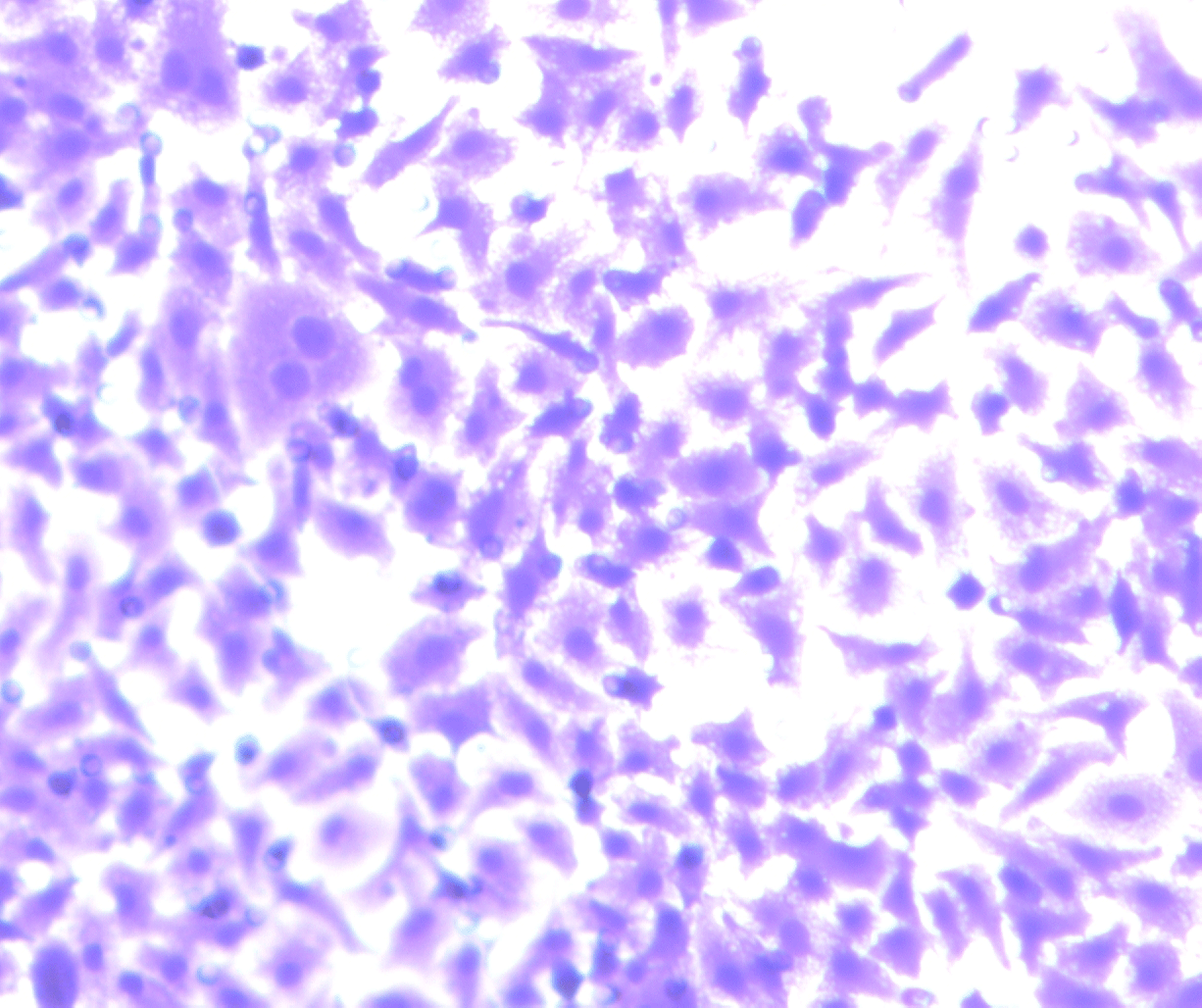

Supplement: Supplementary file 3 [file DataSheet_2.zip › Fig 3/Figure 3D and 3E transwell/invasion/Figure 3E AGS/OE NC.gif]

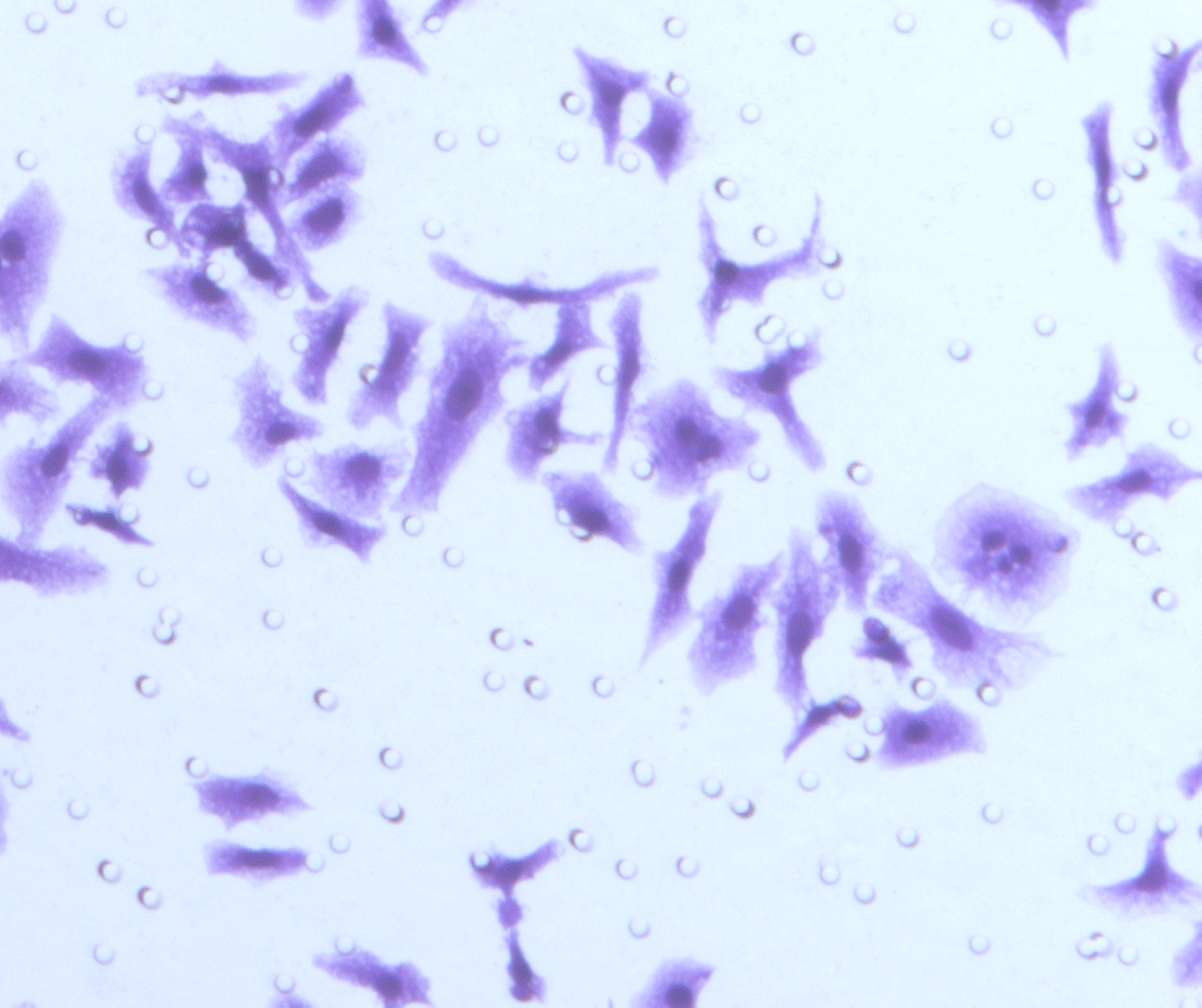

Supplement: Supplementary file 3 [file DataSheet_2.zip › Fig 3/Figure 3D and 3E transwell/invasion/Figure 3E AGS/circPTK2 OE.gif]

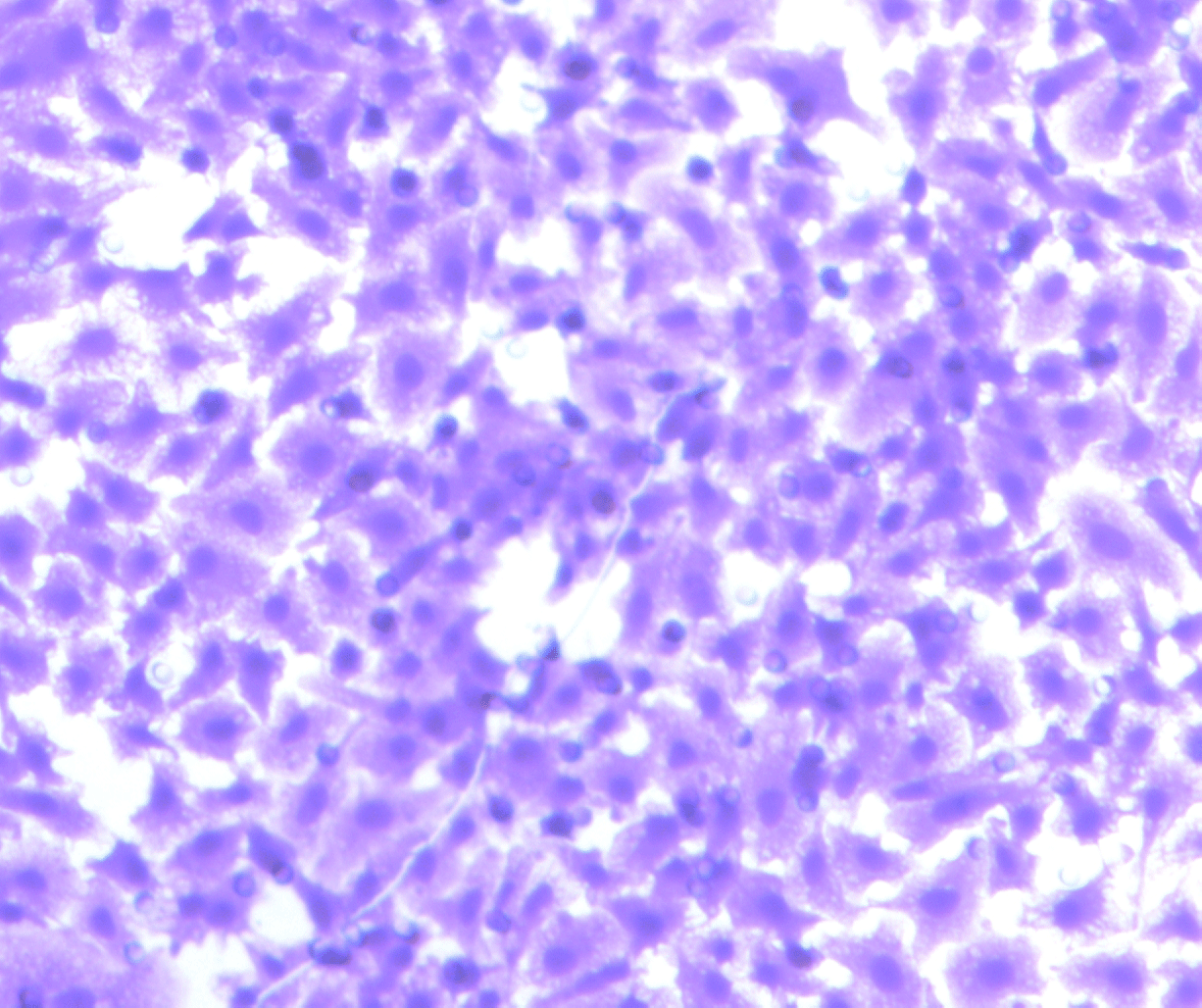

Supplement: Supplementary file 3 [file DataSheet_2.zip › Fig 3/Figure 3D and 3E transwell/invasion/Figure 3E AGS/circPTK2 shRNA2.gif]

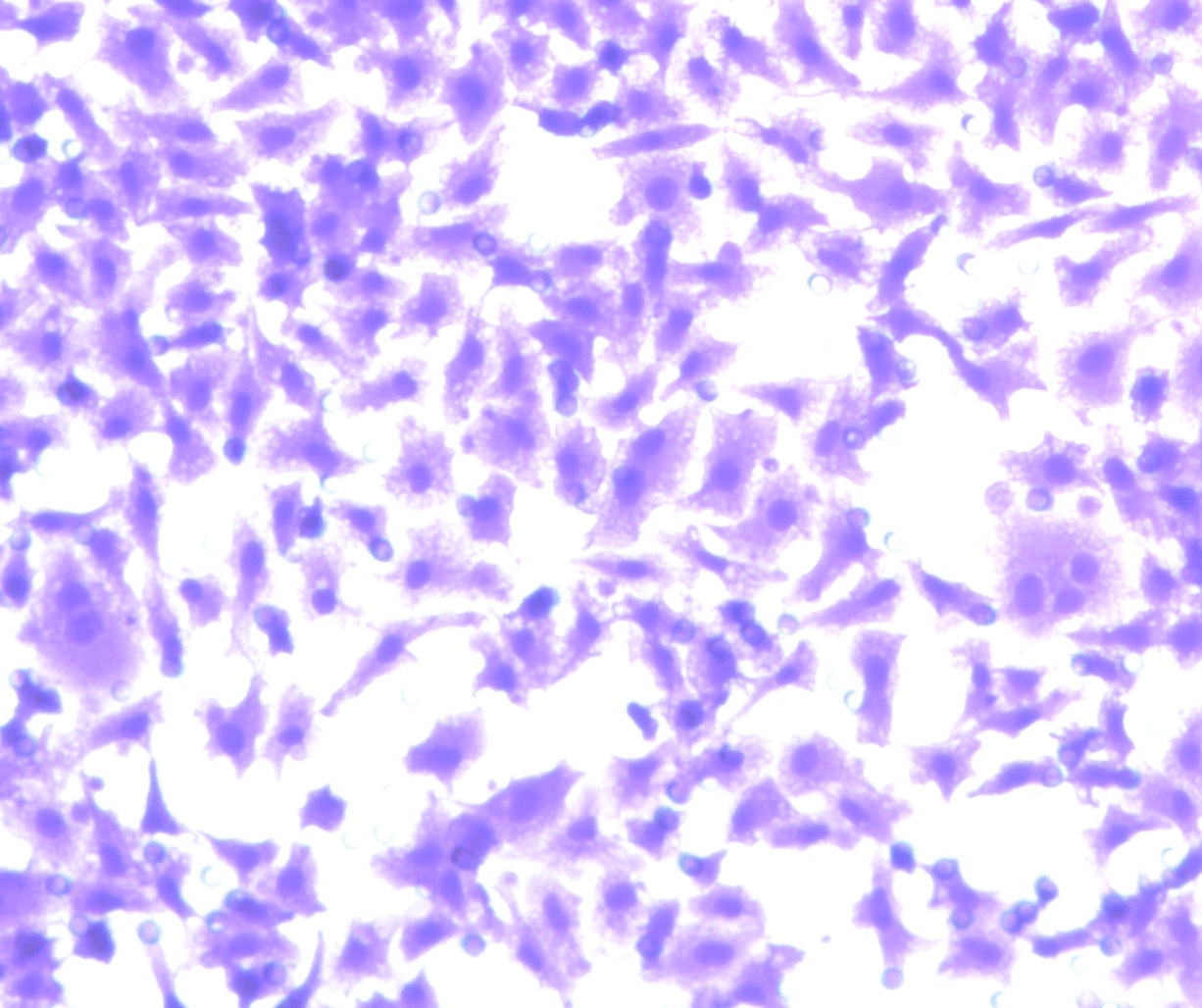

Supplement: Supplementary file 3 [file DataSheet_2.zip › Fig 3/Figure 3D and 3E transwell/invasion/Figure 3E AGS/shRNA NC.gif]

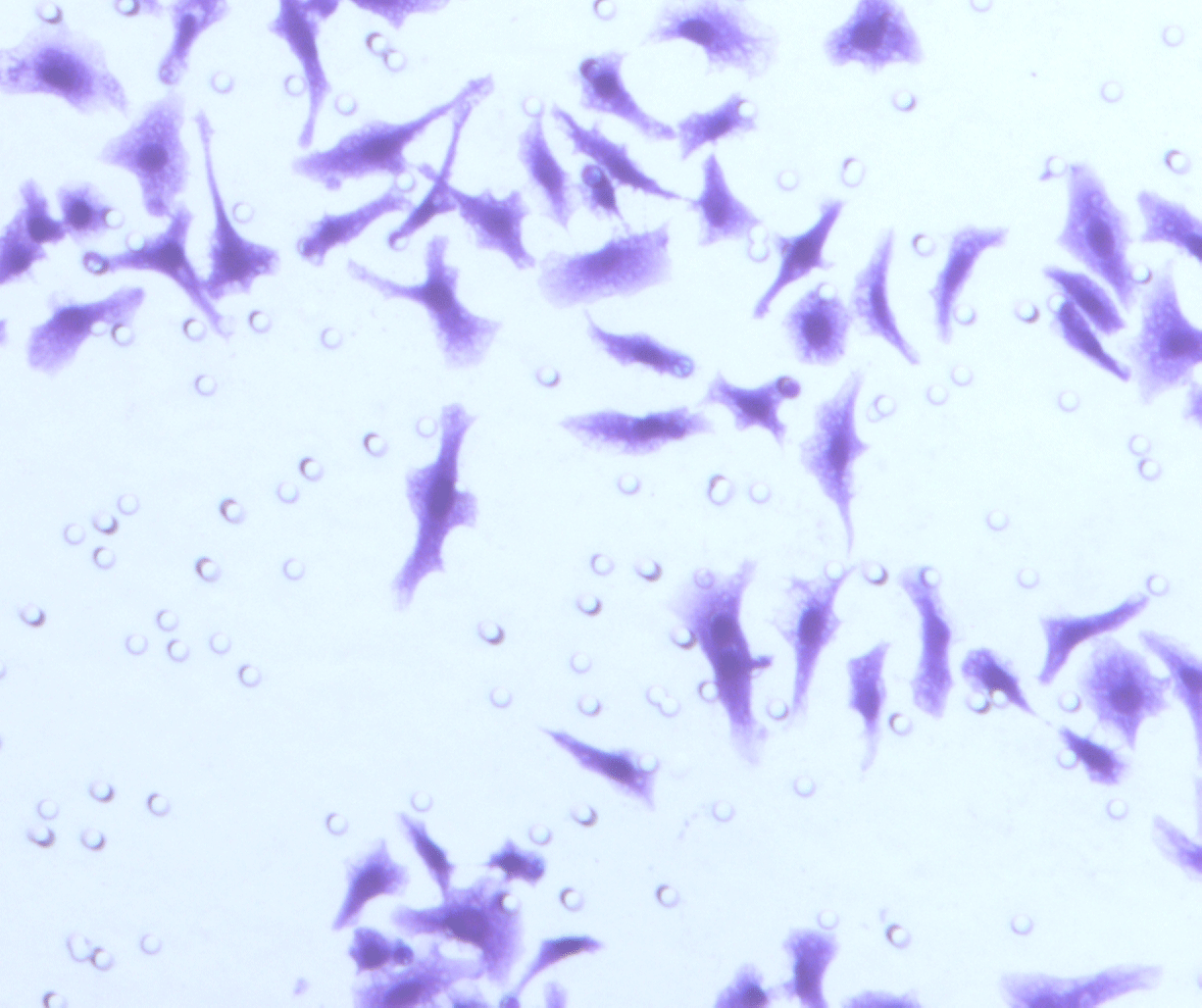

Supplement: Supplementary file 3 [file DataSheet_2.zip › Fig 3/Figure 3D and 3E transwell/invasion/Figure 3E MKN45/ circPTK2 OE.gif]

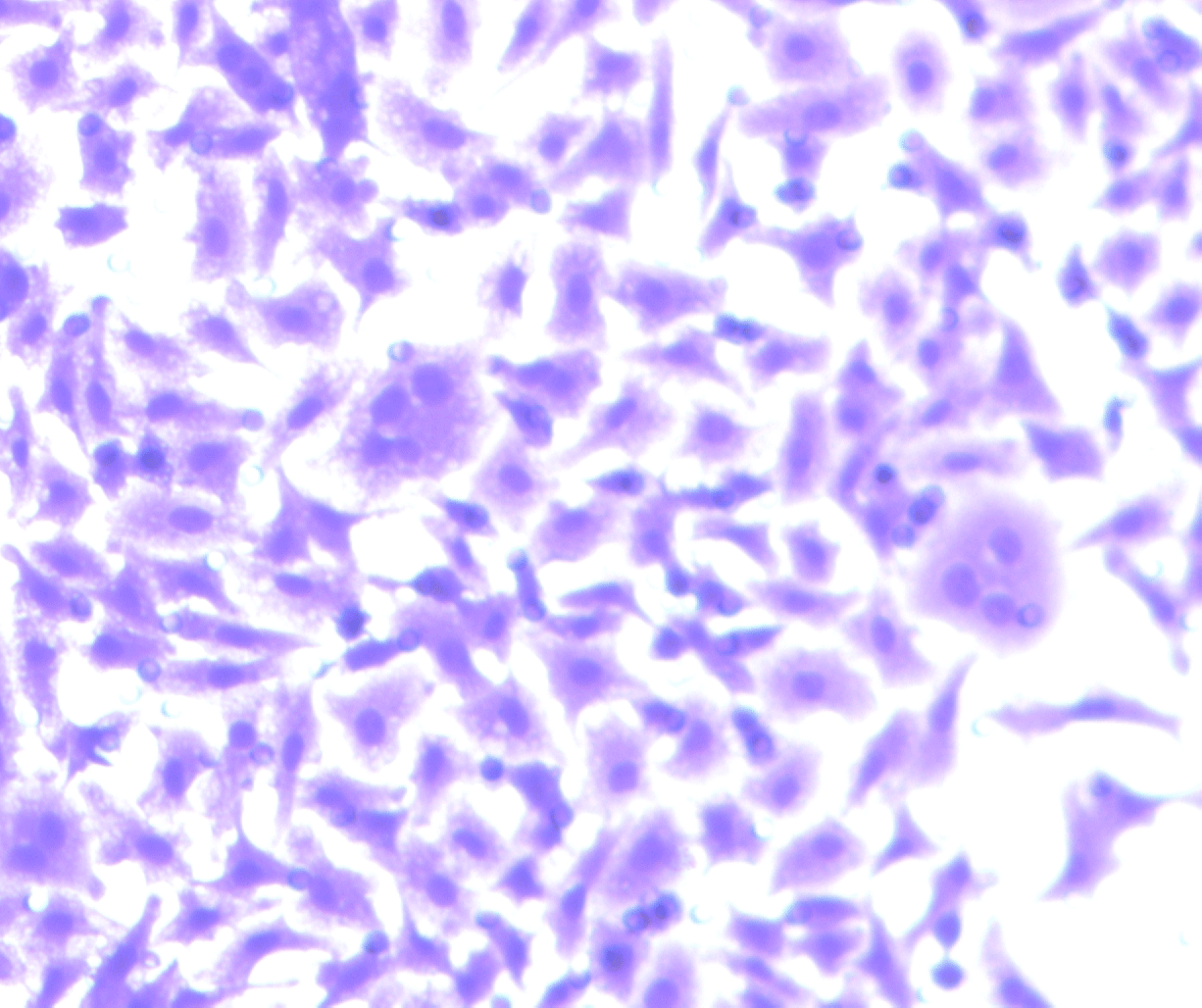

Supplement: Supplementary file 3 [file DataSheet_2.zip › Fig 3/Figure 3D and 3E transwell/invasion/Figure 3E MKN45/OE NC.gif]

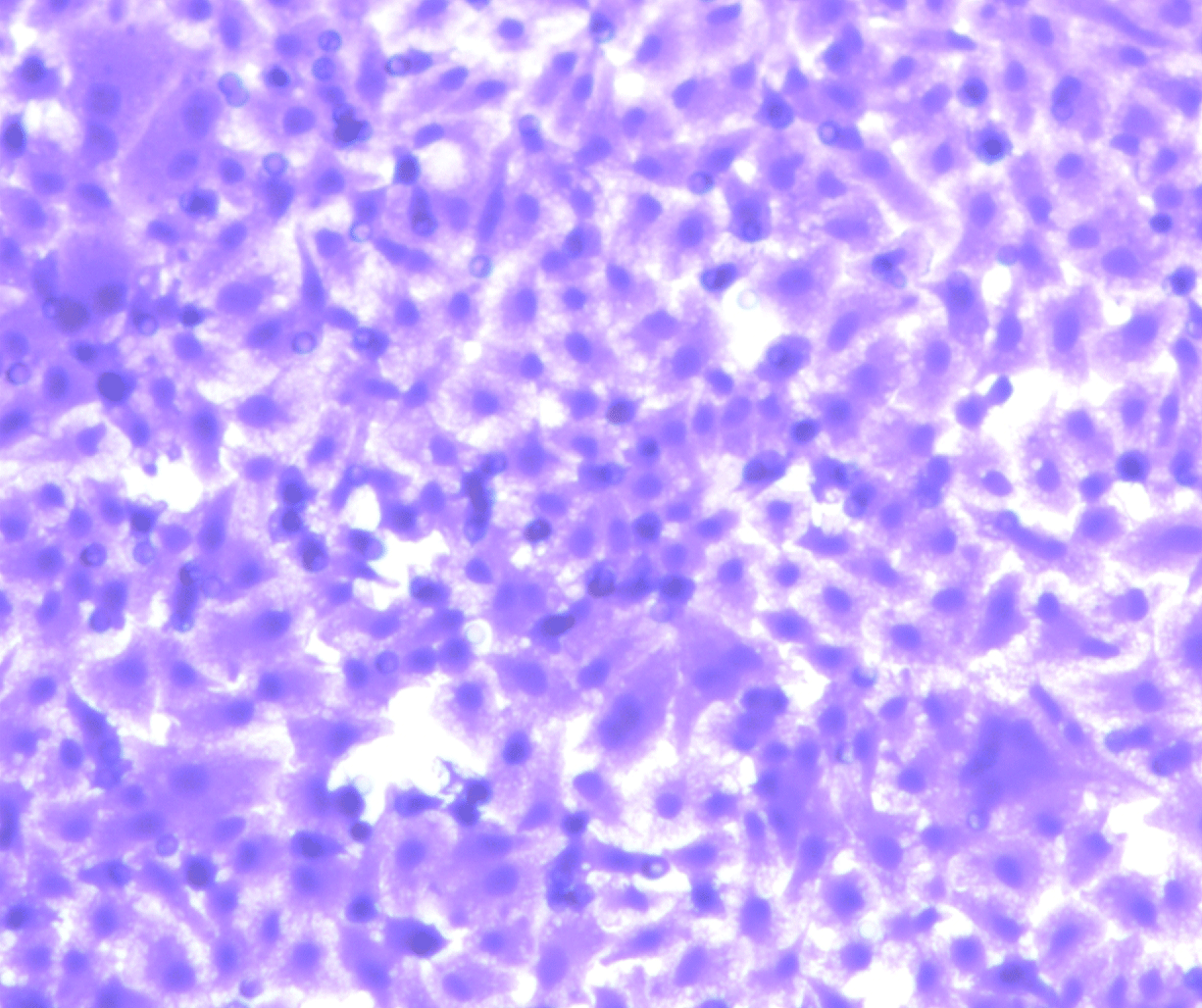

Supplement: Supplementary file 3 [file DataSheet_2.zip › Fig 3/Figure 3D and 3E transwell/invasion/Figure 3E MKN45/circPTK2 shRNA2.gif]

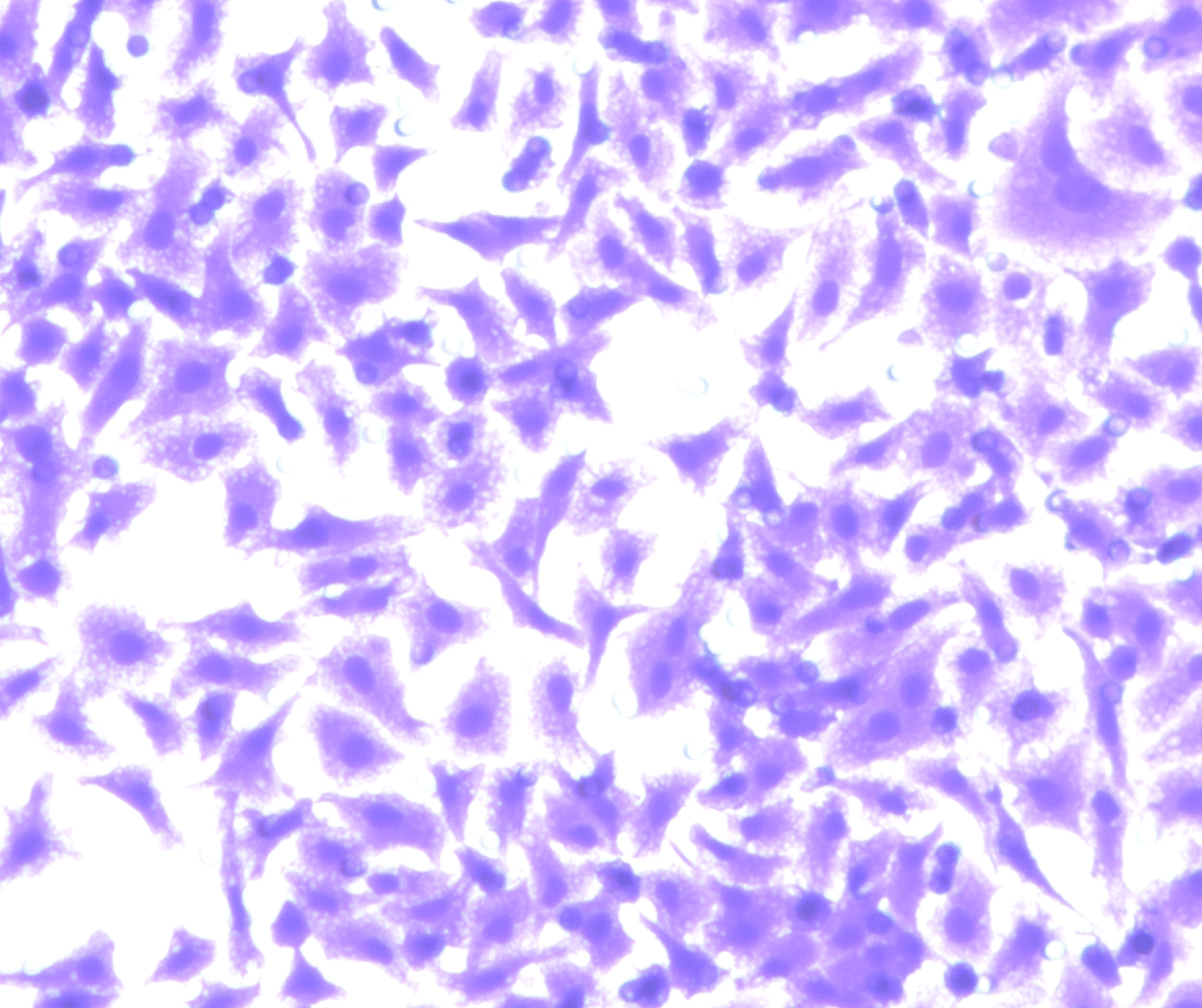

Supplement: Supplementary file 3 [file DataSheet_2.zip › Fig 3/Figure 3D and 3E transwell/invasion/Figure 3E MKN45/shRNA NC.gif]

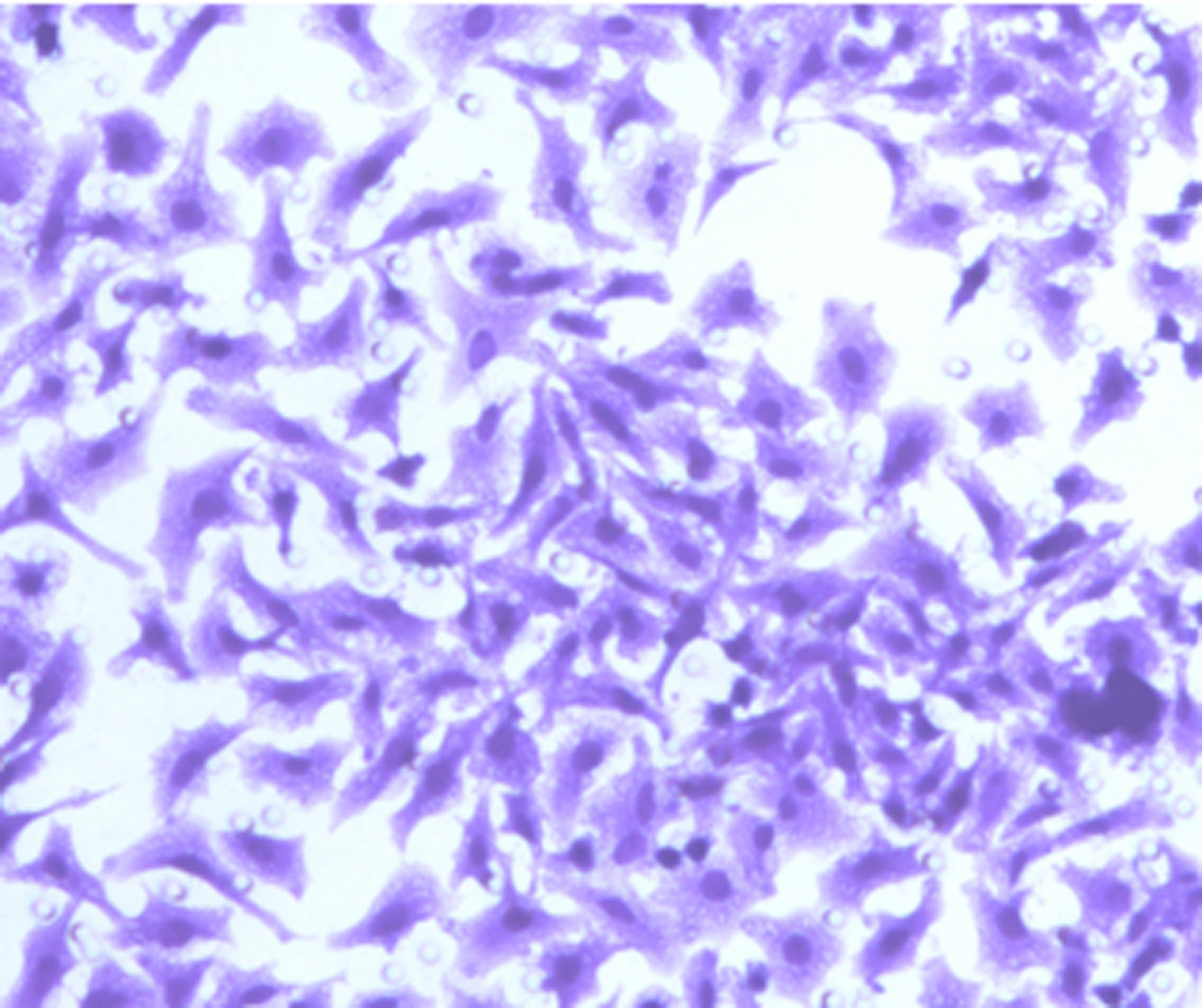

Supplement: Supplementary file 3 [file DataSheet_2.zip › Fig 3/Figure 3D and 3E transwell/migration/Figure 3D AGS/OE NC.jpg]

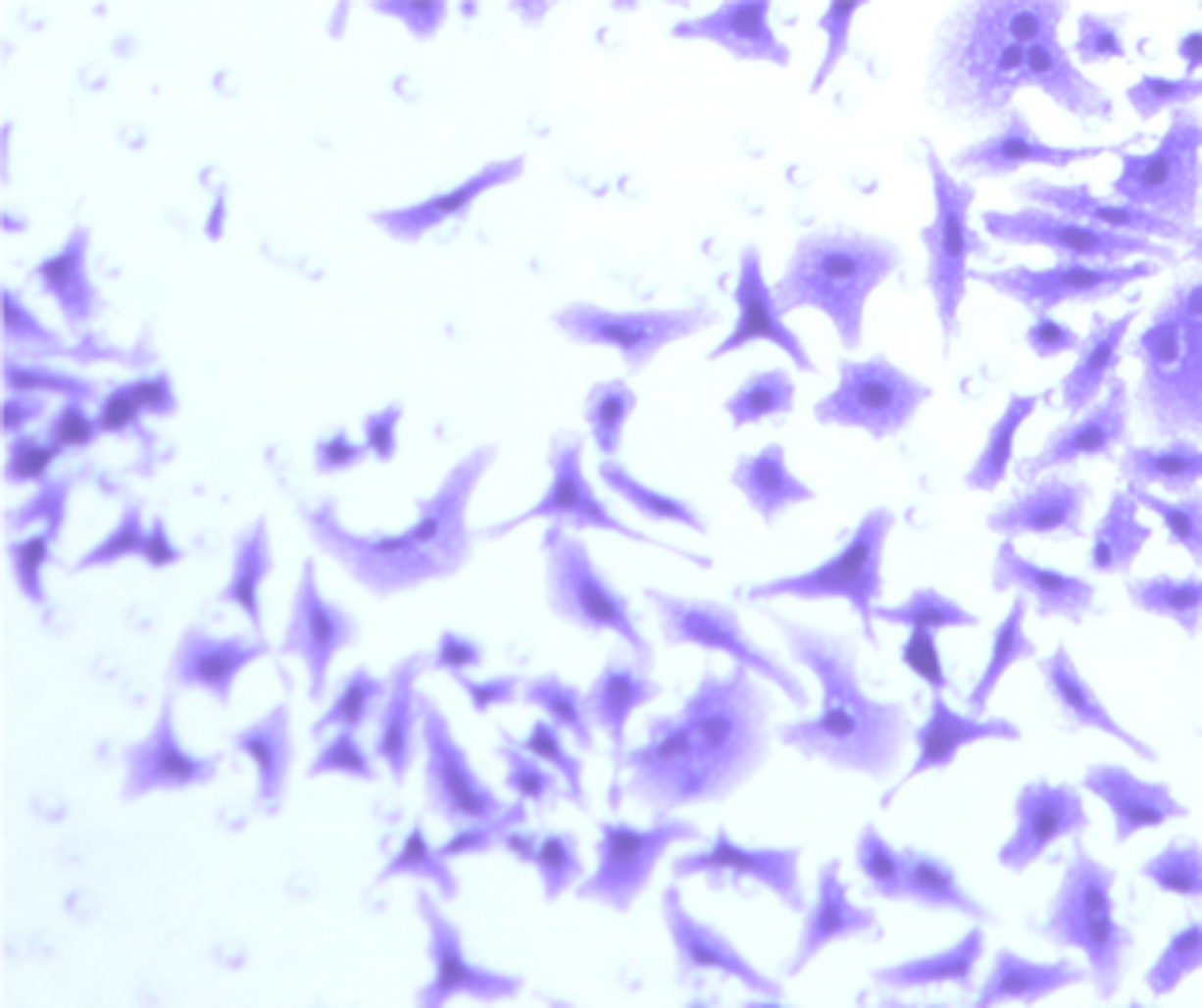

Supplement: Supplementary file 3 [file DataSheet_2.zip › Fig 3/Figure 3D and 3E transwell/migration/Figure 3D AGS/circPTK2 OE.jpg]

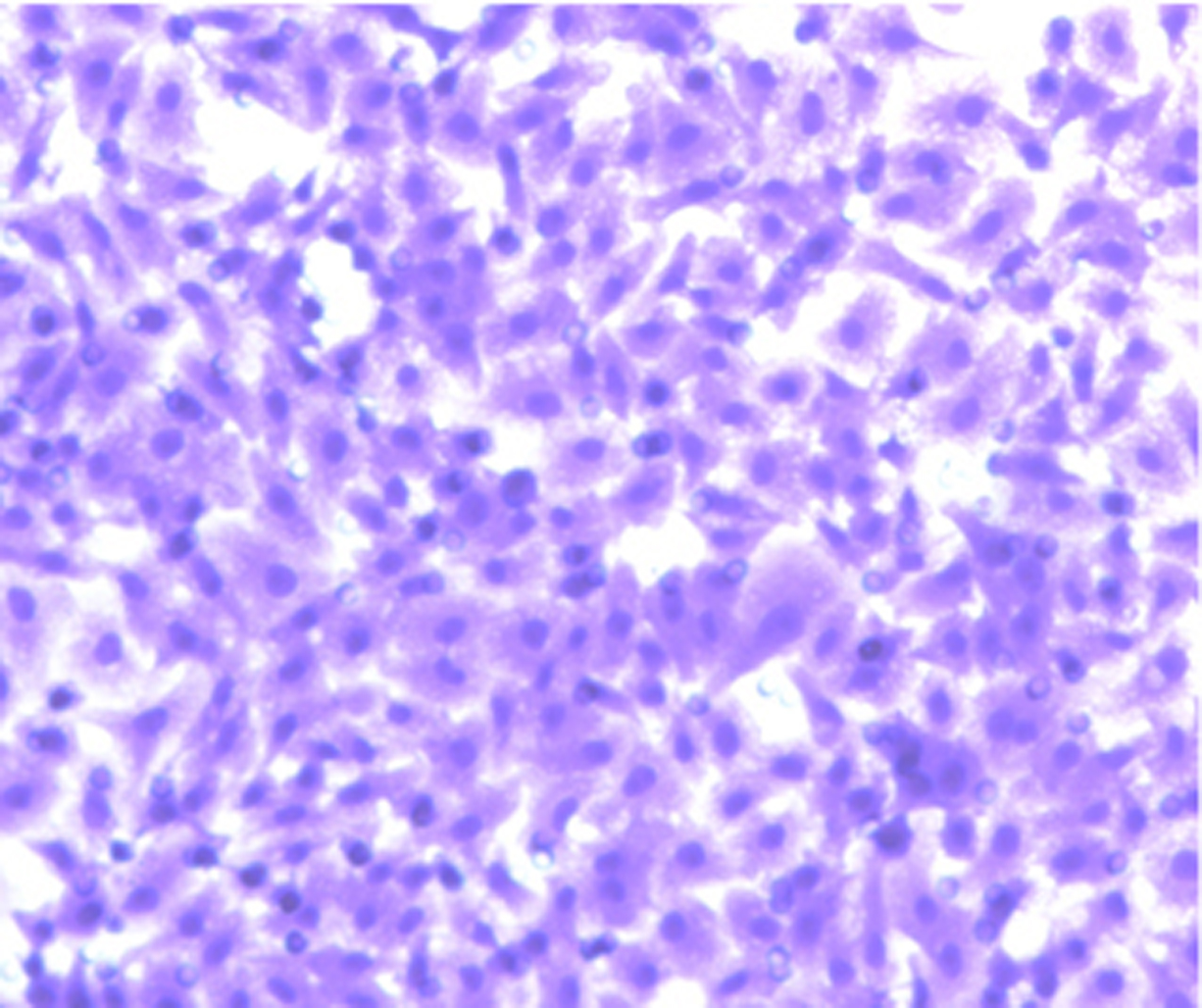

Supplement: Supplementary file 3 [file DataSheet_2.zip › Fig 3/Figure 3D and 3E transwell/migration/Figure 3D AGS/circPTK2 shRNA2.jpg]

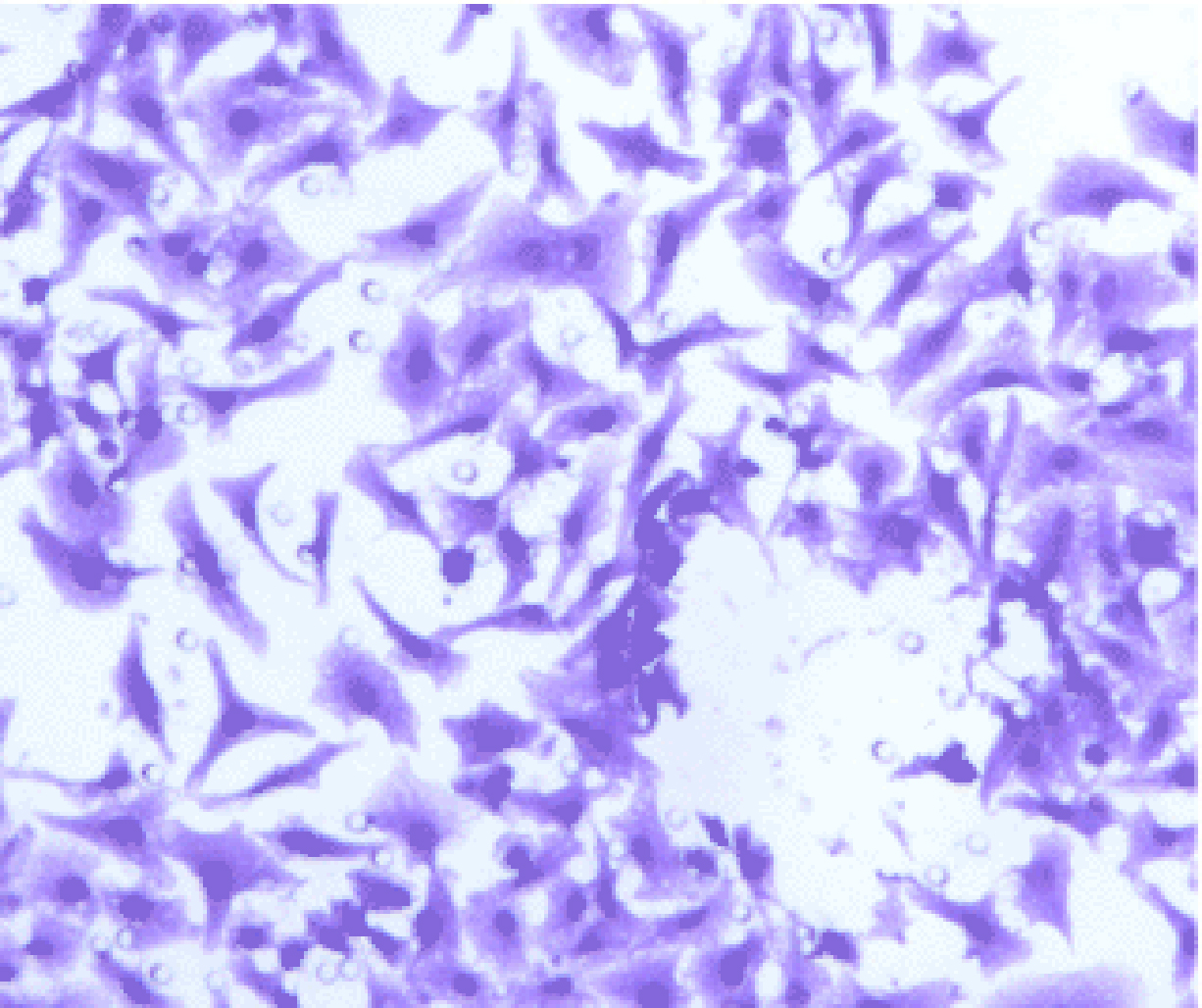

Supplement: Supplementary file 3 [file DataSheet_2.zip › Fig 3/Figure 3D and 3E transwell/migration/Figure 3D AGS/shRNA NC.jpg]

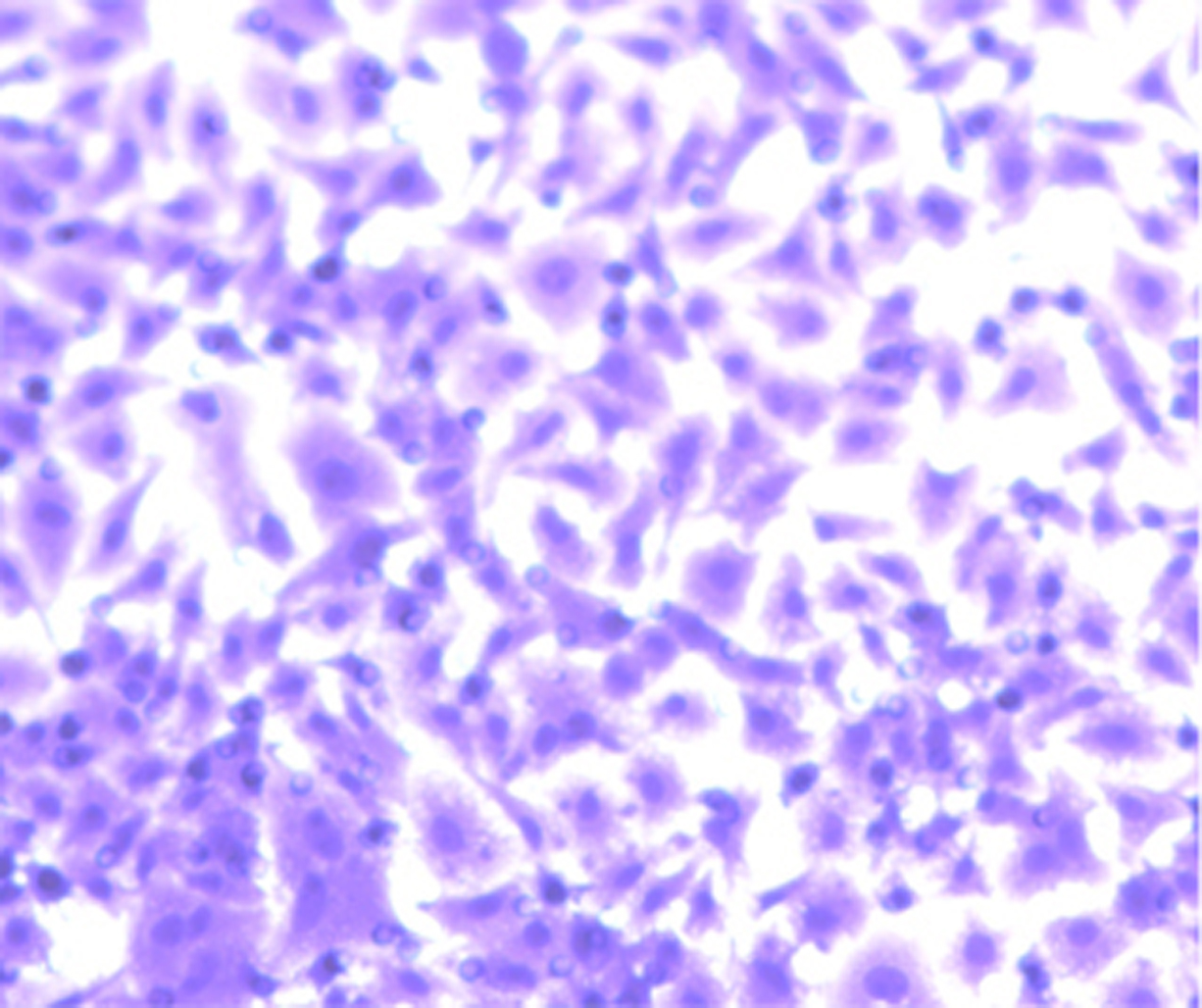

Supplement: Supplementary file 3 [file DataSheet_2.zip › Fig 3/Figure 3D and 3E transwell/migration/Figure 3D MKN45/OE NC.jpg]

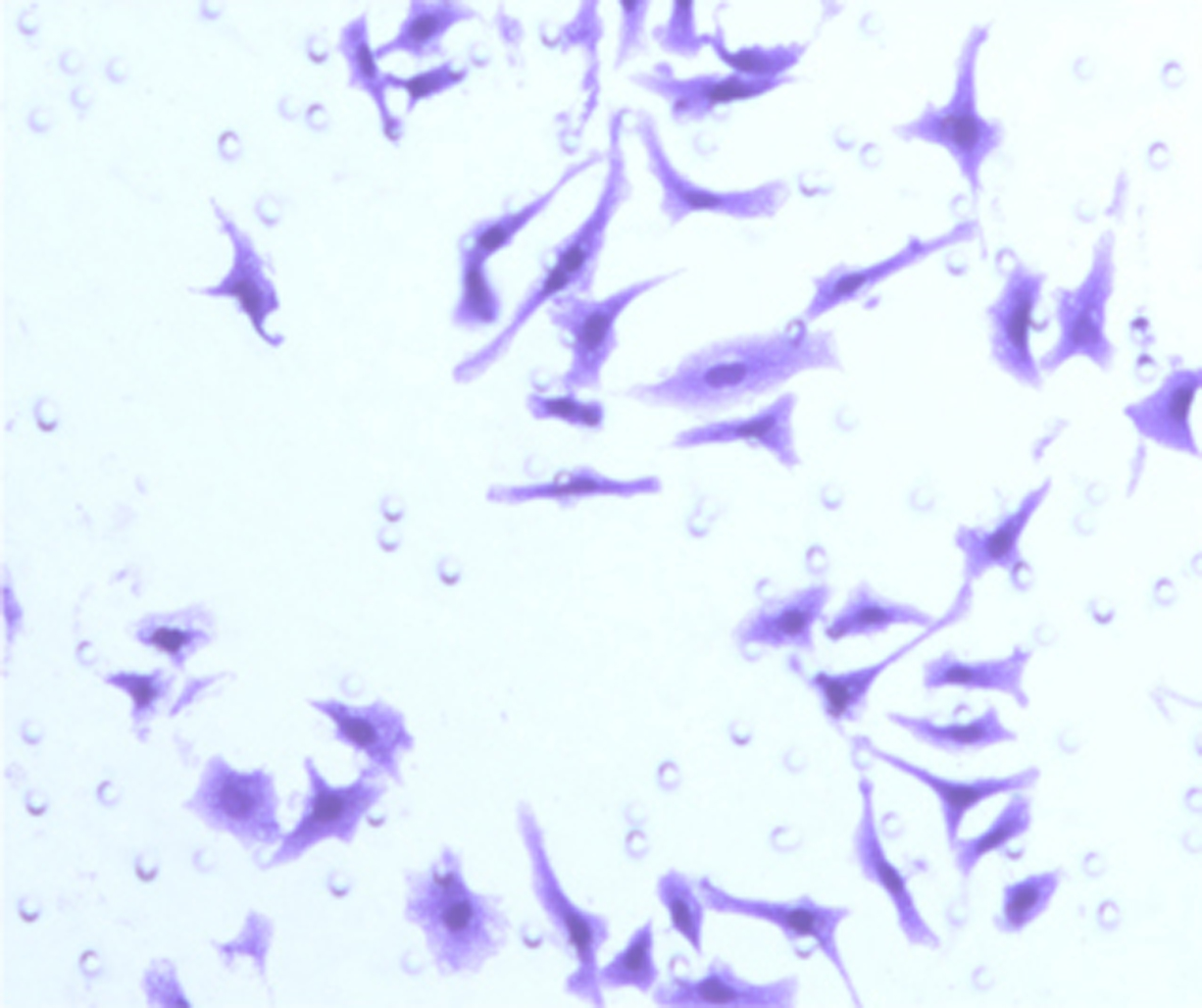

Supplement: Supplementary file 3 [file DataSheet_2.zip › Fig 3/Figure 3D and 3E transwell/migration/Figure 3D MKN45/circPTK2 OE.jpg]

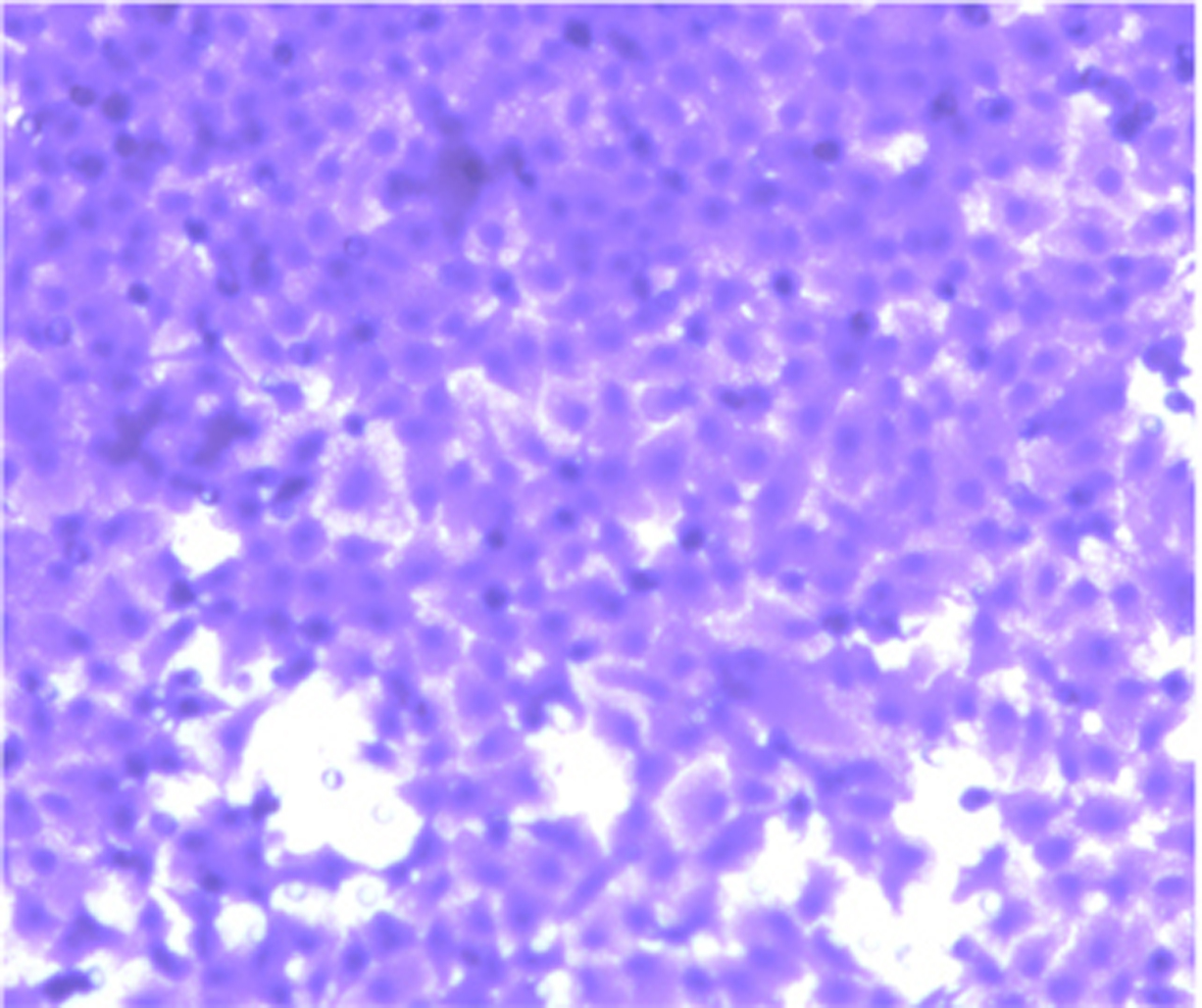

Supplement: Supplementary file 3 [file DataSheet_2.zip › Fig 3/Figure 3D and 3E transwell/migration/Figure 3D MKN45/circPTK2 shRNA2.jpg]

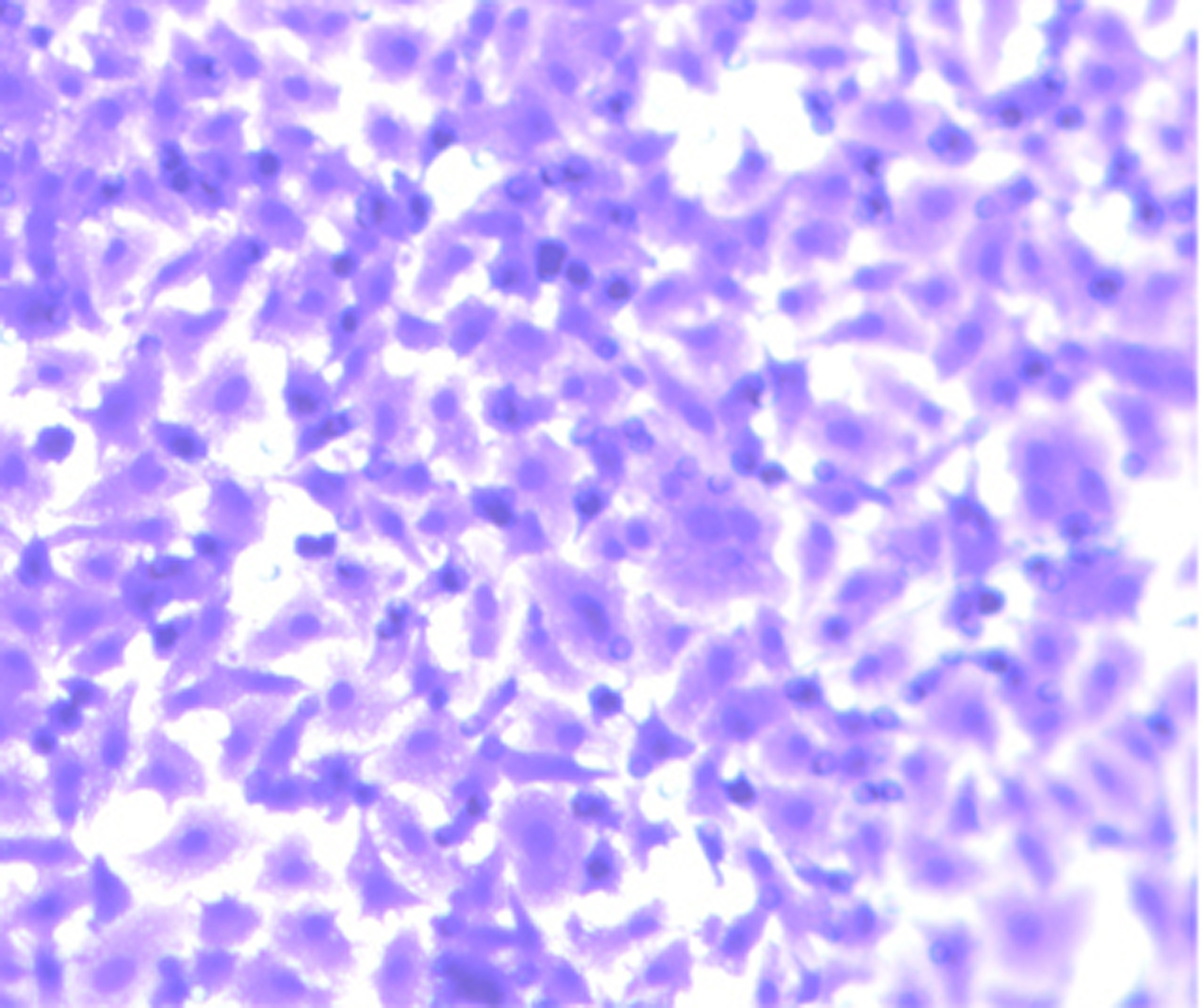

Supplement: Supplementary file 3 [file DataSheet_2.zip › Fig 3/Figure 3D and 3E transwell/migration/Figure 3D MKN45/shRNA NC.jpg]

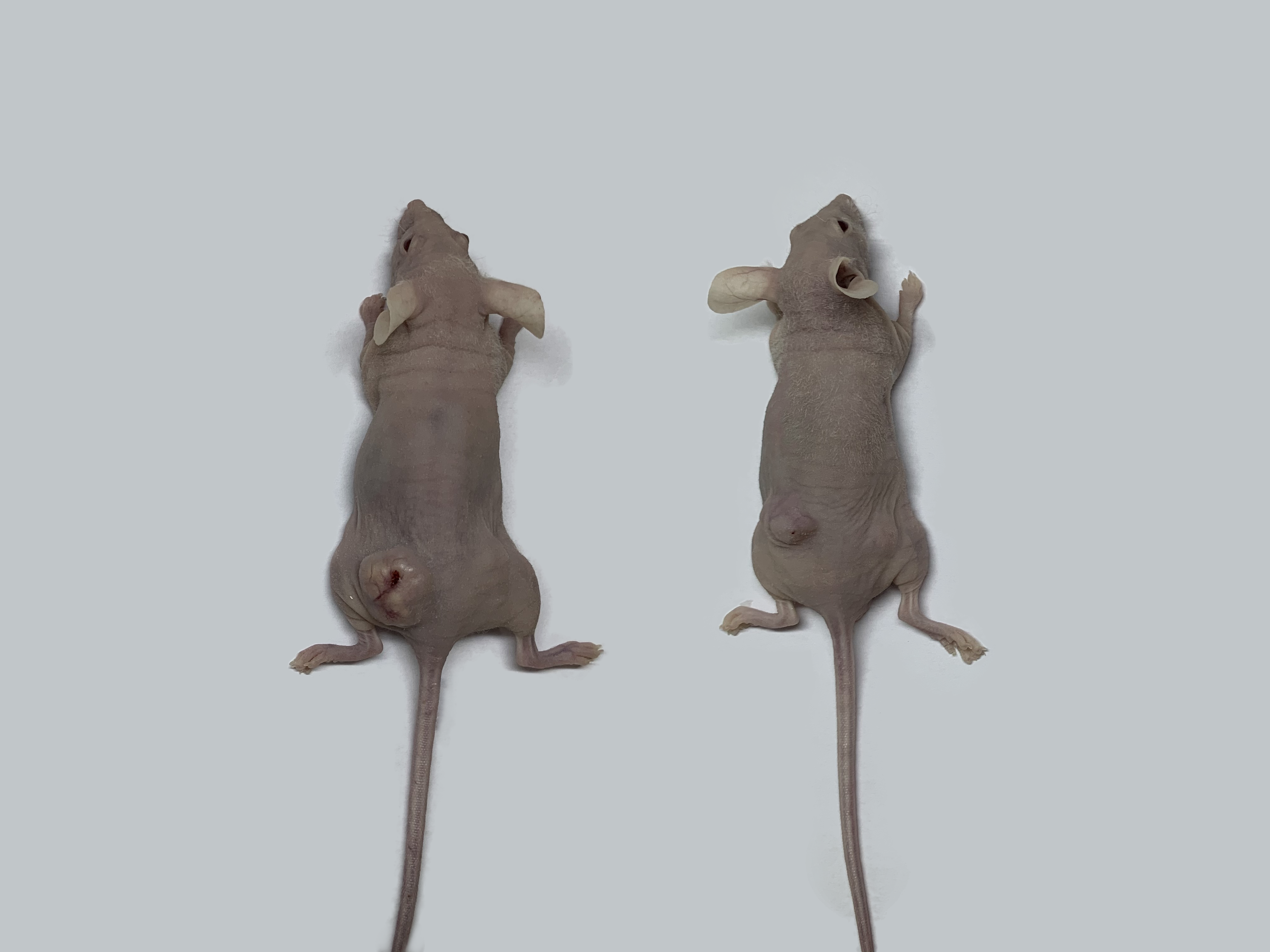

Supplement: Supplementary file 4 [file DataSheet_3.zip › Fig 4/AGS.tif]

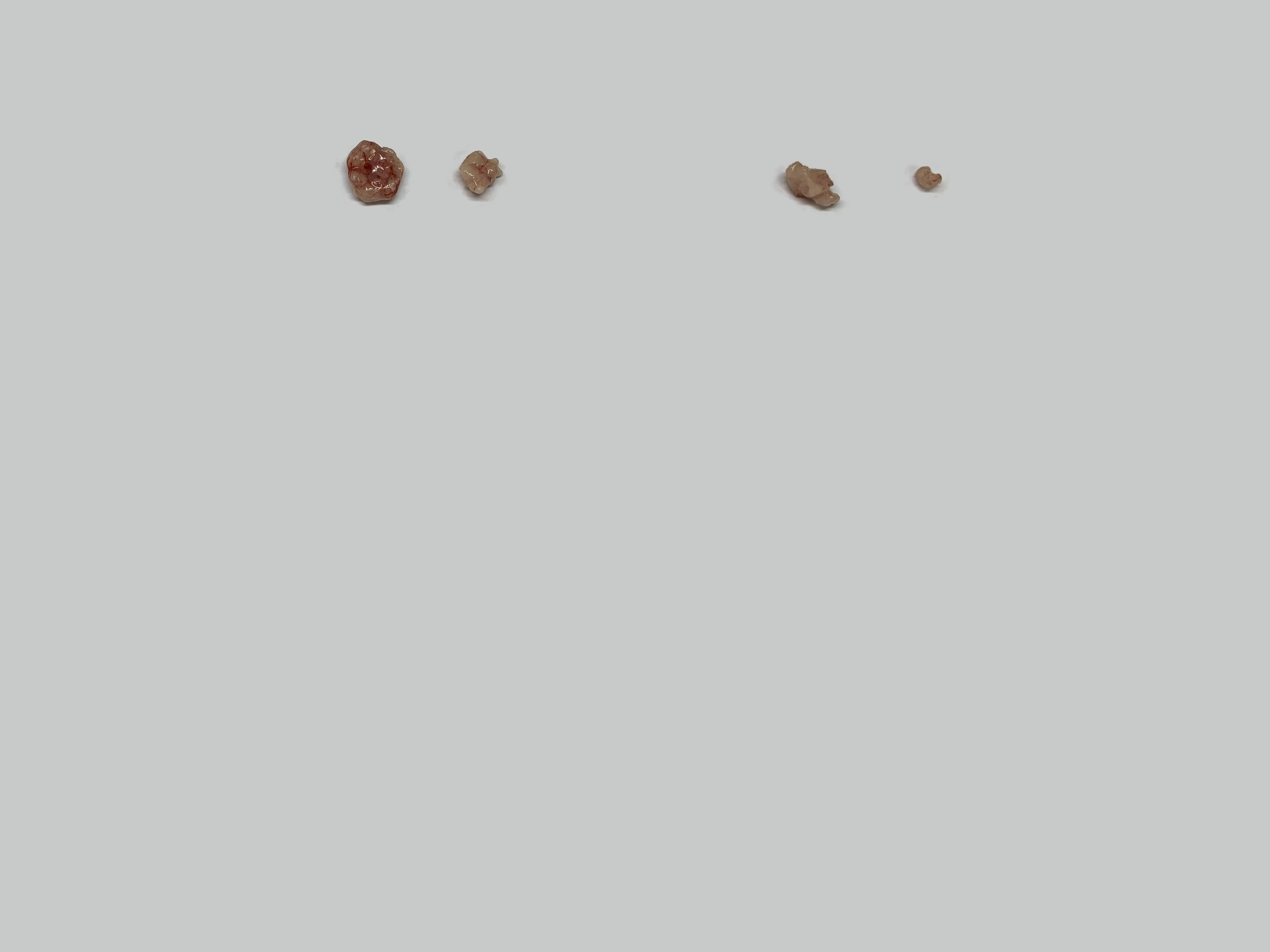

Supplement: Supplementary file 4 [file DataSheet_3.zip › Fig 4/Figure 4A AGS.tif]

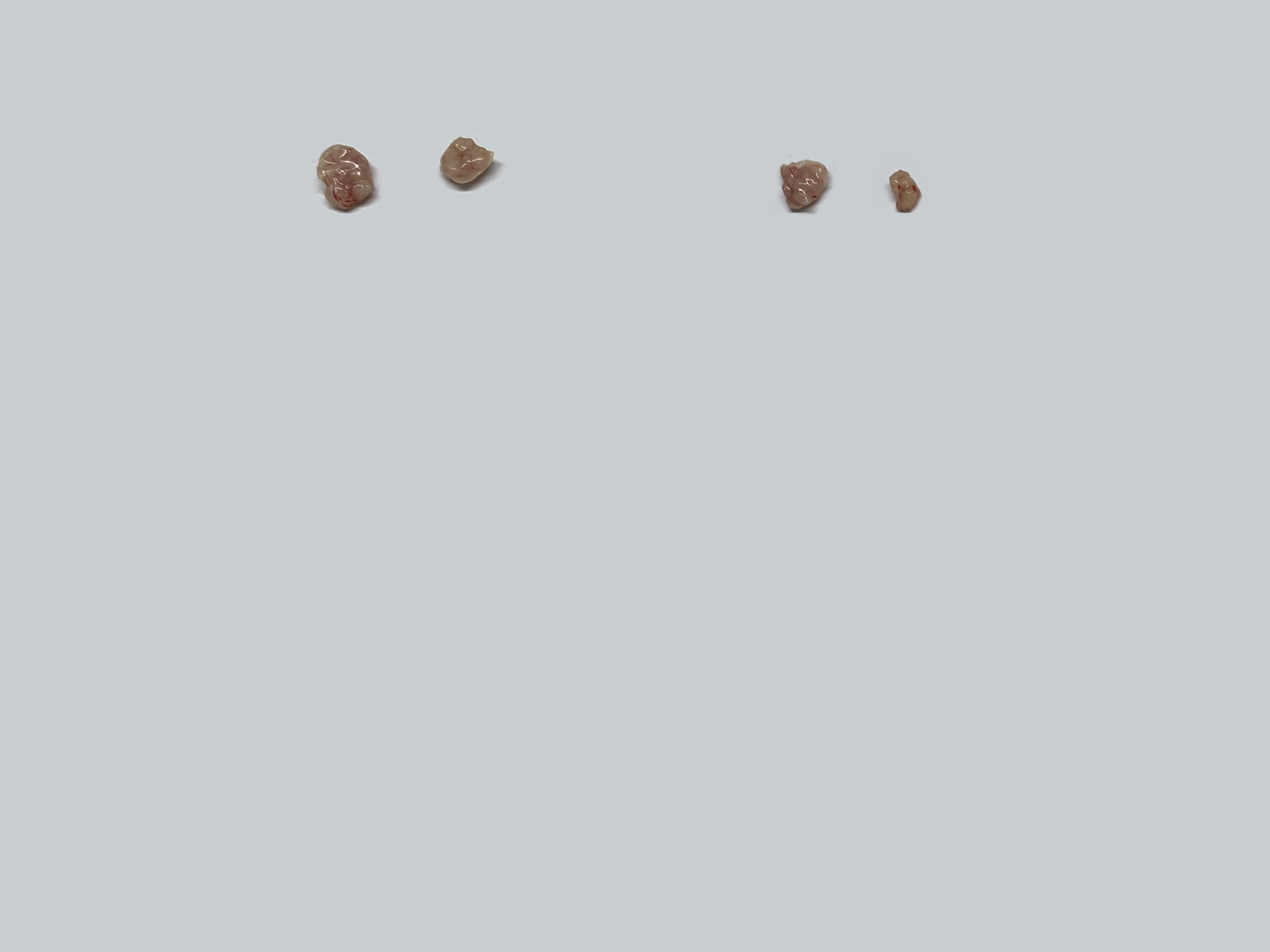

Supplement: Supplementary file 4 [file DataSheet_3.zip › Fig 4/Figure 4A MKN45.tif]

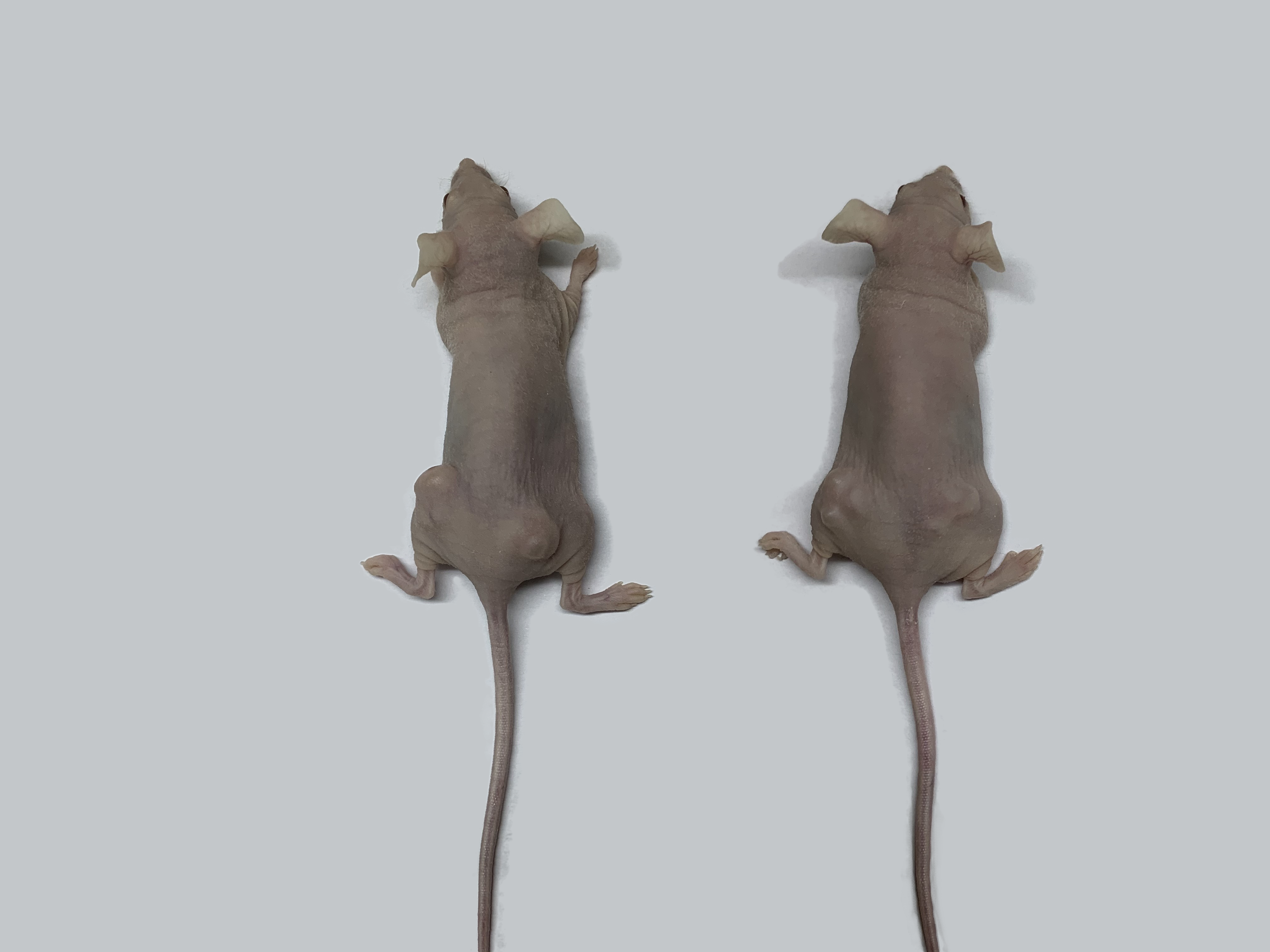

Supplement: Supplementary file 4 [file DataSheet_3.zip › Fig 4/MKN45.tif]

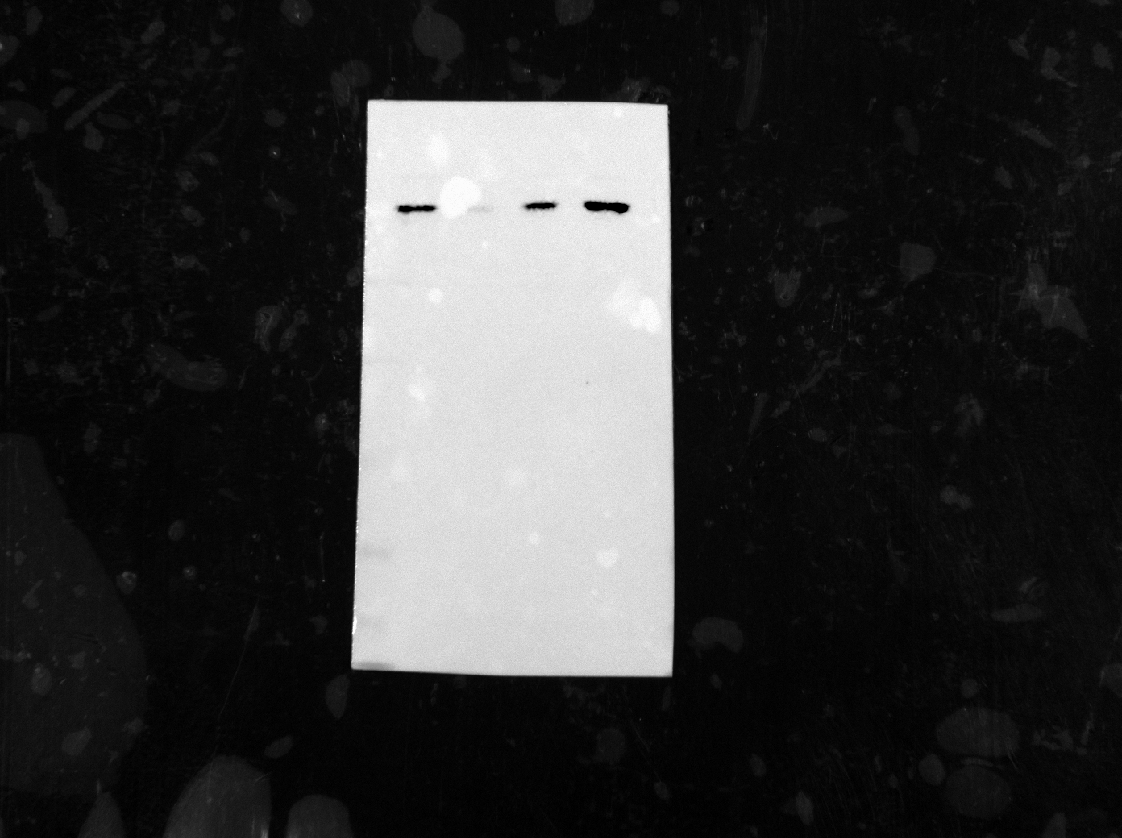

Supplement: Supplementary file 5 [file DataSheet_4.zip › Fig 6/Figure 6D-WB/AAKT-AGS.tif]

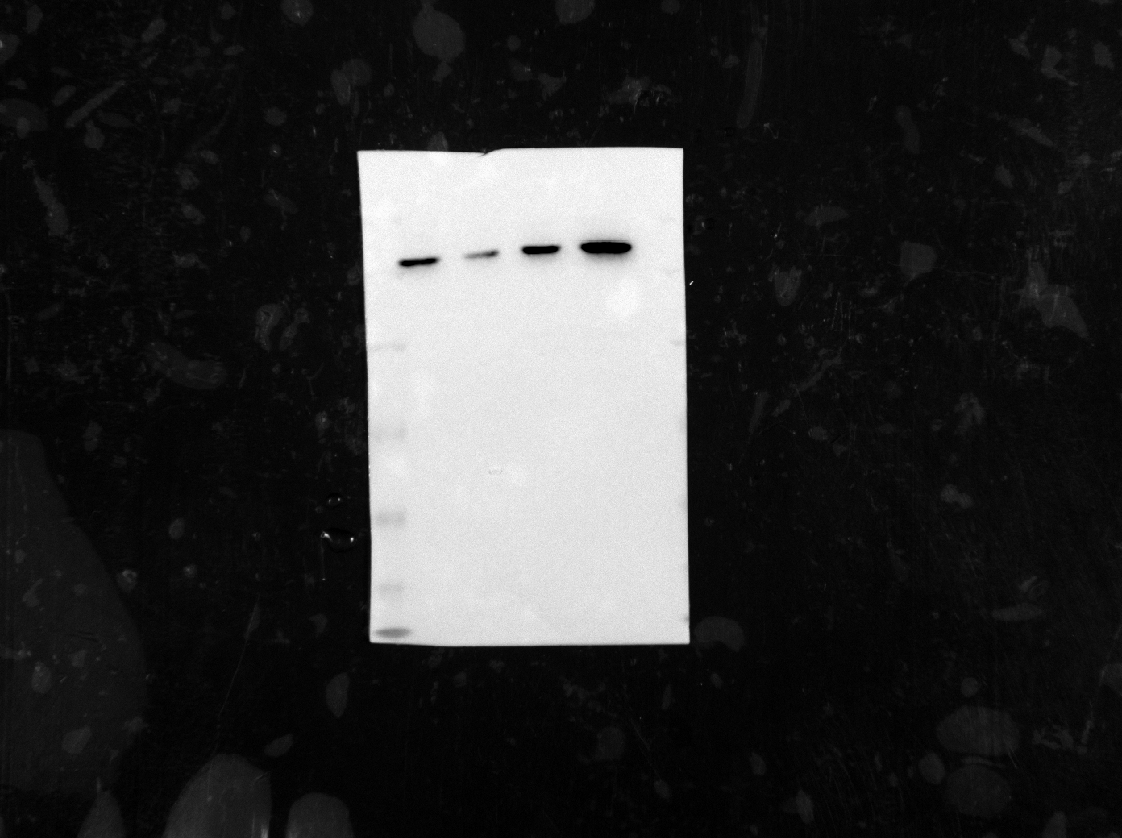

Supplement: Supplementary file 5 [file DataSheet_4.zip › Fig 6/Figure 6D-WB/AAKT-MKN45.tif]

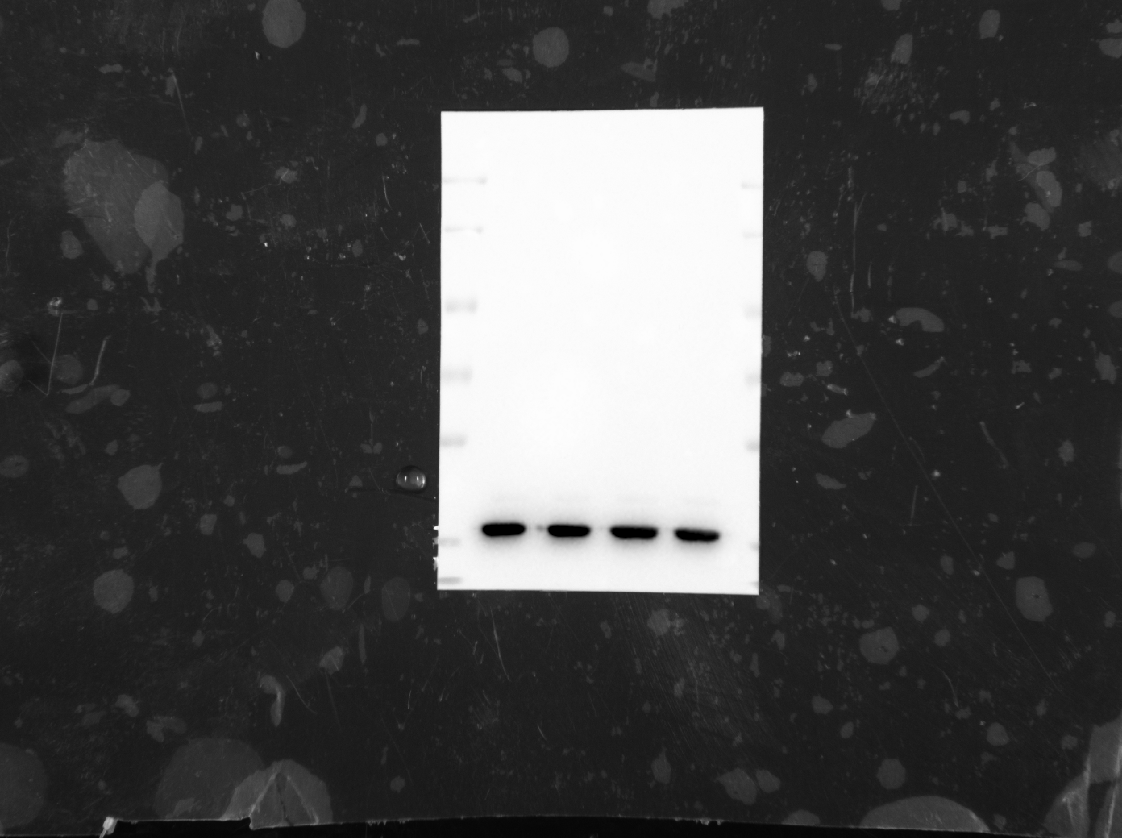

Supplement: Supplementary file 5 [file DataSheet_4.zip › Fig 6/Figure 6D-WB/GAPDH-AGS.tif]

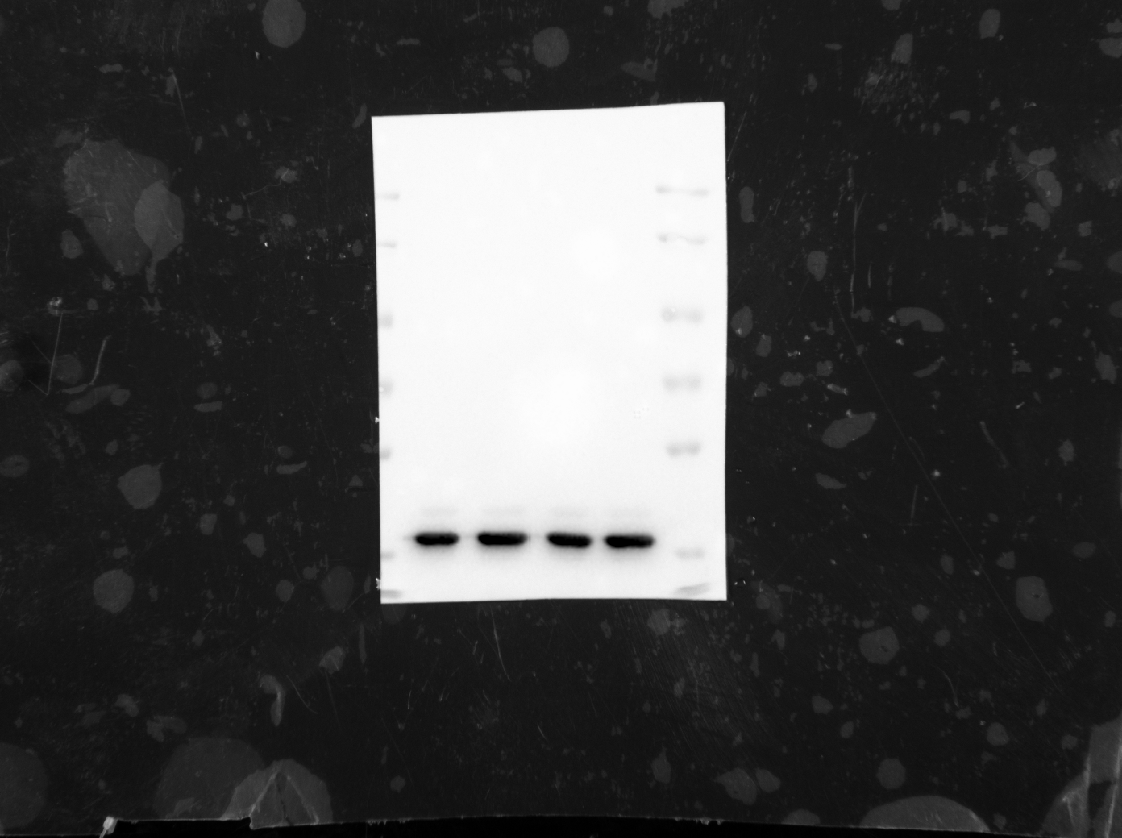

Supplement: Supplementary file 5 [file DataSheet_4.zip › Fig 6/Figure 6D-WB/GAPDH-MKN45.tif]

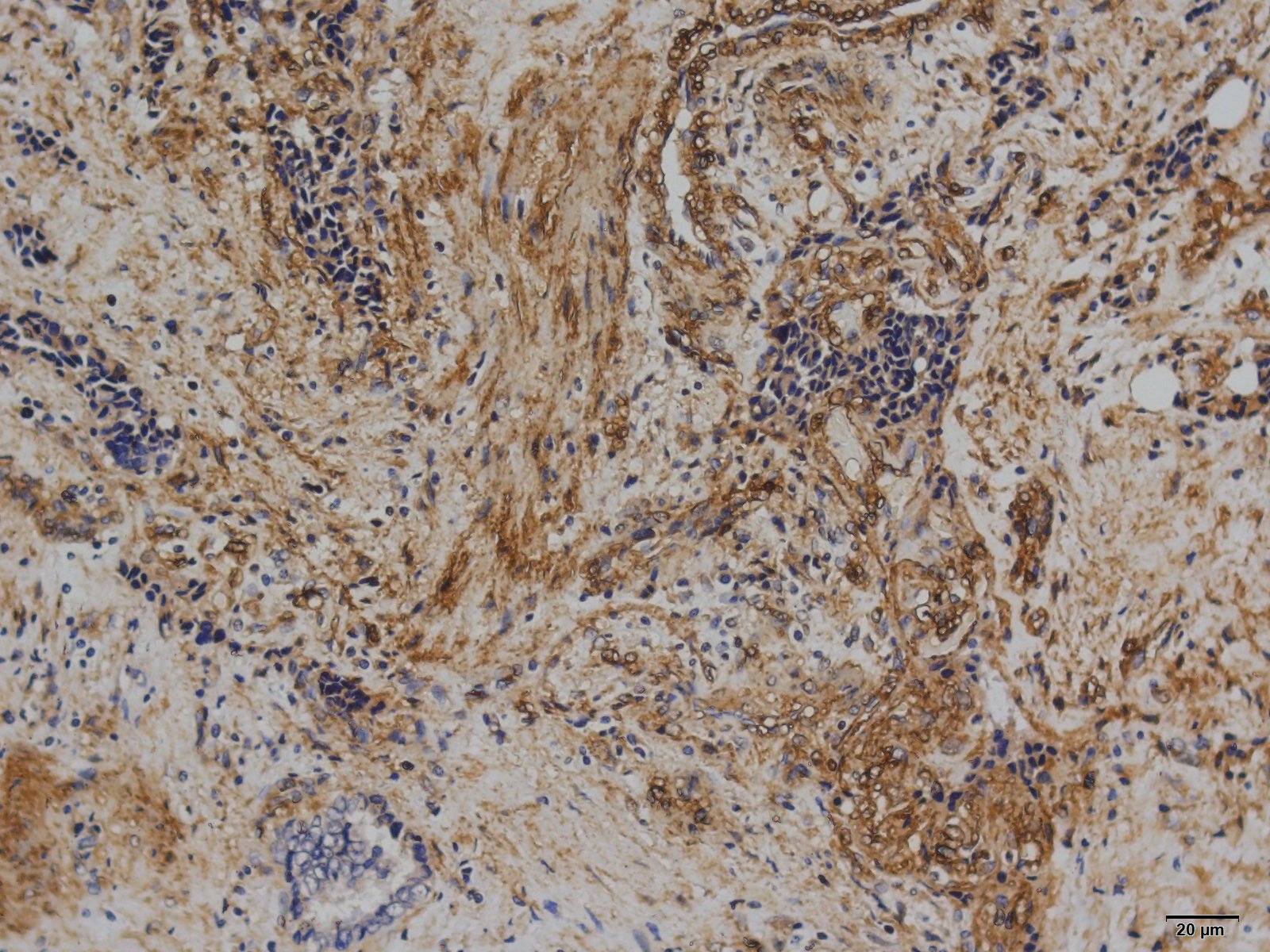

Supplement: Supplementary file 5 [file DataSheet_4.zip › Fig 6/Figure 6J IHC/circPTK2 normal.jpg]

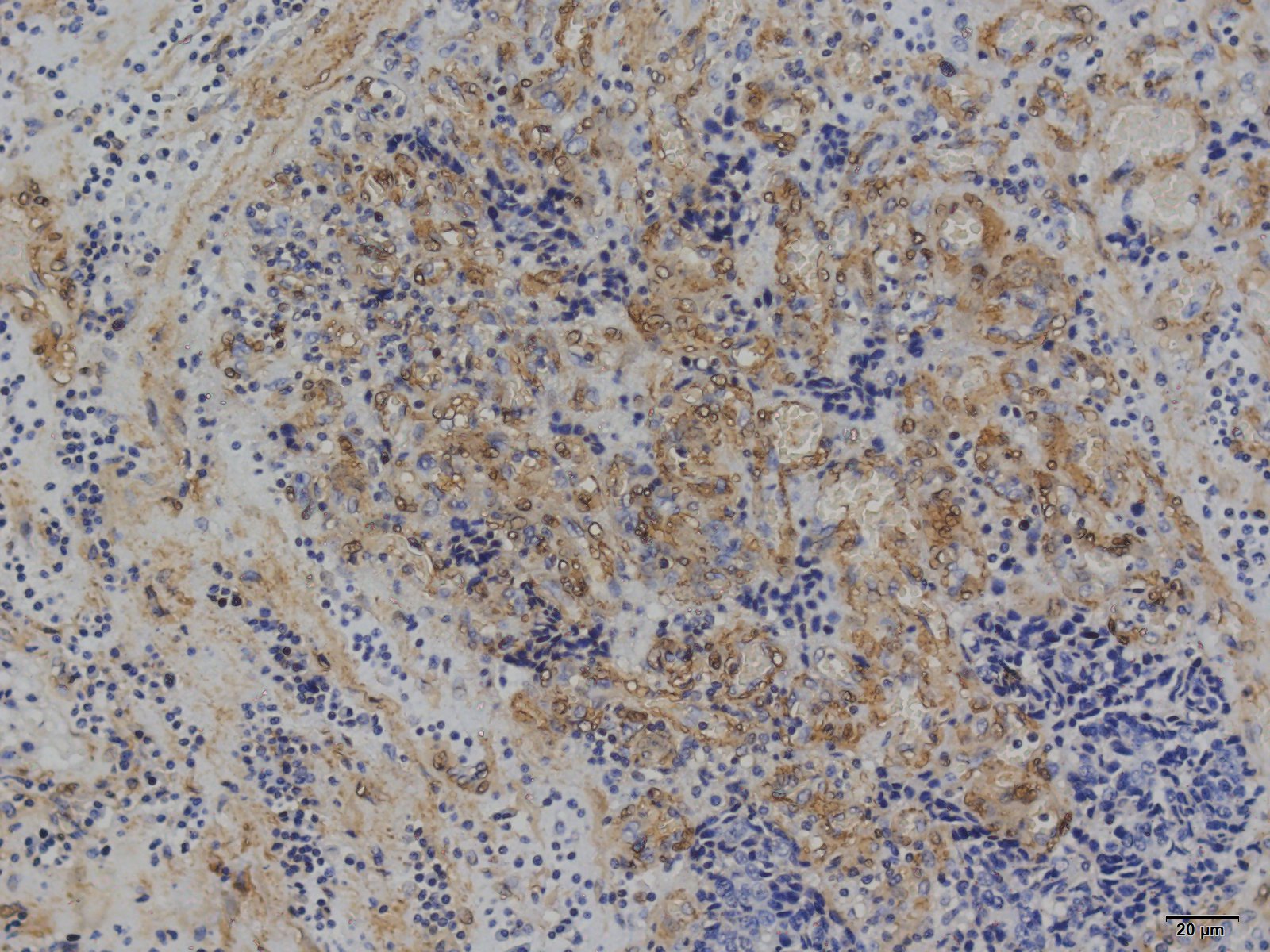

Supplement: Supplementary file 5 [file DataSheet_4.zip › Fig 6/Figure 6J IHC/circPTK2 tumor.jpg]

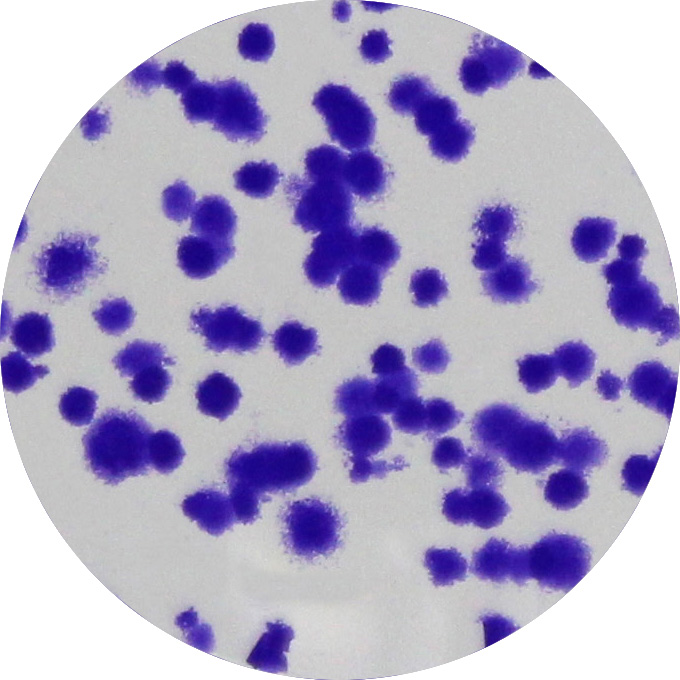

Supplement: Supplementary file 6 [file DataSheet_5.zip › Figure 7C colony formation/AGS/circPTK2+AATK shRNA.jpg]

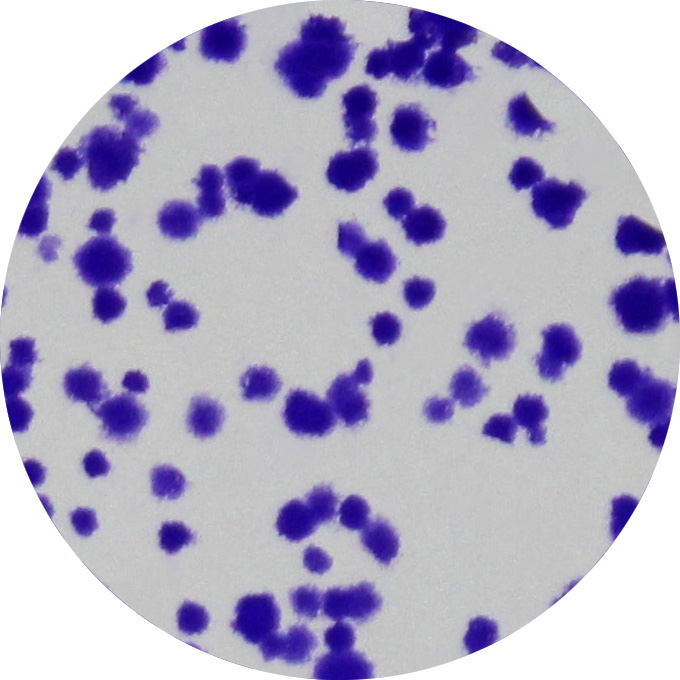

Supplement: Supplementary file 6 [file DataSheet_5.zip › Figure 7C colony formation/AGS/circPTK2+miR-196a-3p.jpg]

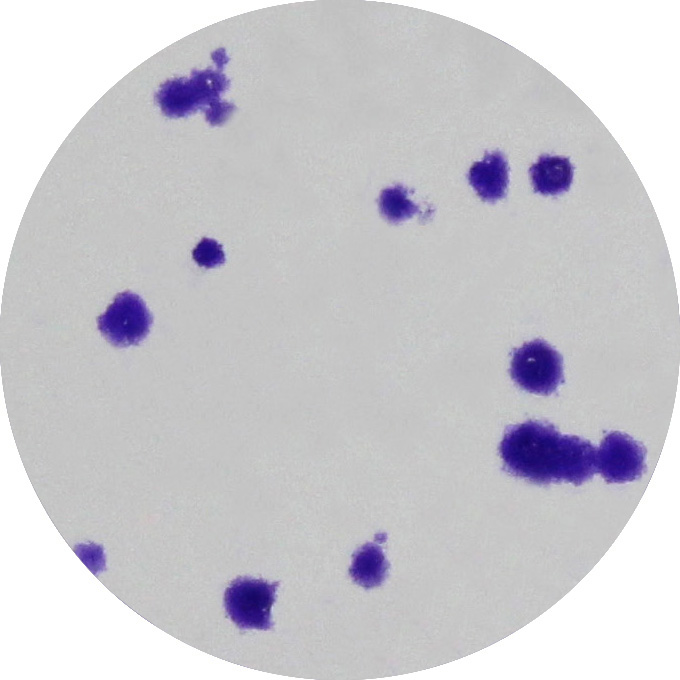

Supplement: Supplementary file 6 [file DataSheet_5.zip › Figure 7C colony formation/AGS/circPTK2+mimics NC.jpg]

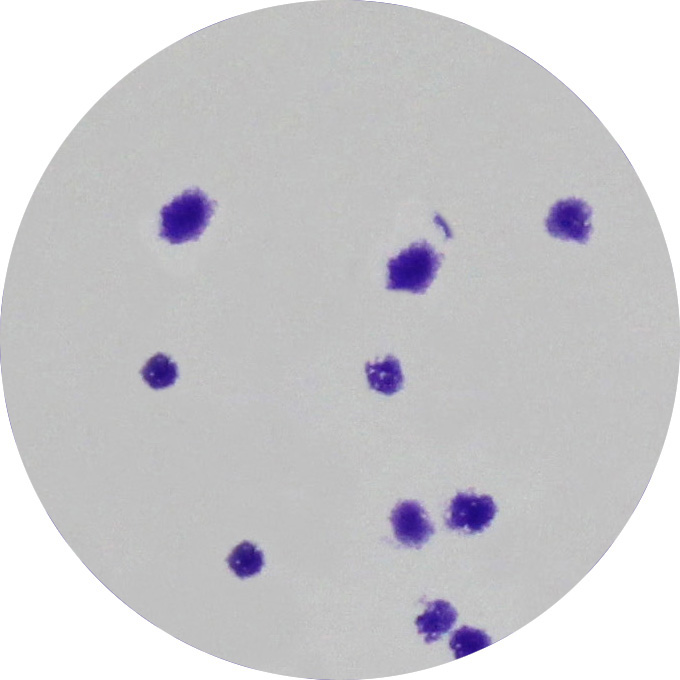

Supplement: Supplementary file 6 [file DataSheet_5.zip › Figure 7C colony formation/AGS/circPTK2+shRNA NC.jpg]

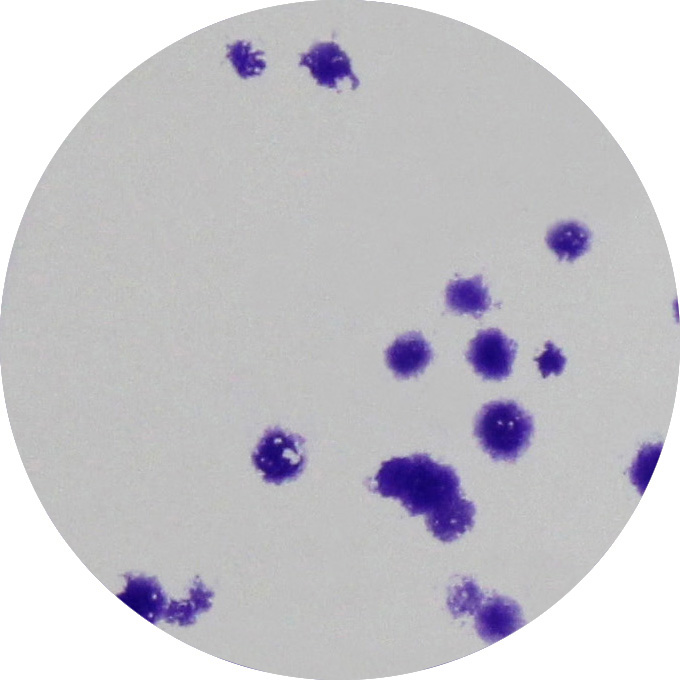

Supplement: Supplementary file 6 [file DataSheet_5.zip › Figure 7C colony formation/AGS/circPTK2.jpg]

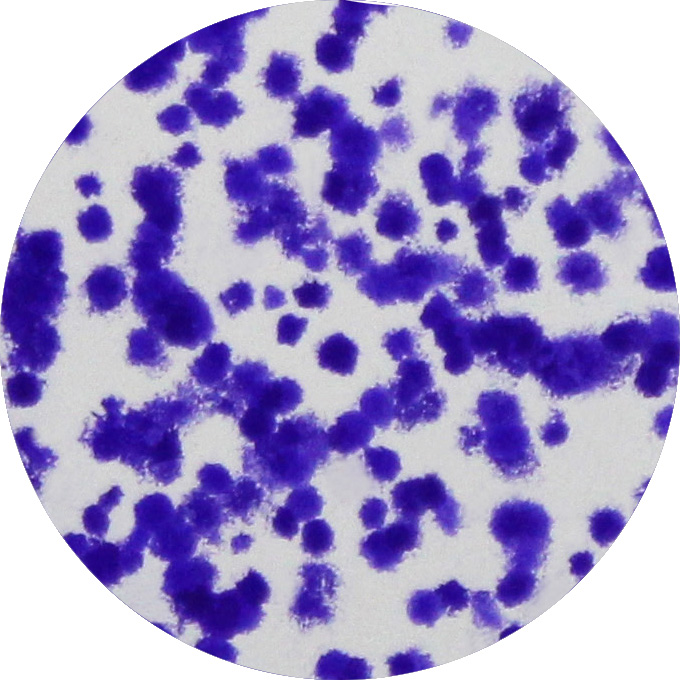

Supplement: Supplementary file 6 [file DataSheet_5.zip › Figure 7C colony formation/AGS/control.jpg]

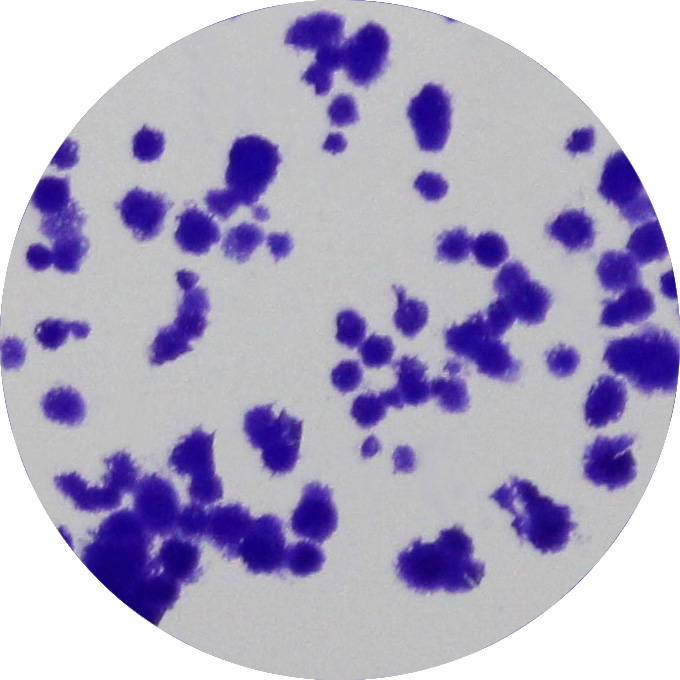

Supplement: Supplementary file 6 [file DataSheet_5.zip › Figure 7C colony formation/MKN45/circPTK2+AATK shRNA.jpg]

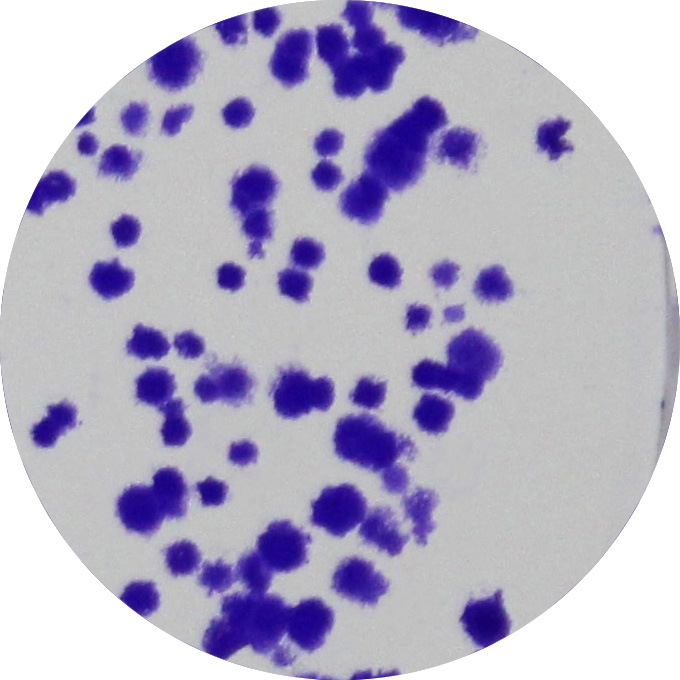

Supplement: Supplementary file 6 [file DataSheet_5.zip › Figure 7C colony formation/MKN45/circPTK2+miR-196a-3p.jpg]

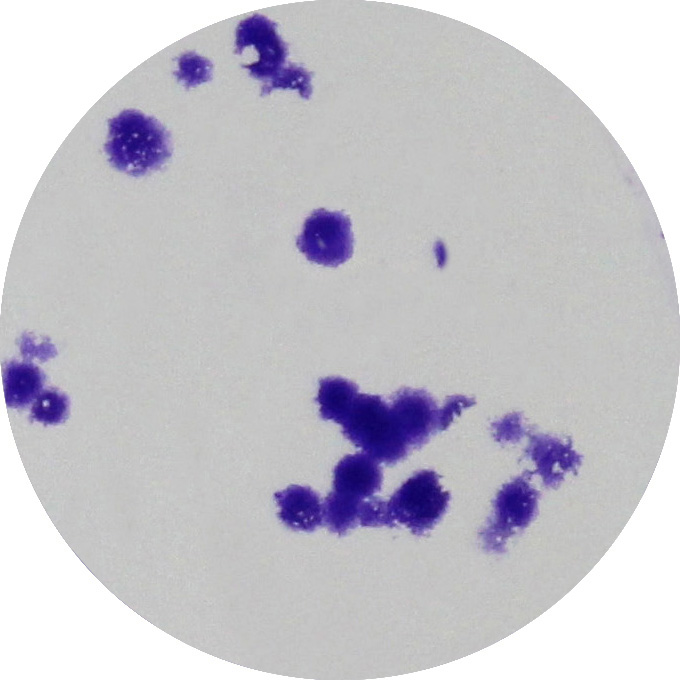

Supplement: Supplementary file 6 [file DataSheet_5.zip › Figure 7C colony formation/MKN45/circPTK2+mimics NC.jpg]

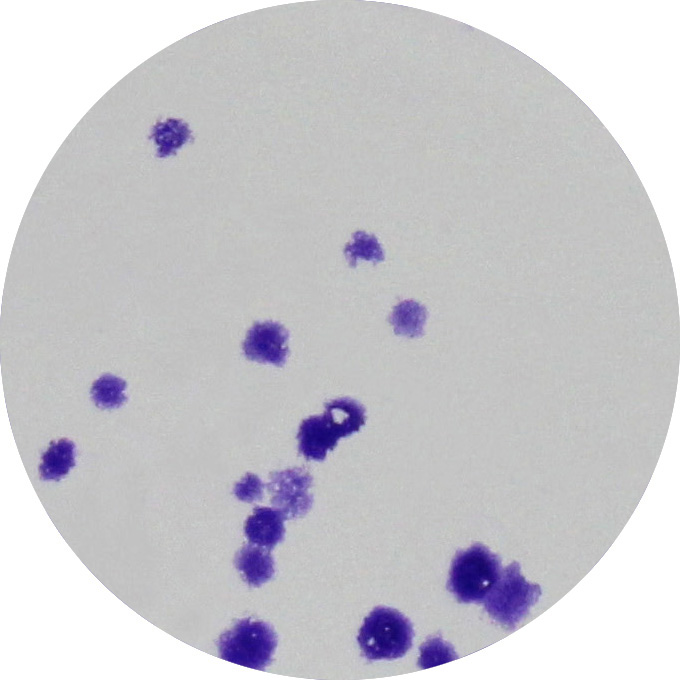

Supplement: Supplementary file 6 [file DataSheet_5.zip › Figure 7C colony formation/MKN45/circPTK2+shRNA NC.jpg]

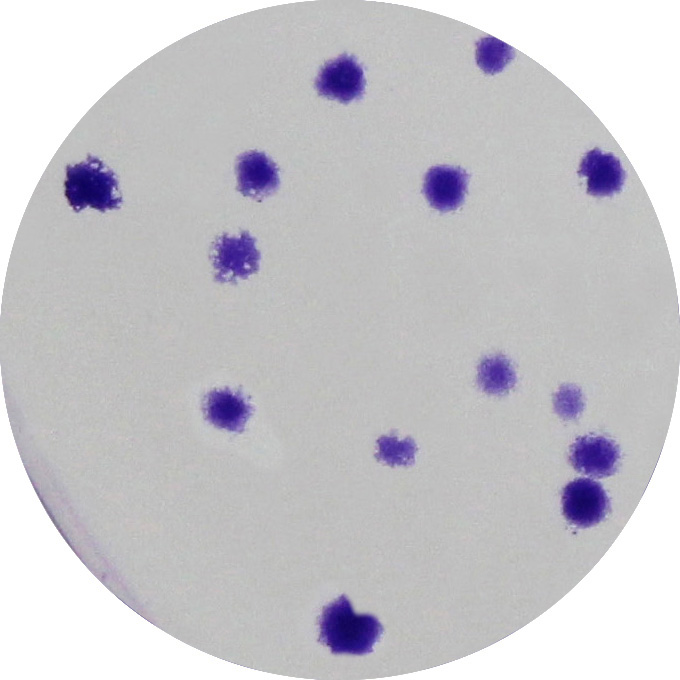

Supplement: Supplementary file 6 [file DataSheet_5.zip › Figure 7C colony formation/MKN45/circPTK2.jpg]

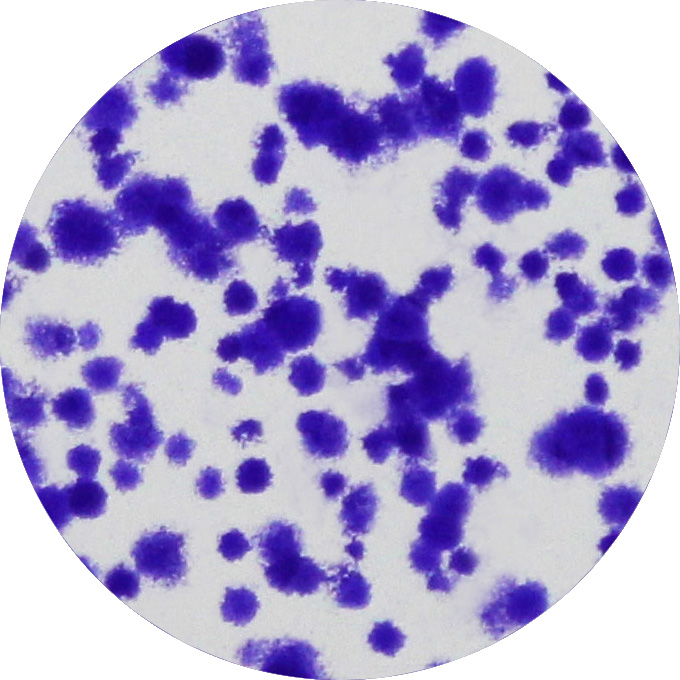

Supplement: Supplementary file 6 [file DataSheet_5.zip › Figure 7C colony formation/MKN45/control.jpg]

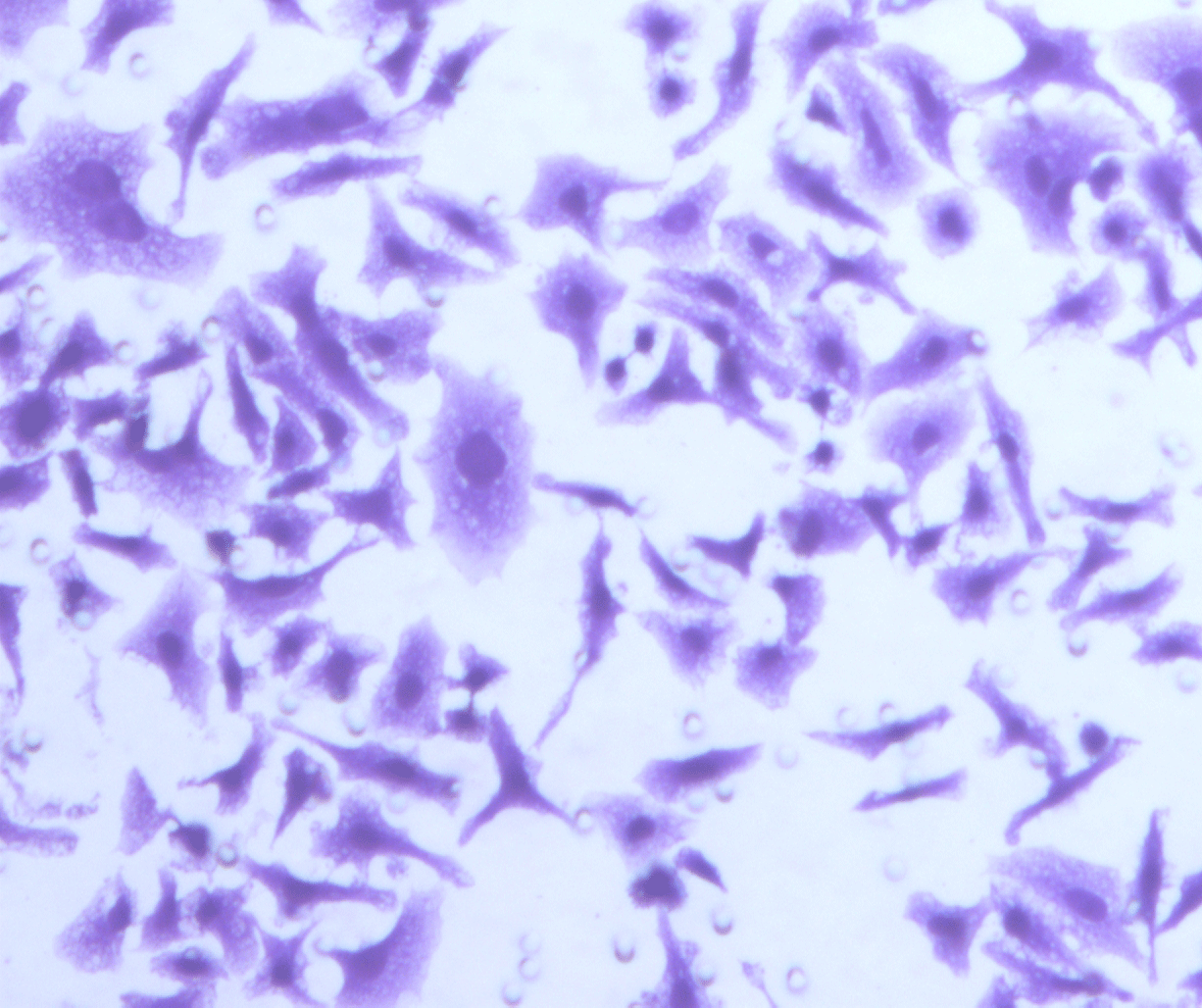

Supplement: Supplementary file 7 [file DataSheet_6.zip › Figure 7D, 7E transwell/Figure 7D migration/AGS/circPTK2+AATK shRNA.gif]

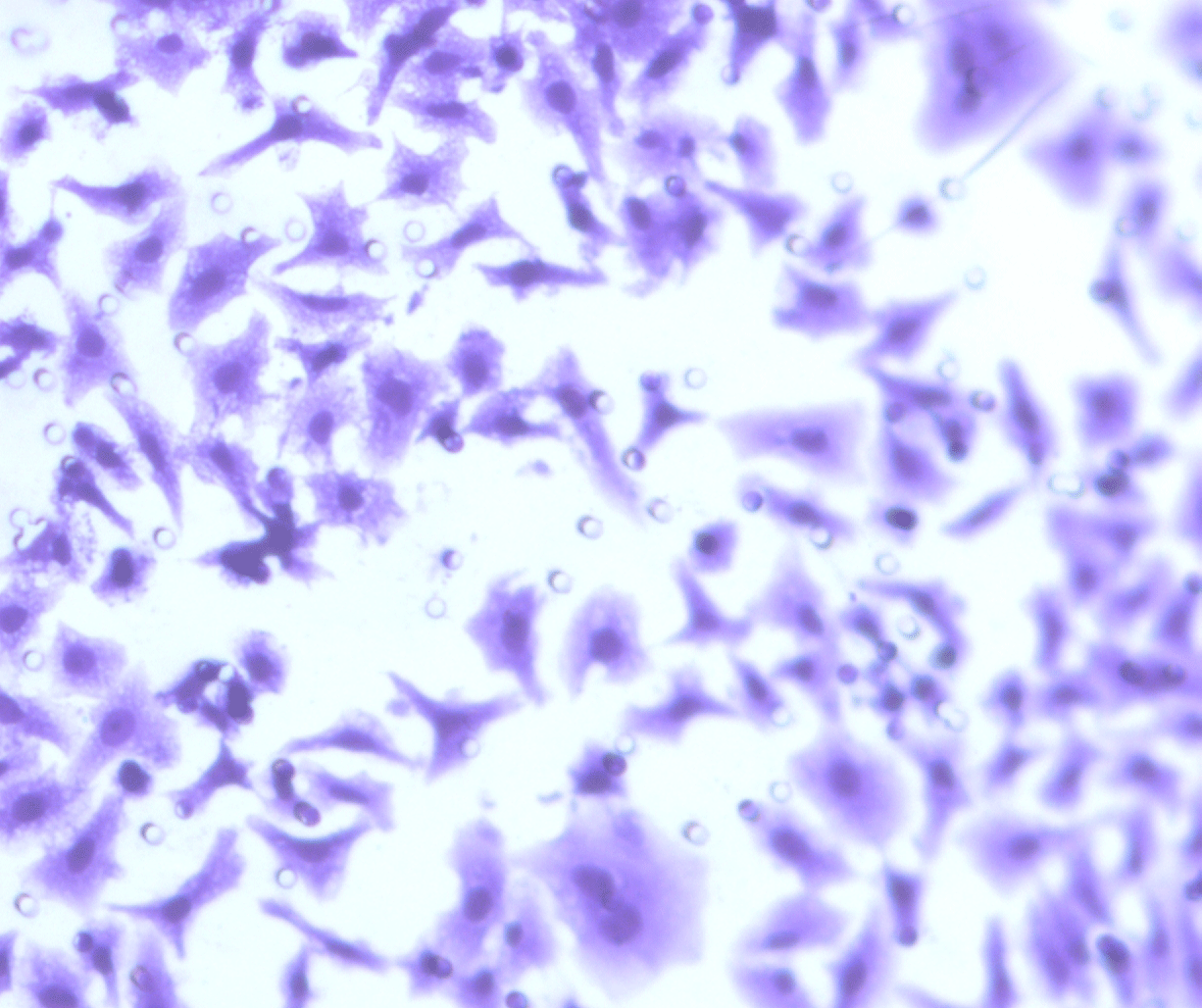

Supplement: Supplementary file 7 [file DataSheet_6.zip › Figure 7D, 7E transwell/Figure 7D migration/AGS/circPTK2+miR-196a-3p.gif]

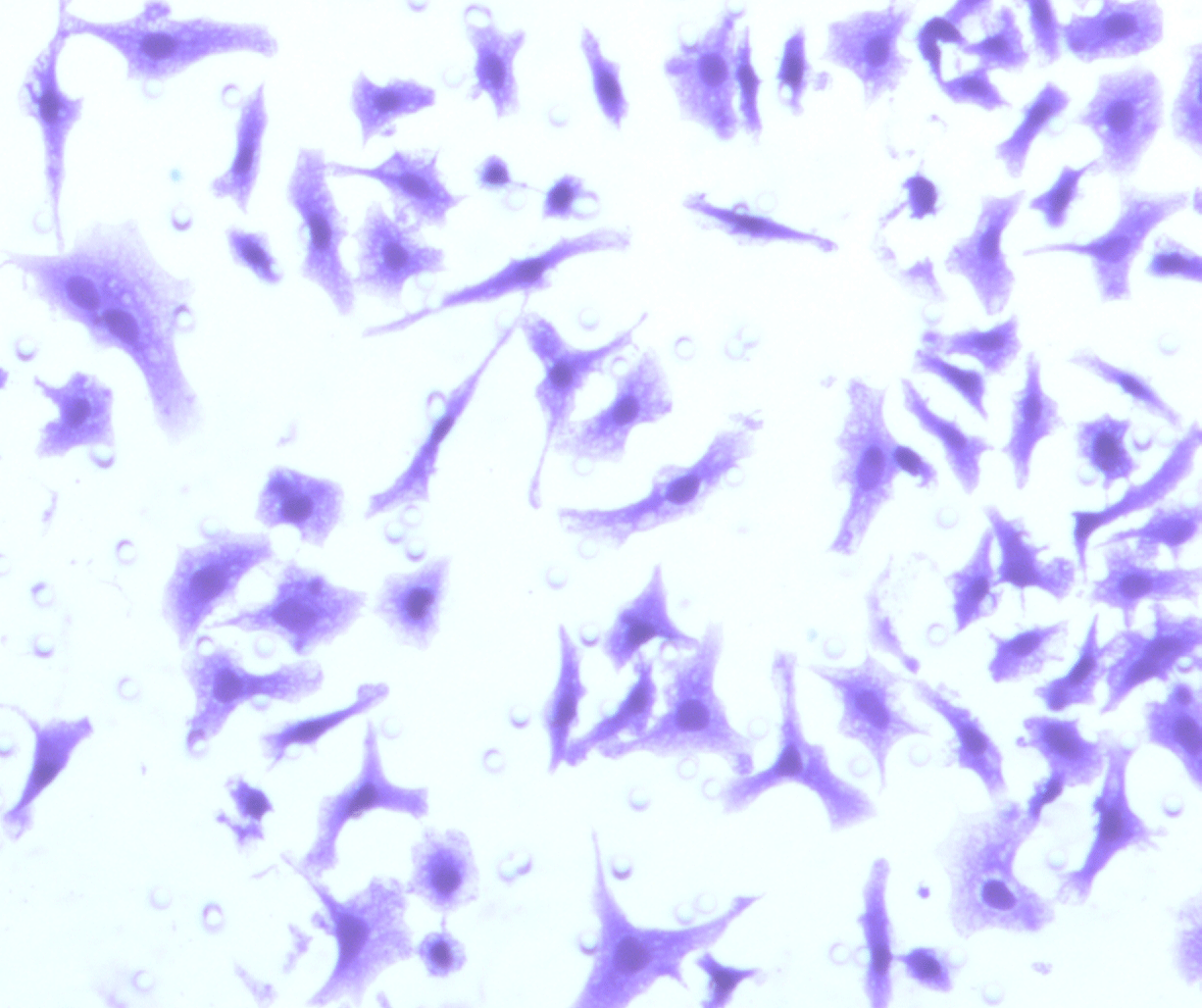

Supplement: Supplementary file 7 [file DataSheet_6.zip › Figure 7D, 7E transwell/Figure 7D migration/AGS/circPTK2+mimics NC.gif]

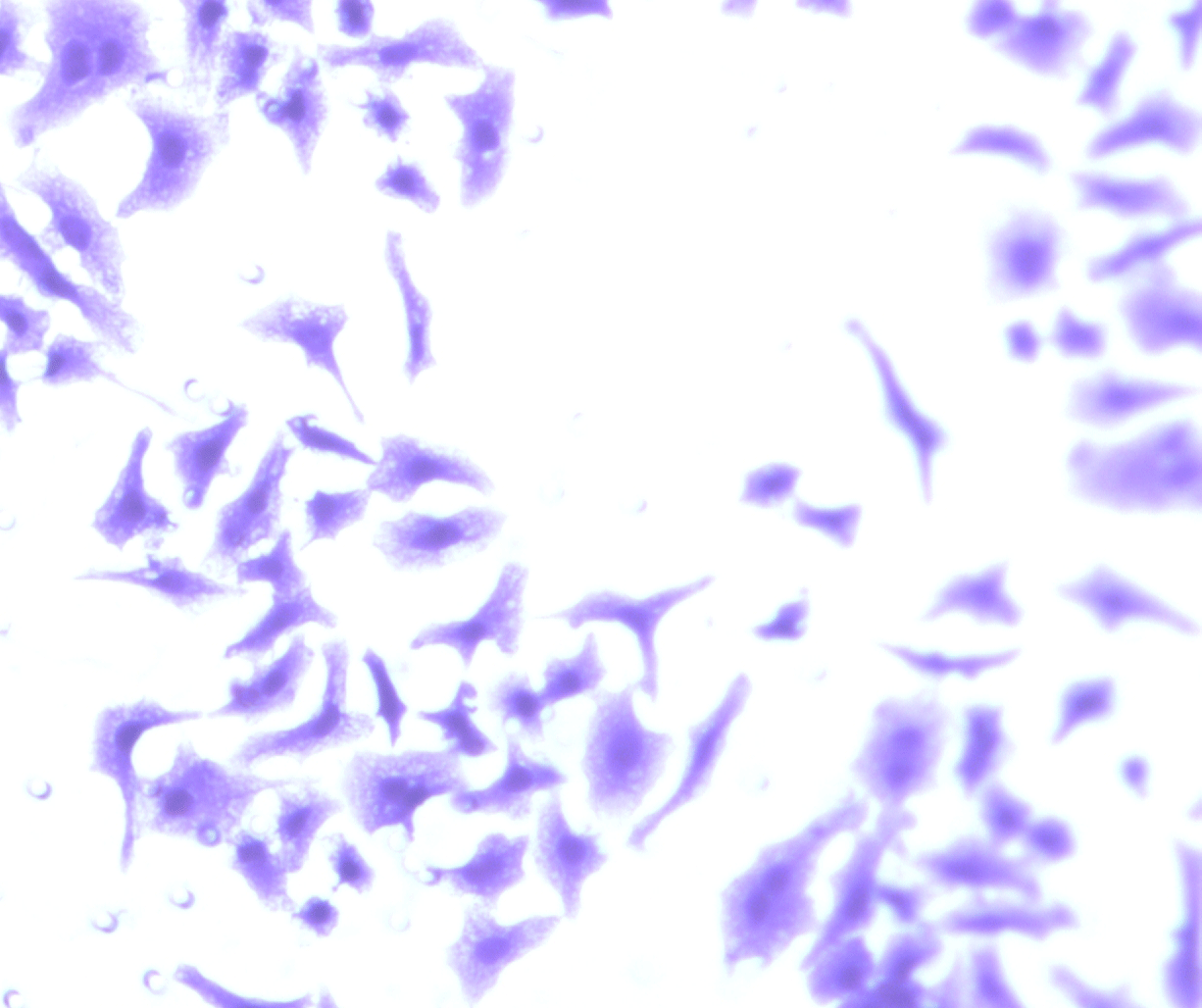

Supplement: Supplementary file 7 [file DataSheet_6.zip › Figure 7D, 7E transwell/Figure 7D migration/AGS/circPTK2+shRNA NC.gif]

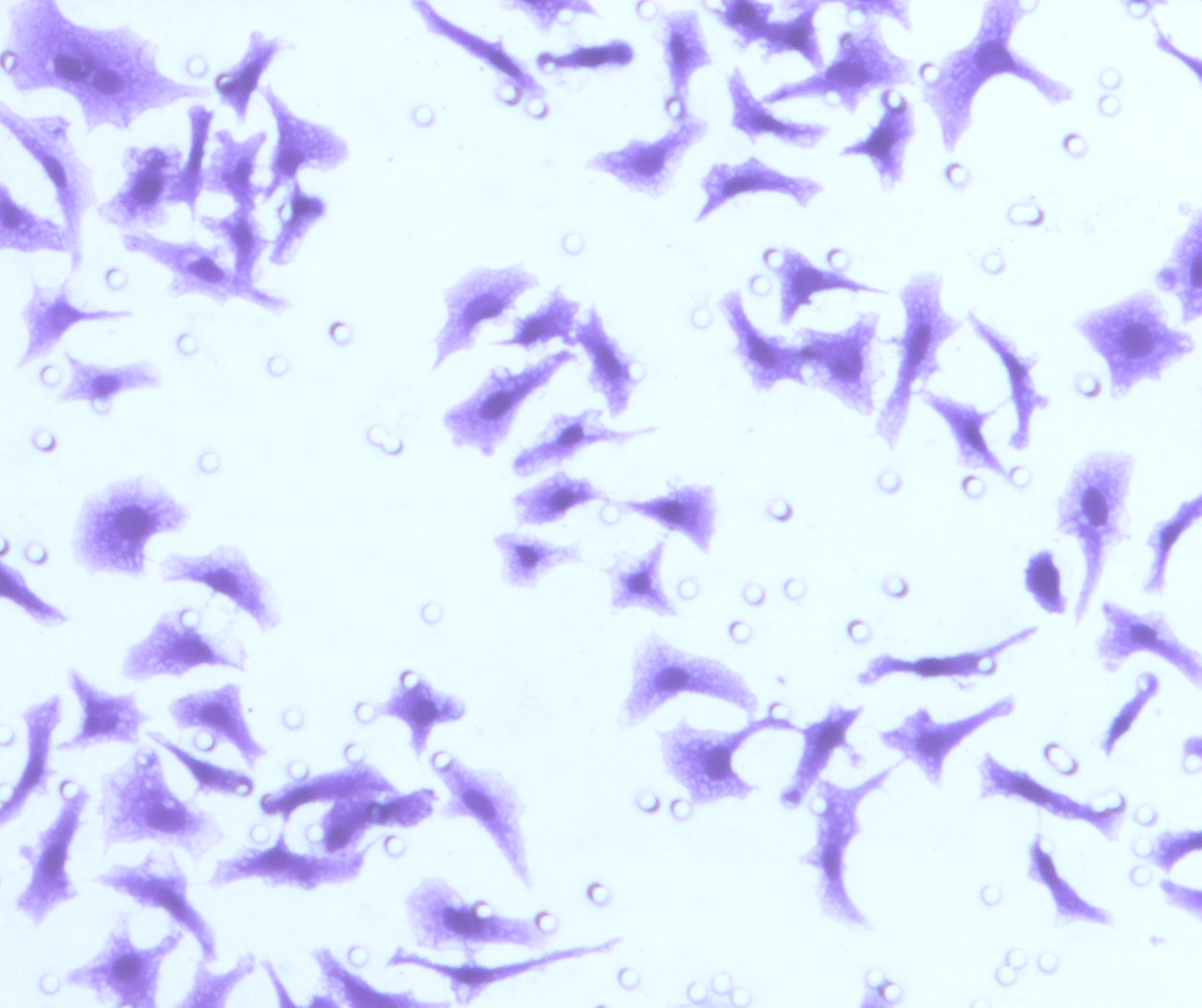

Supplement: Supplementary file 7 [file DataSheet_6.zip › Figure 7D, 7E transwell/Figure 7D migration/AGS/circPTK2.gif]

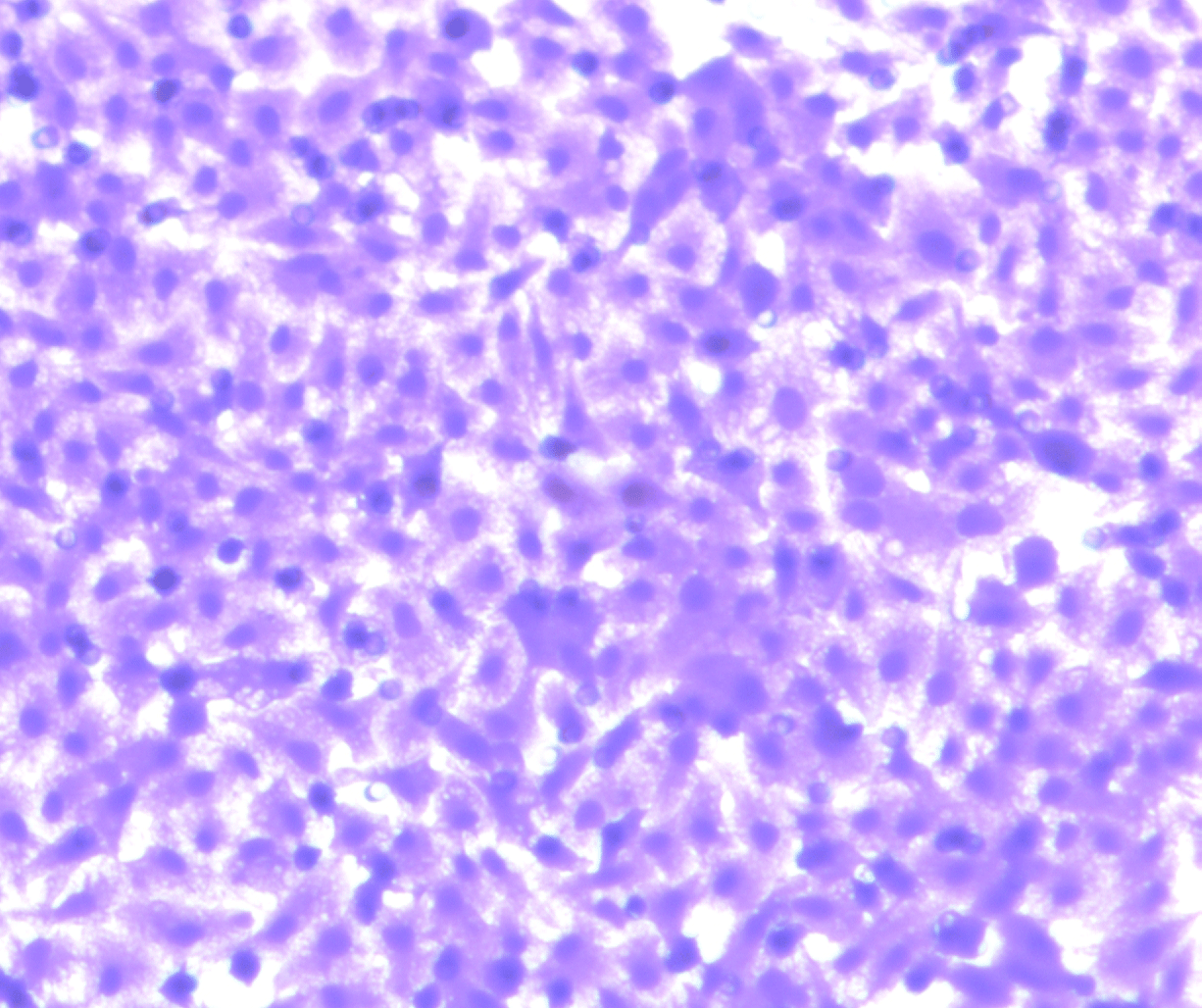

Supplement: Supplementary file 7 [file DataSheet_6.zip › Figure 7D, 7E transwell/Figure 7D migration/AGS/control.gif]

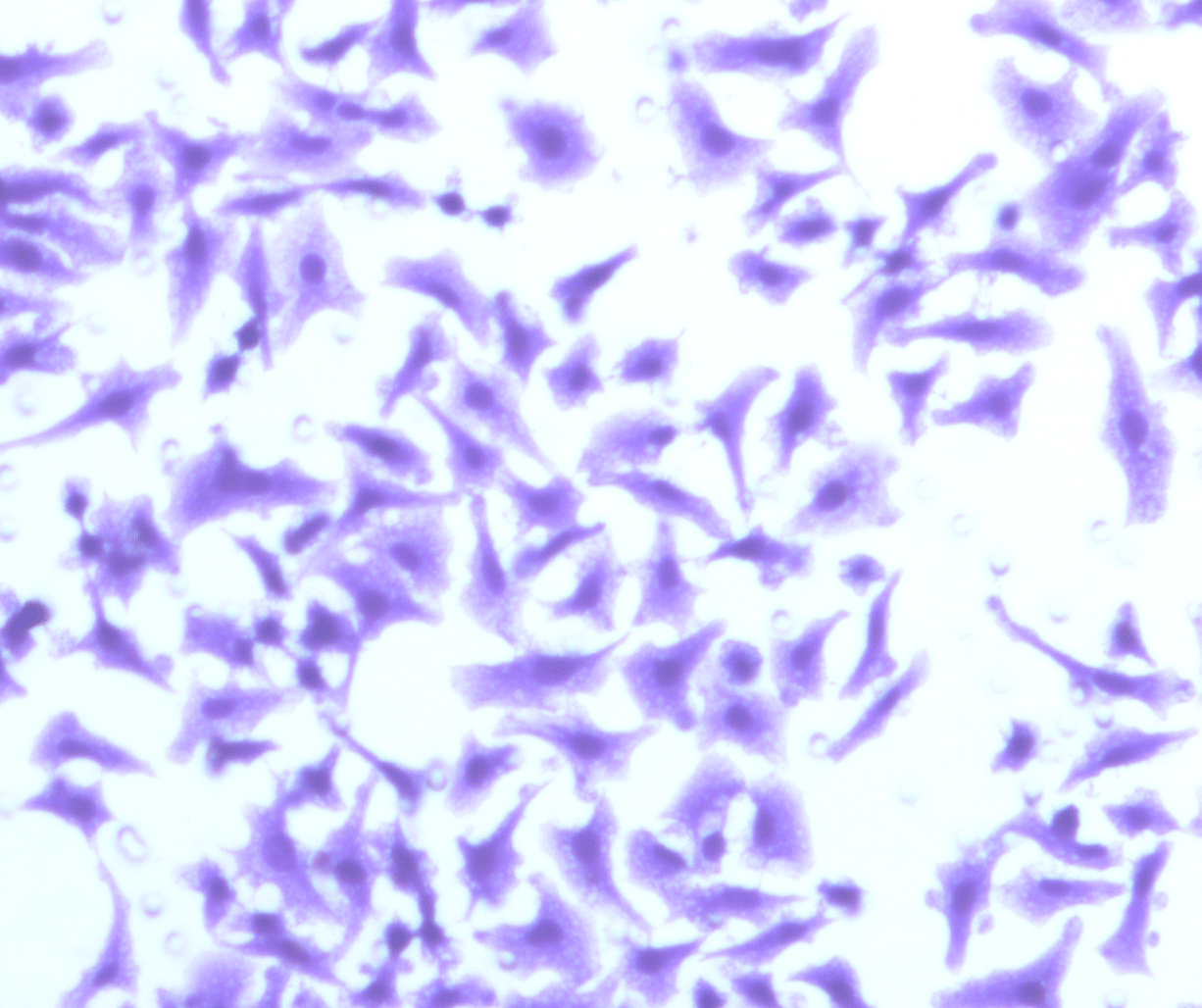

Supplement: Supplementary file 7 [file DataSheet_6.zip › Figure 7D, 7E transwell/Figure 7D migration/MKN45/circPTK2+AATK shRNA.gif]

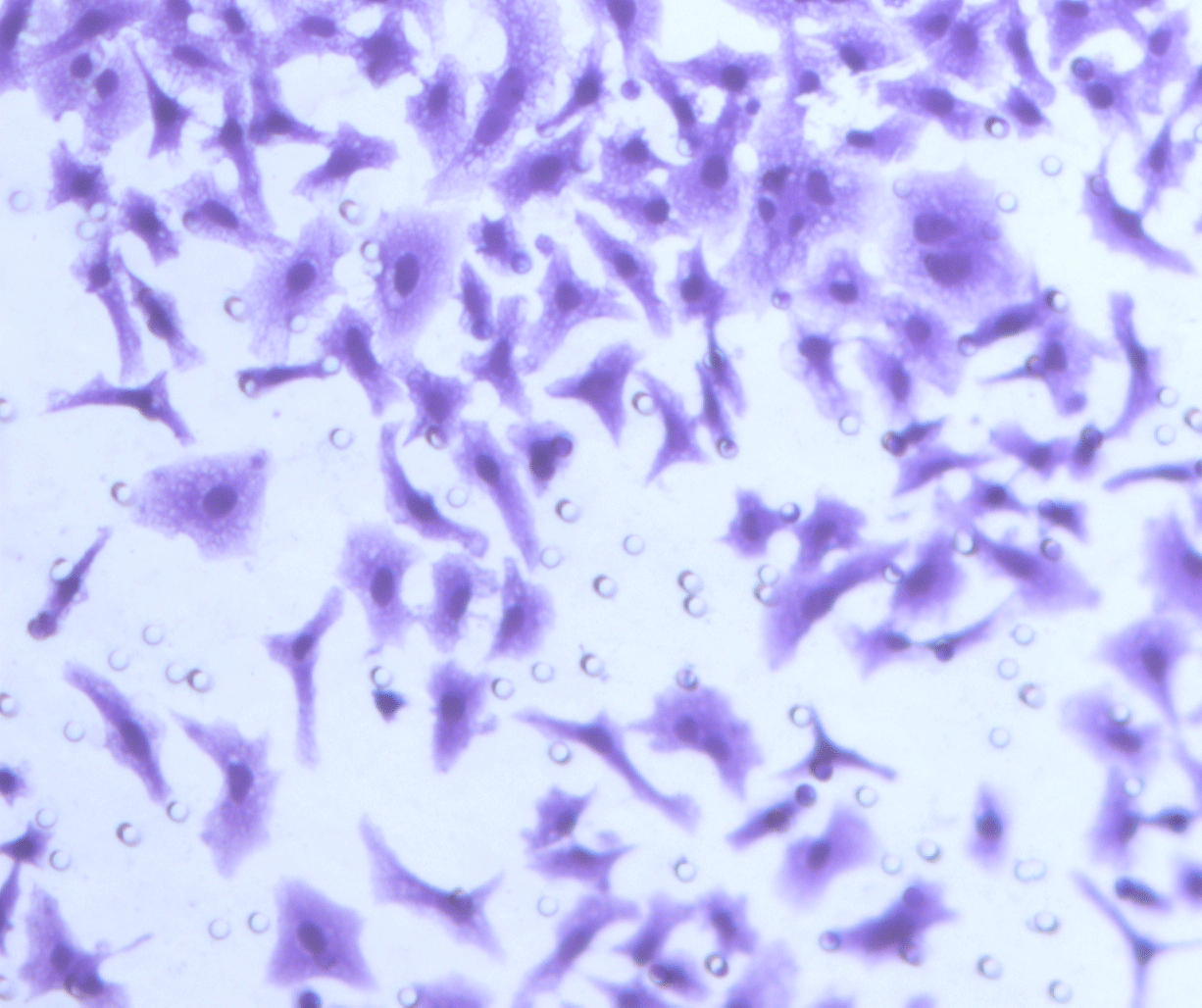

Supplement: Supplementary file 7 [file DataSheet_6.zip › Figure 7D, 7E transwell/Figure 7D migration/MKN45/circPTK2+miR-196a-3p.gif]

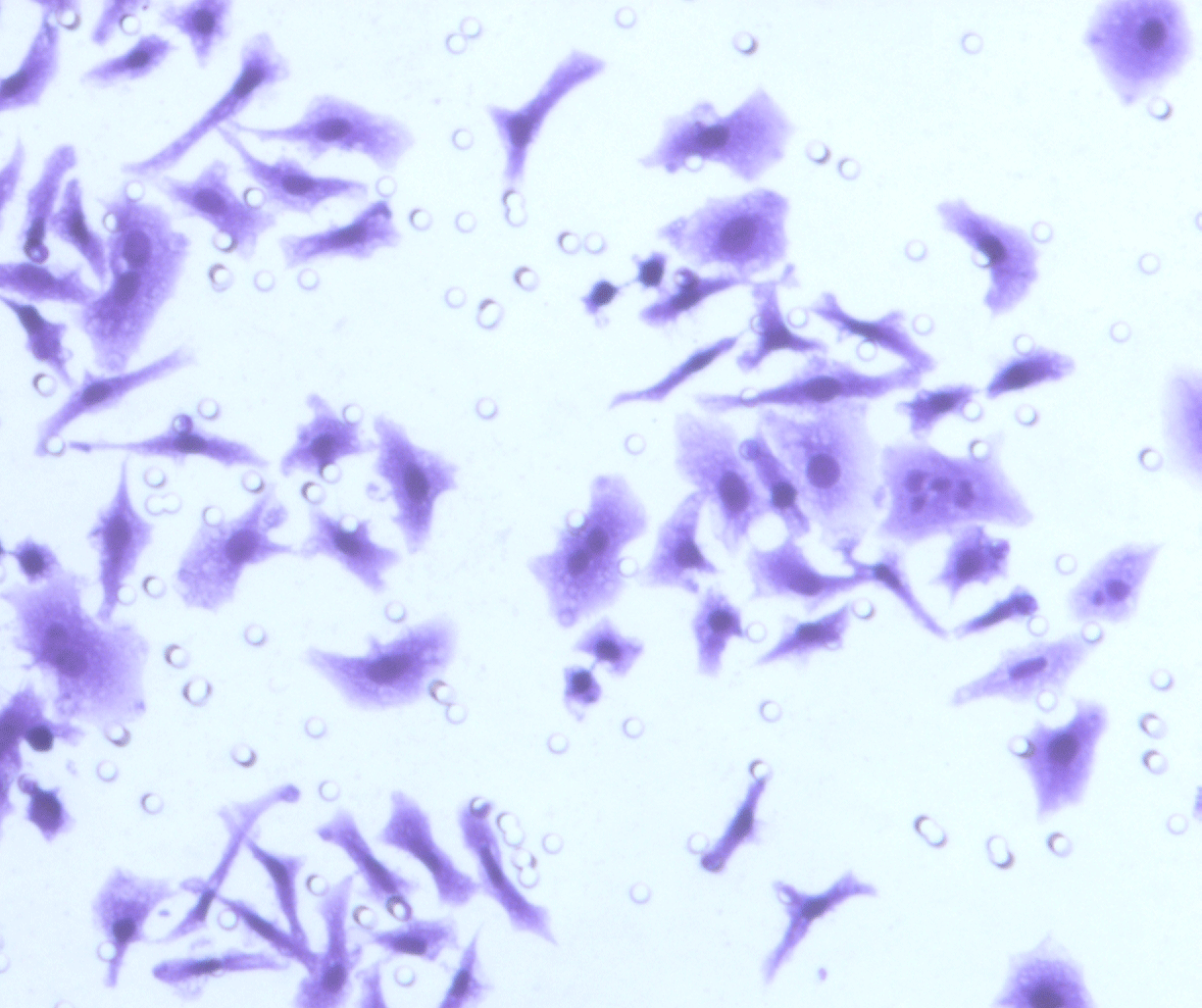

Supplement: Supplementary file 7 [file DataSheet_6.zip › Figure 7D, 7E transwell/Figure 7D migration/MKN45/circPTK2+mimics NC.gif]

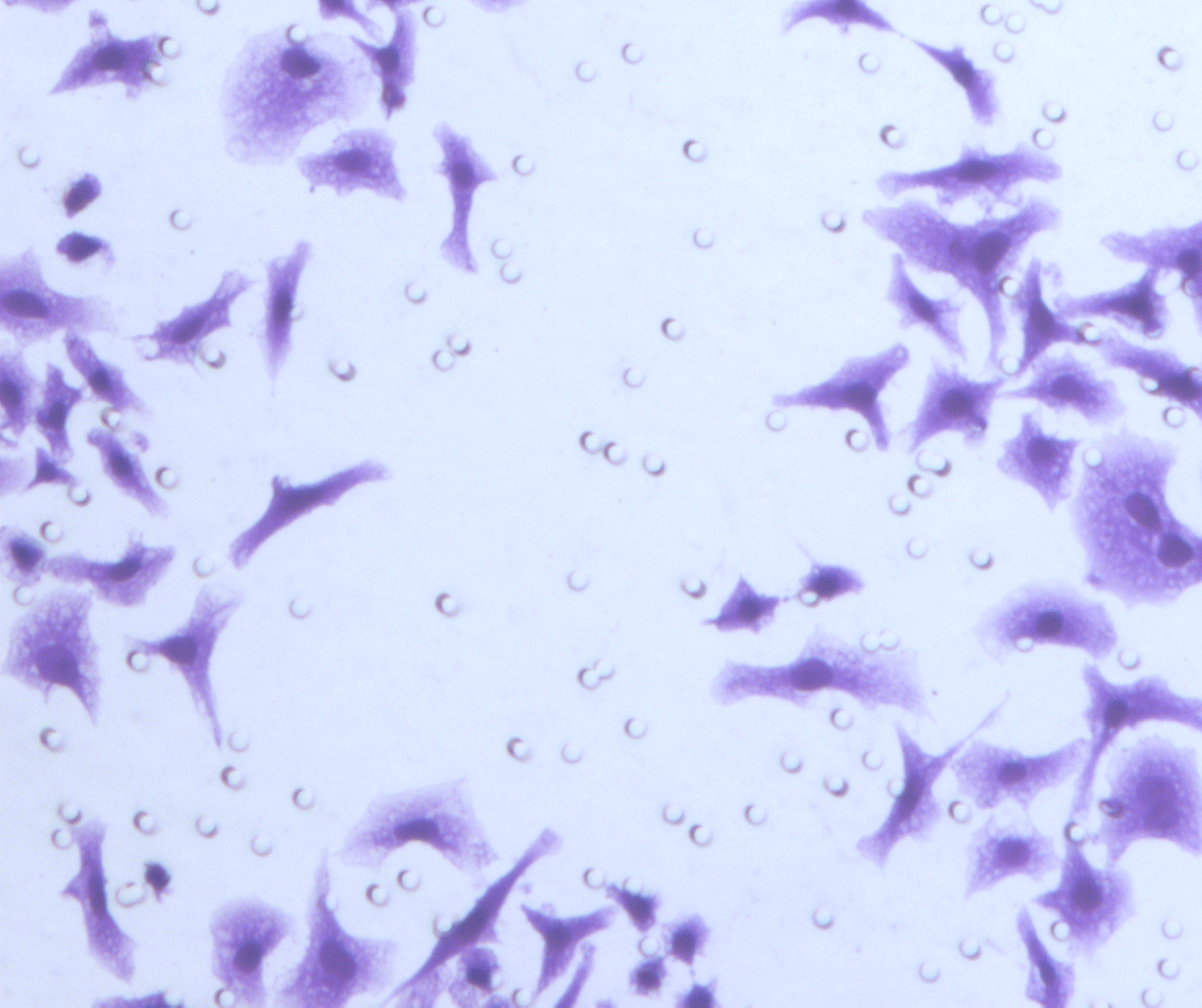

Supplement: Supplementary file 7 [file DataSheet_6.zip › Figure 7D, 7E transwell/Figure 7D migration/MKN45/circPTK2+shRNA NC.gif]

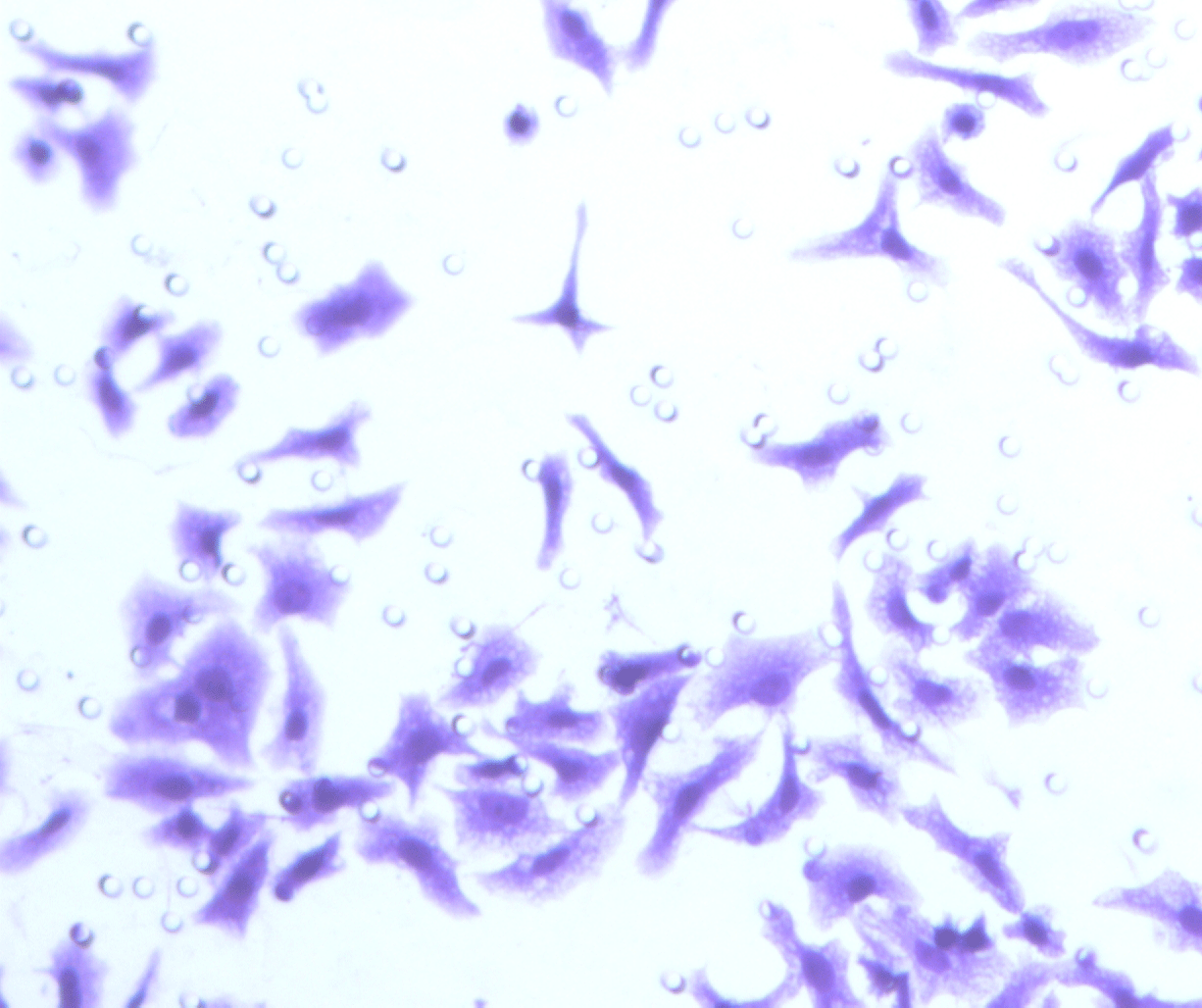

Supplement: Supplementary file 7 [file DataSheet_6.zip › Figure 7D, 7E transwell/Figure 7D migration/MKN45/circPTK2.gif]

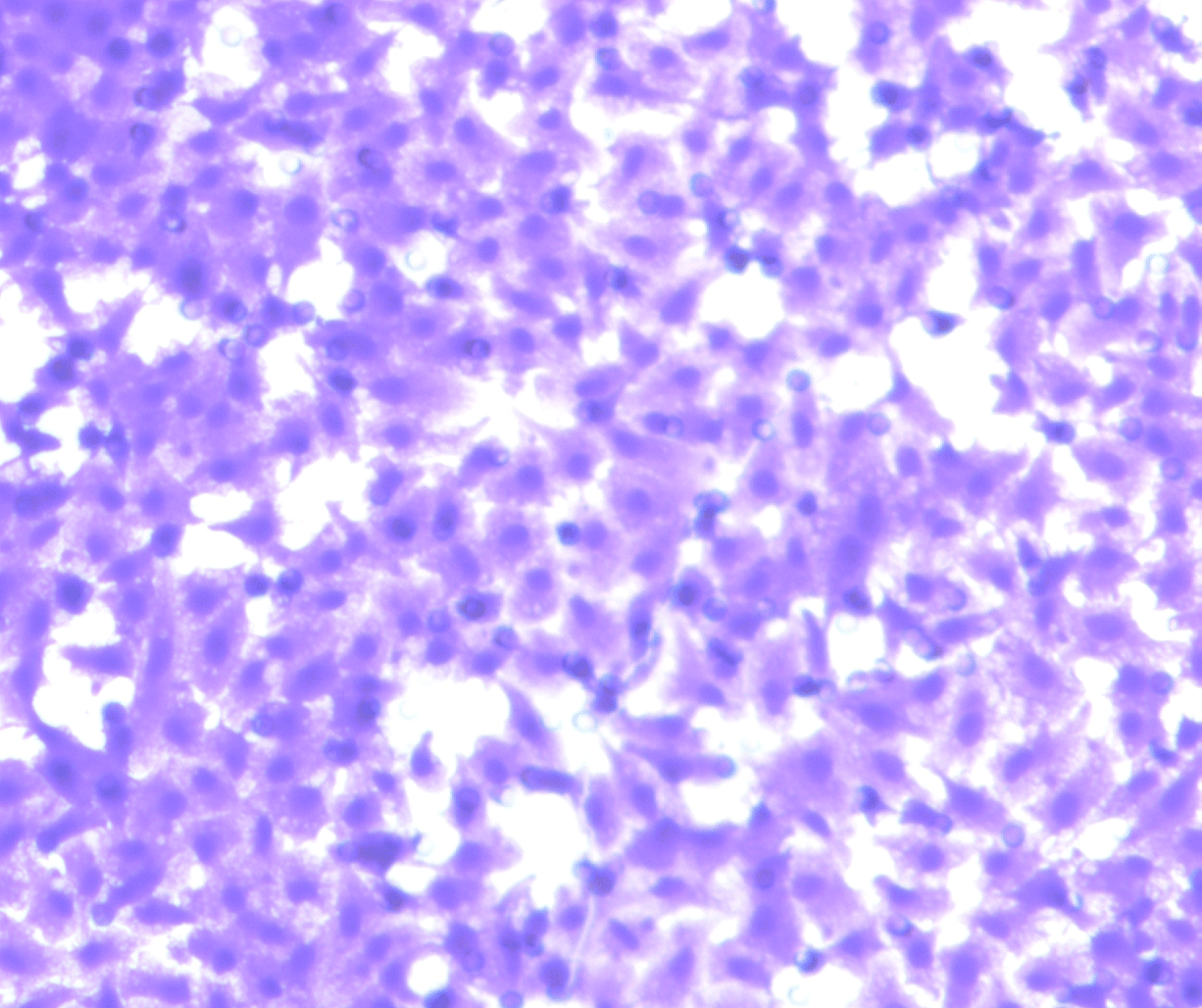

Supplement: Supplementary file 7 [file DataSheet_6.zip › Figure 7D, 7E transwell/Figure 7D migration/MKN45/control.gif]

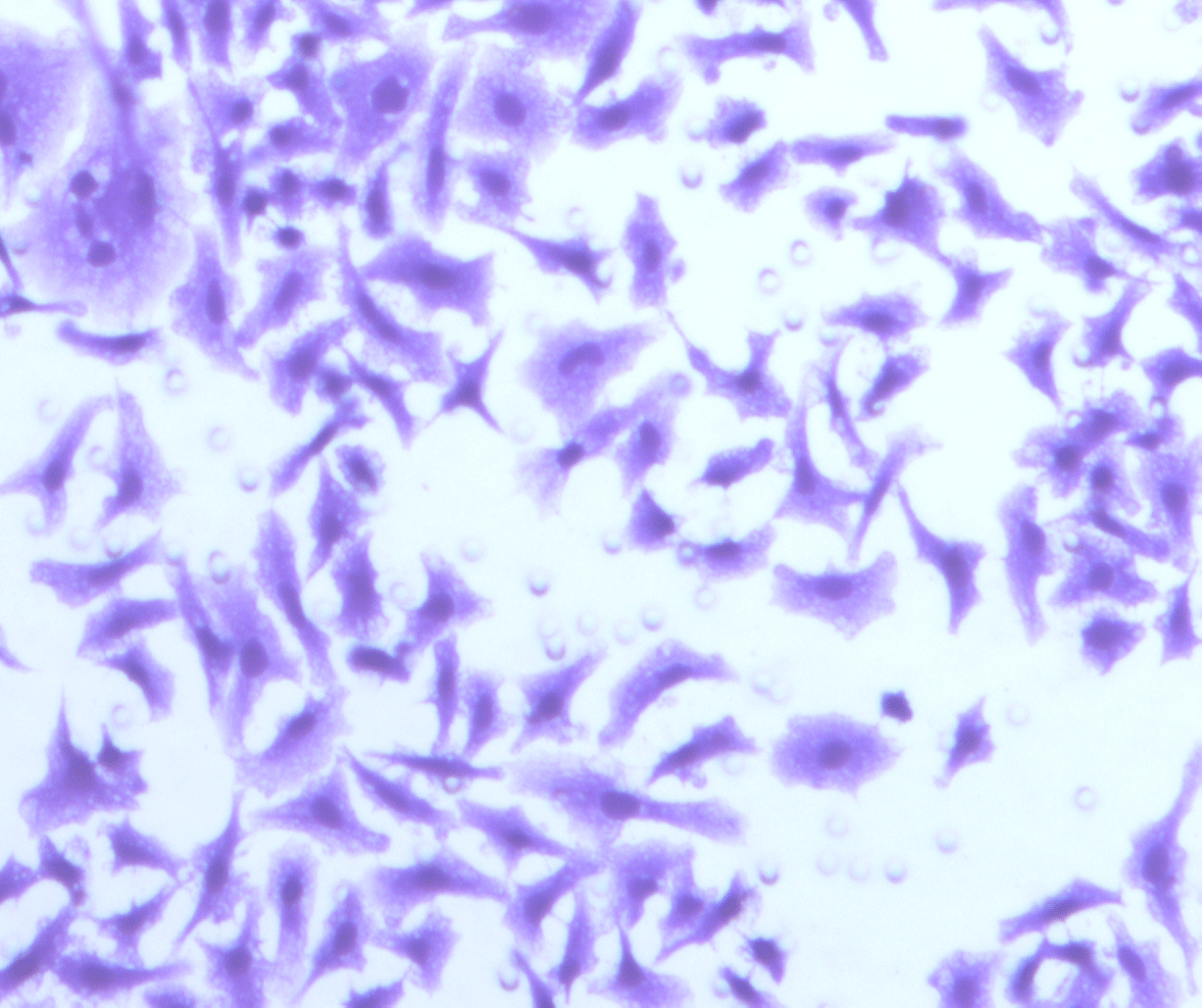

Supplement: Supplementary file 7 [file DataSheet_6.zip › Figure 7D, 7E transwell/Figure 7E invasion/AGS/circPTK2+AATK shRNA.gif]

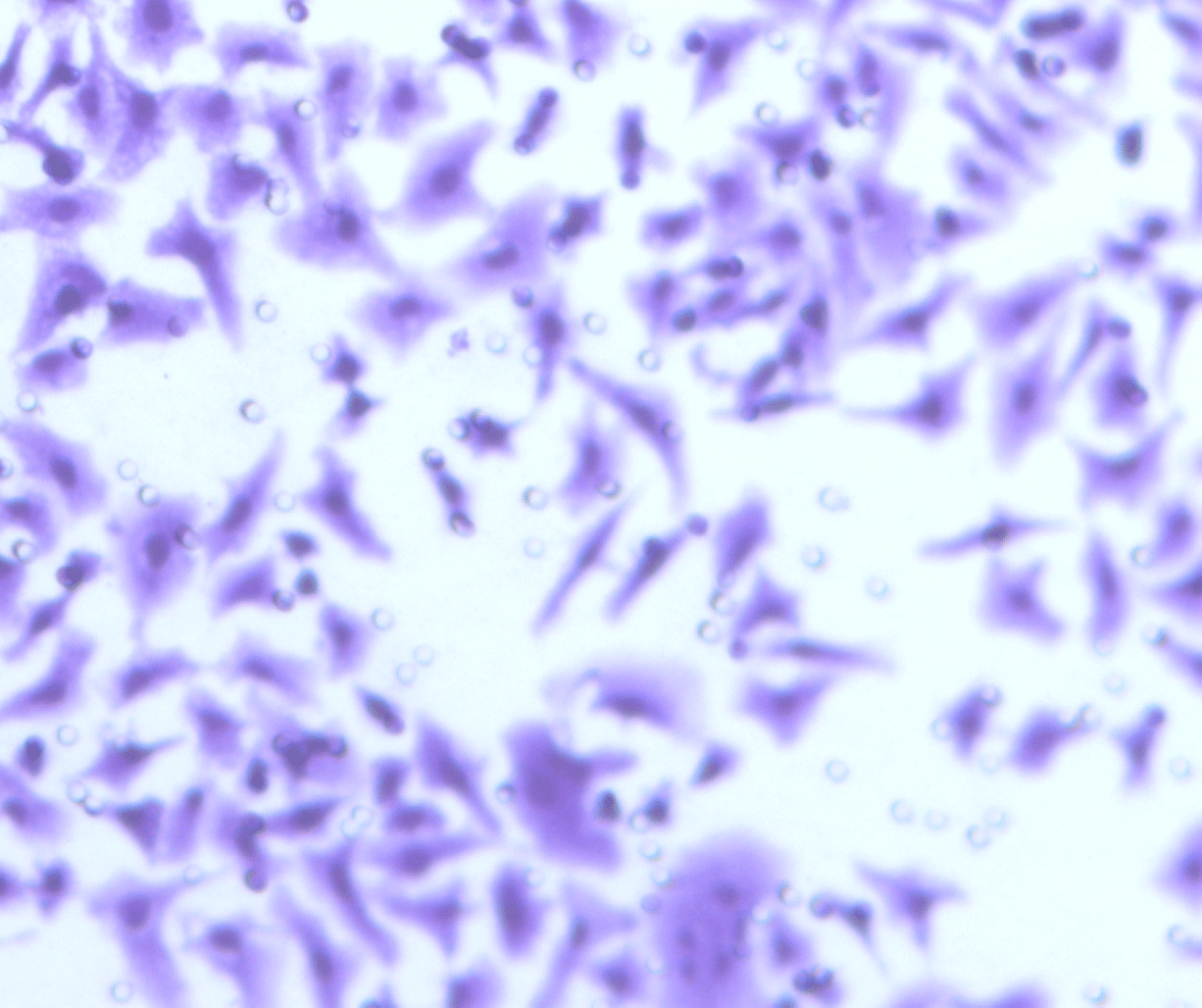

Supplement: Supplementary file 7 [file DataSheet_6.zip › Figure 7D, 7E transwell/Figure 7E invasion/AGS/circPTK2+miR-196a-3p.gif]

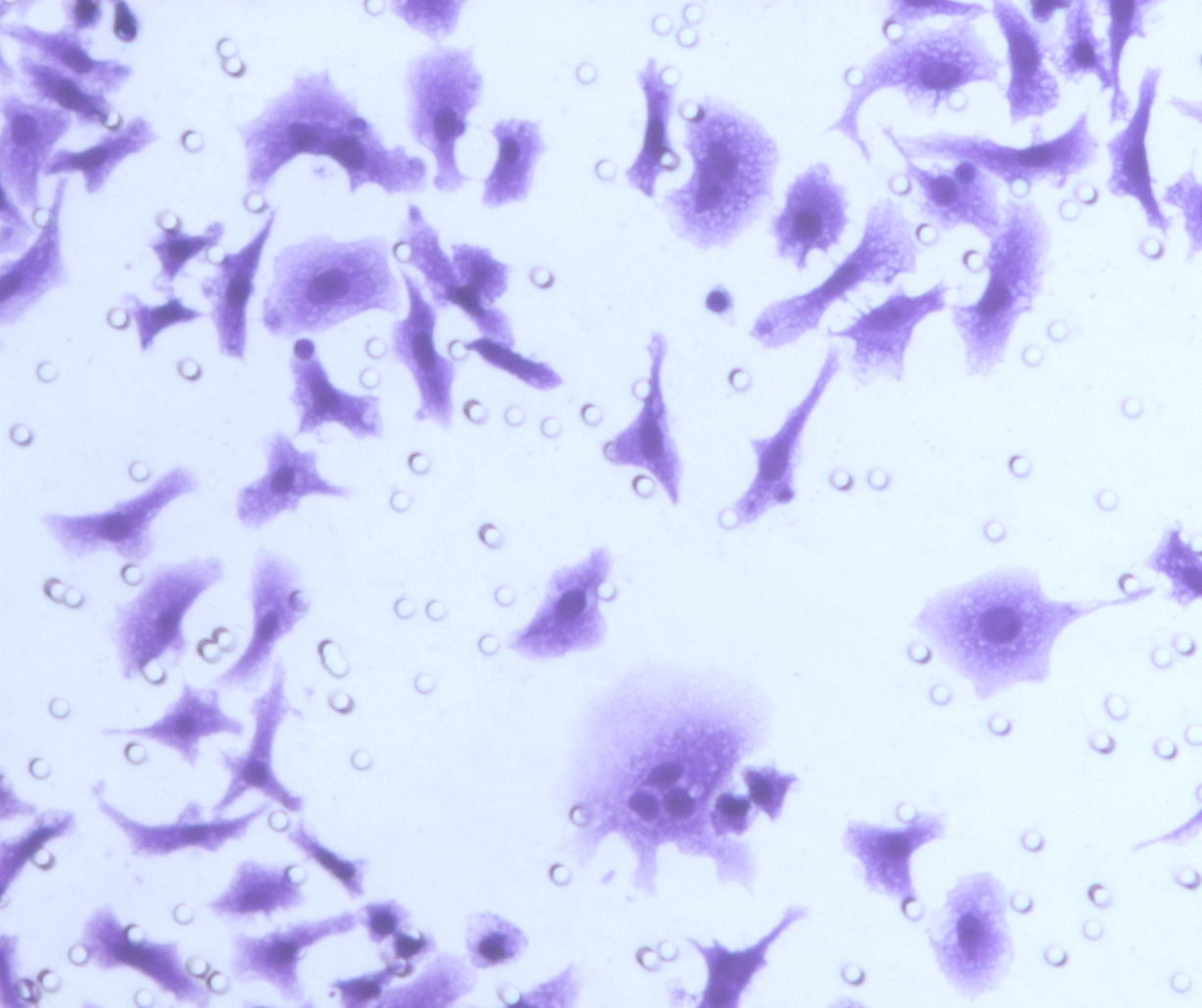

Supplement: Supplementary file 7 [file DataSheet_6.zip › Figure 7D, 7E transwell/Figure 7E invasion/AGS/circPTK2+mimics NC.gif]

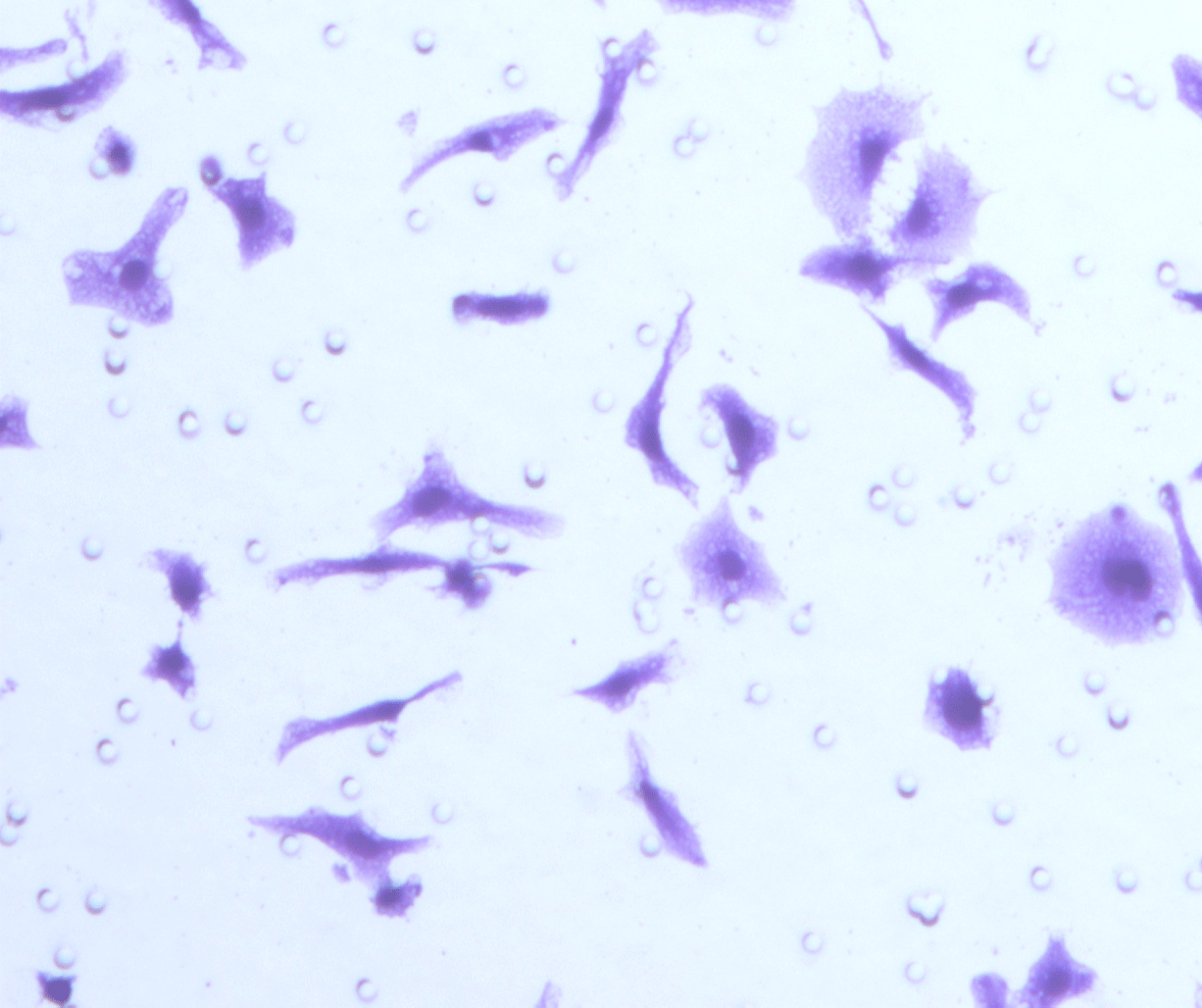

Supplement: Supplementary file 7 [file DataSheet_6.zip › Figure 7D, 7E transwell/Figure 7E invasion/AGS/circPTK2+shRNA NC.gif]

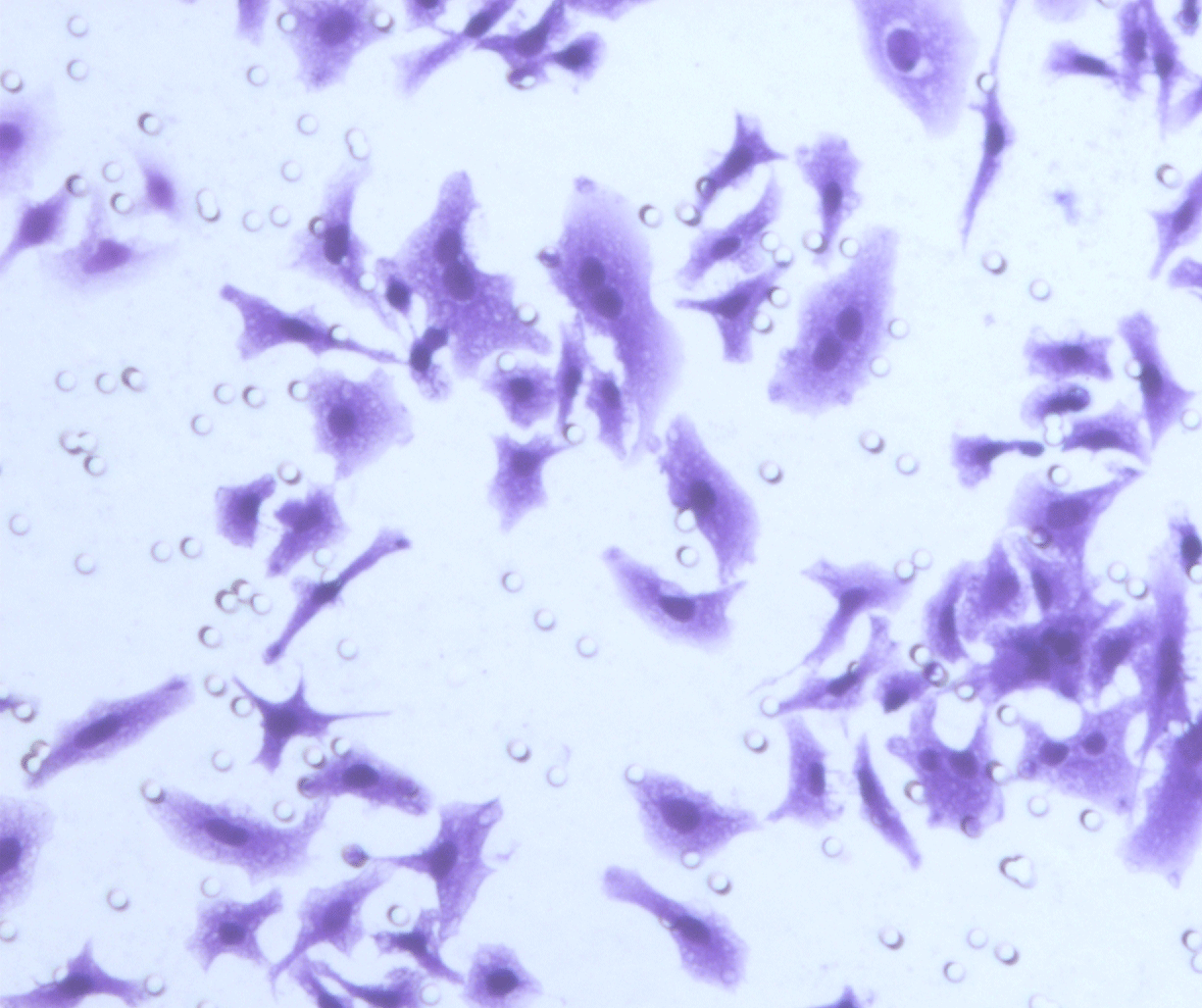

Supplement: Supplementary file 7 [file DataSheet_6.zip › Figure 7D, 7E transwell/Figure 7E invasion/AGS/circPTK2.gif]

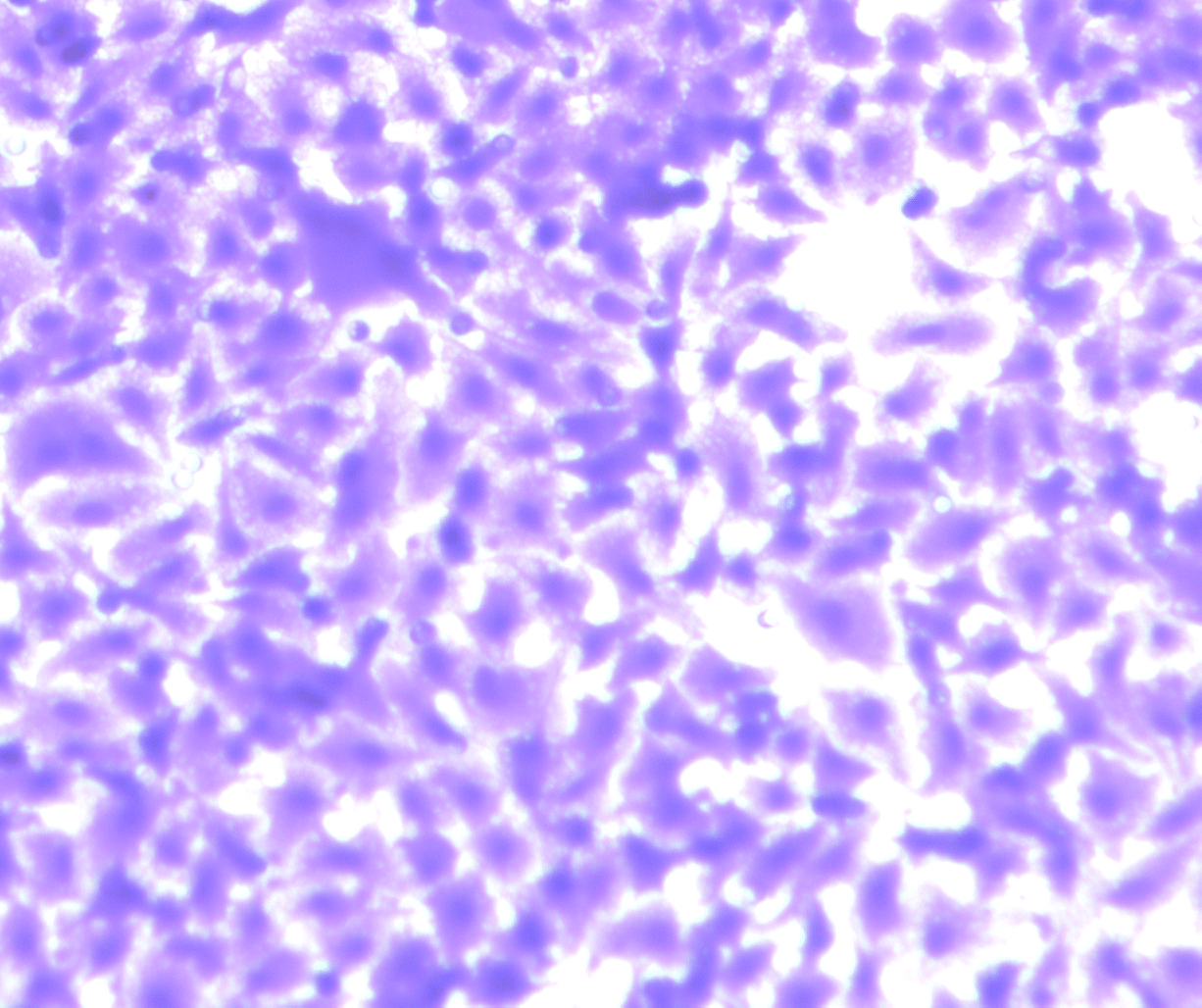

Supplement: Supplementary file 7 [file DataSheet_6.zip › Figure 7D, 7E transwell/Figure 7E invasion/AGS/control.gif]

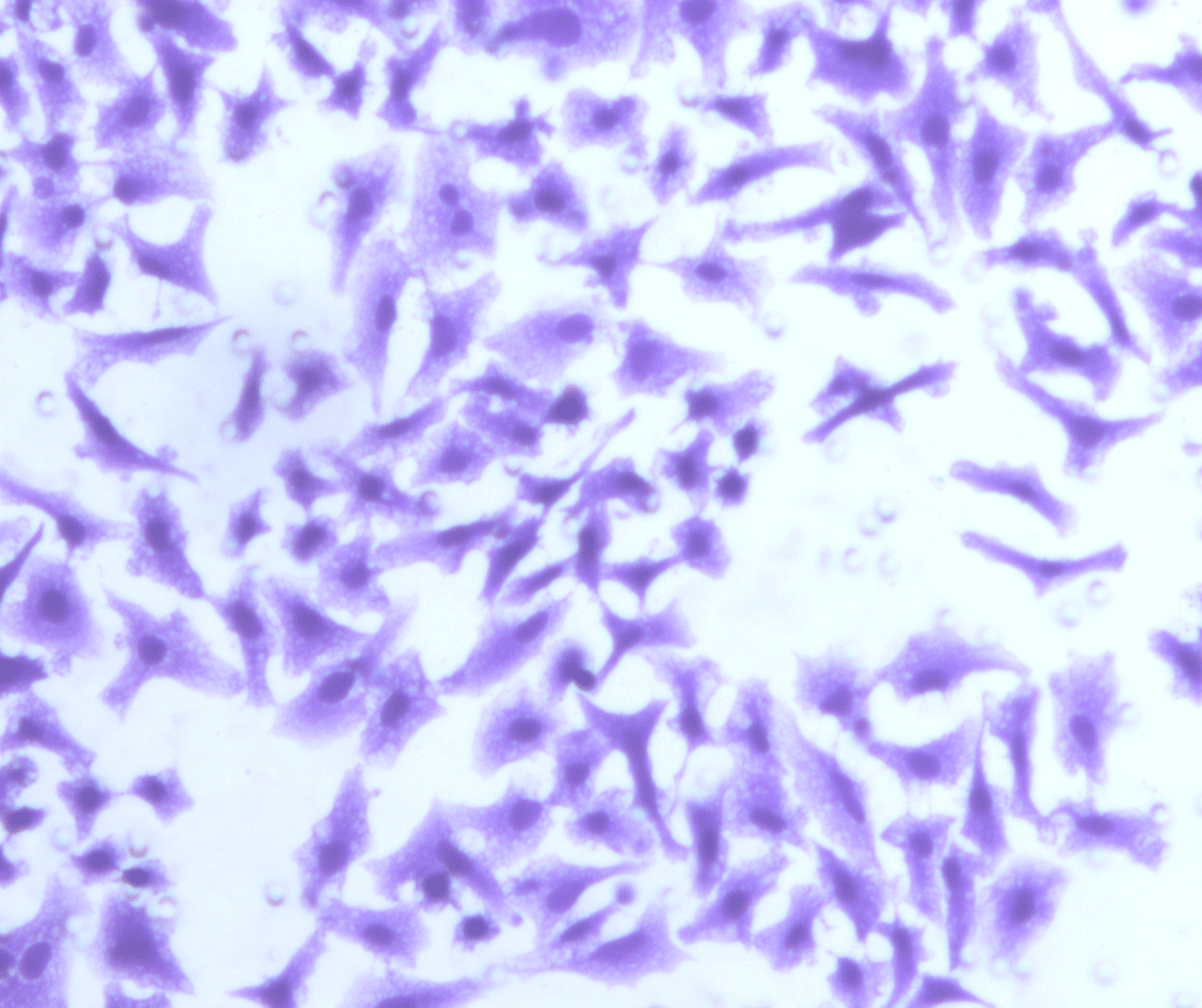

Supplement: Supplementary file 7 [file DataSheet_6.zip › Figure 7D, 7E transwell/Figure 7E invasion/MKN45/circPTK2+AATK shRNA.gif]

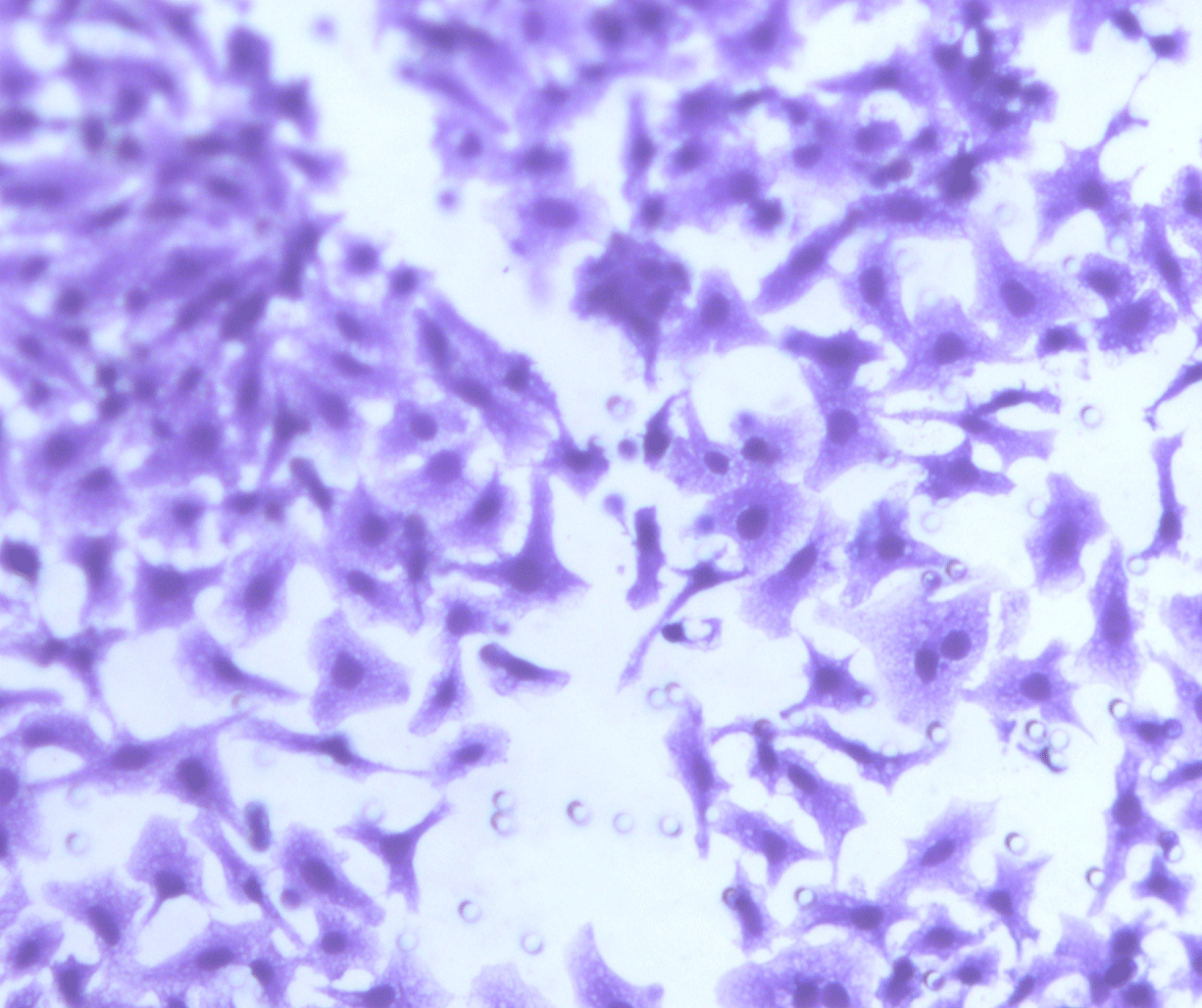

Supplement: Supplementary file 7 [file DataSheet_6.zip › Figure 7D, 7E transwell/Figure 7E invasion/MKN45/circPTK2+miR-196a-3p.gif]

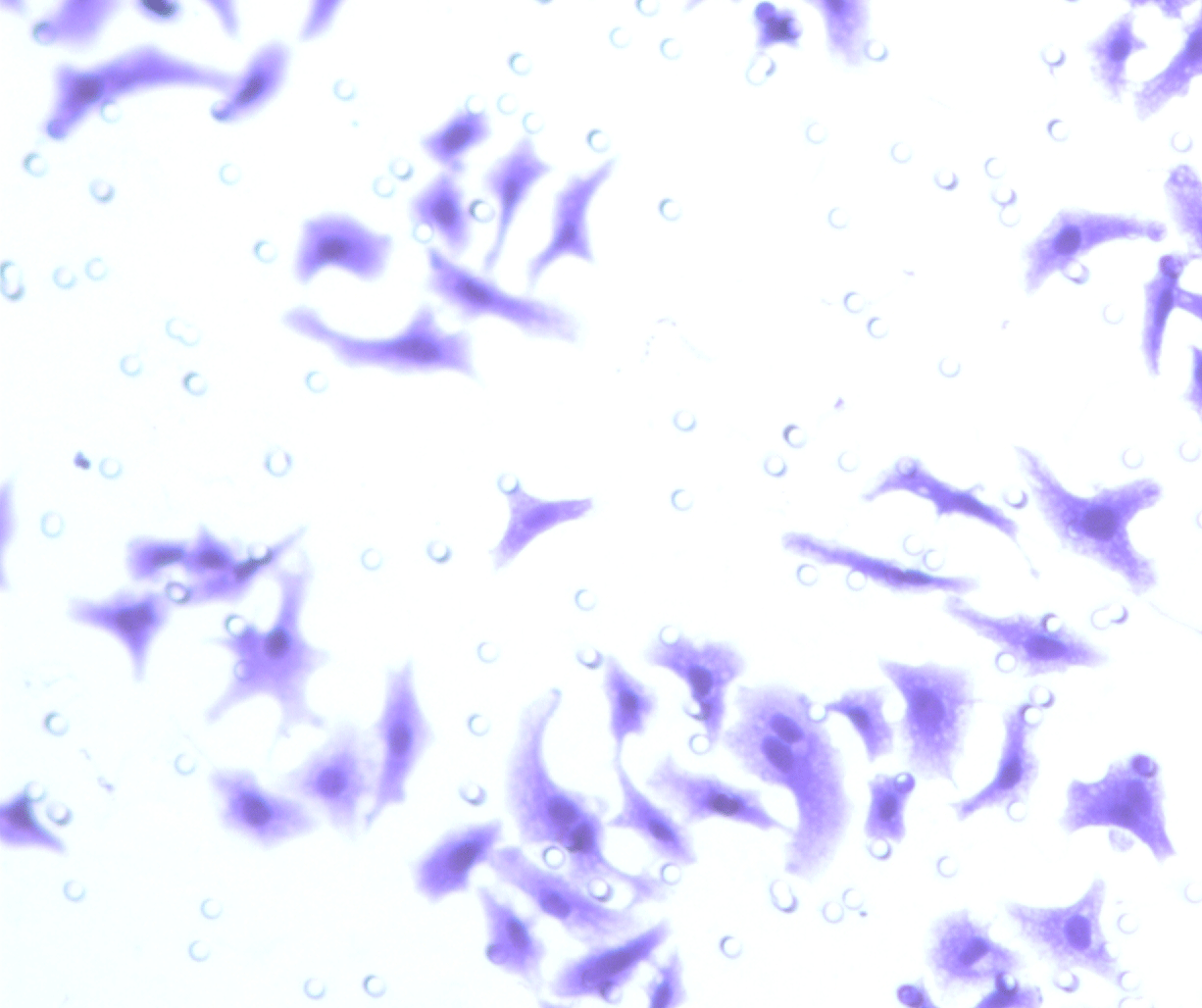

Supplement: Supplementary file 7 [file DataSheet_6.zip › Figure 7D, 7E transwell/Figure 7E invasion/MKN45/circPTK2+mimics NC.gif]

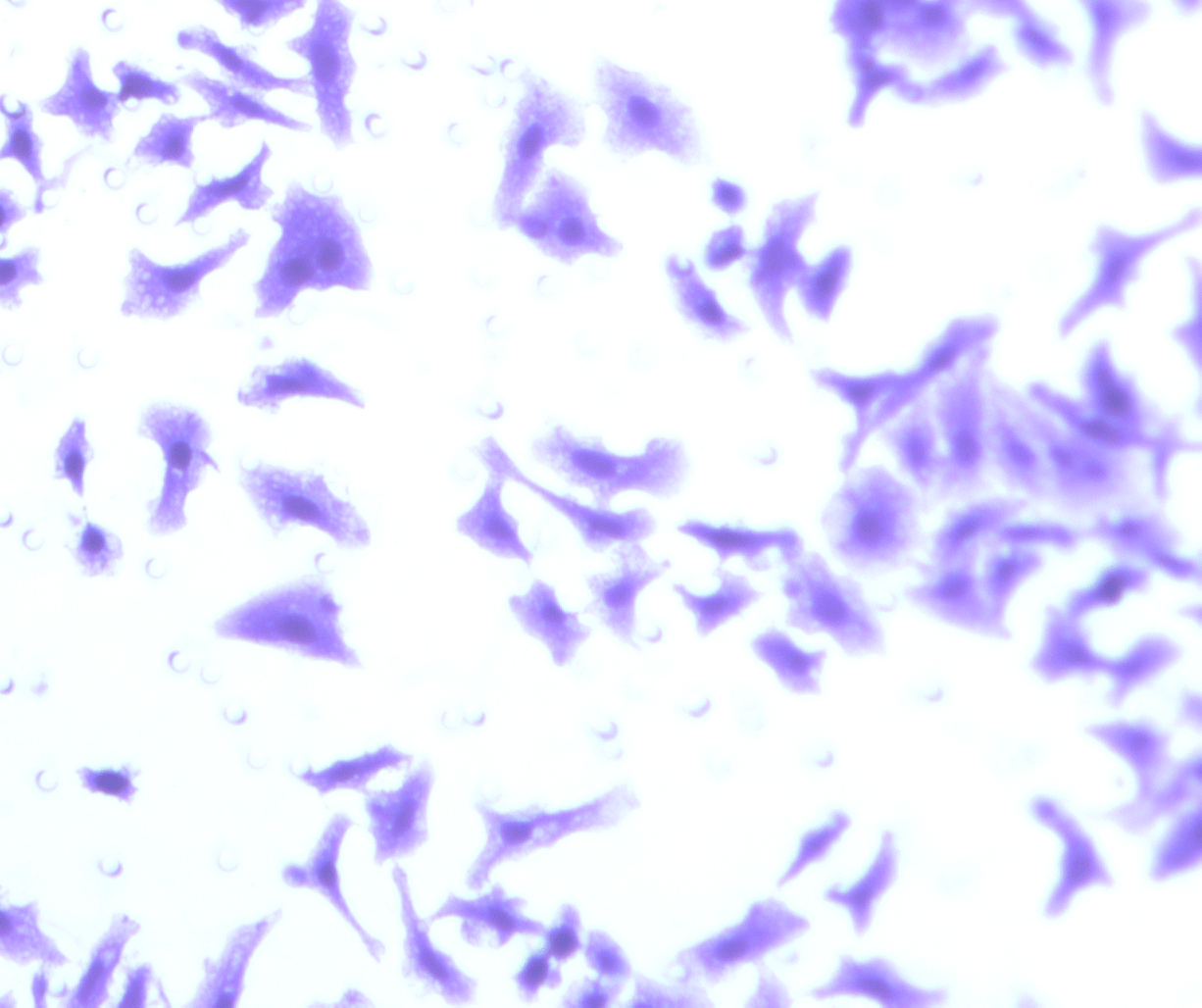

Supplement: Supplementary file 7 [file DataSheet_6.zip › Figure 7D, 7E transwell/Figure 7E invasion/MKN45/circPTK2+shRNA NC.gif]

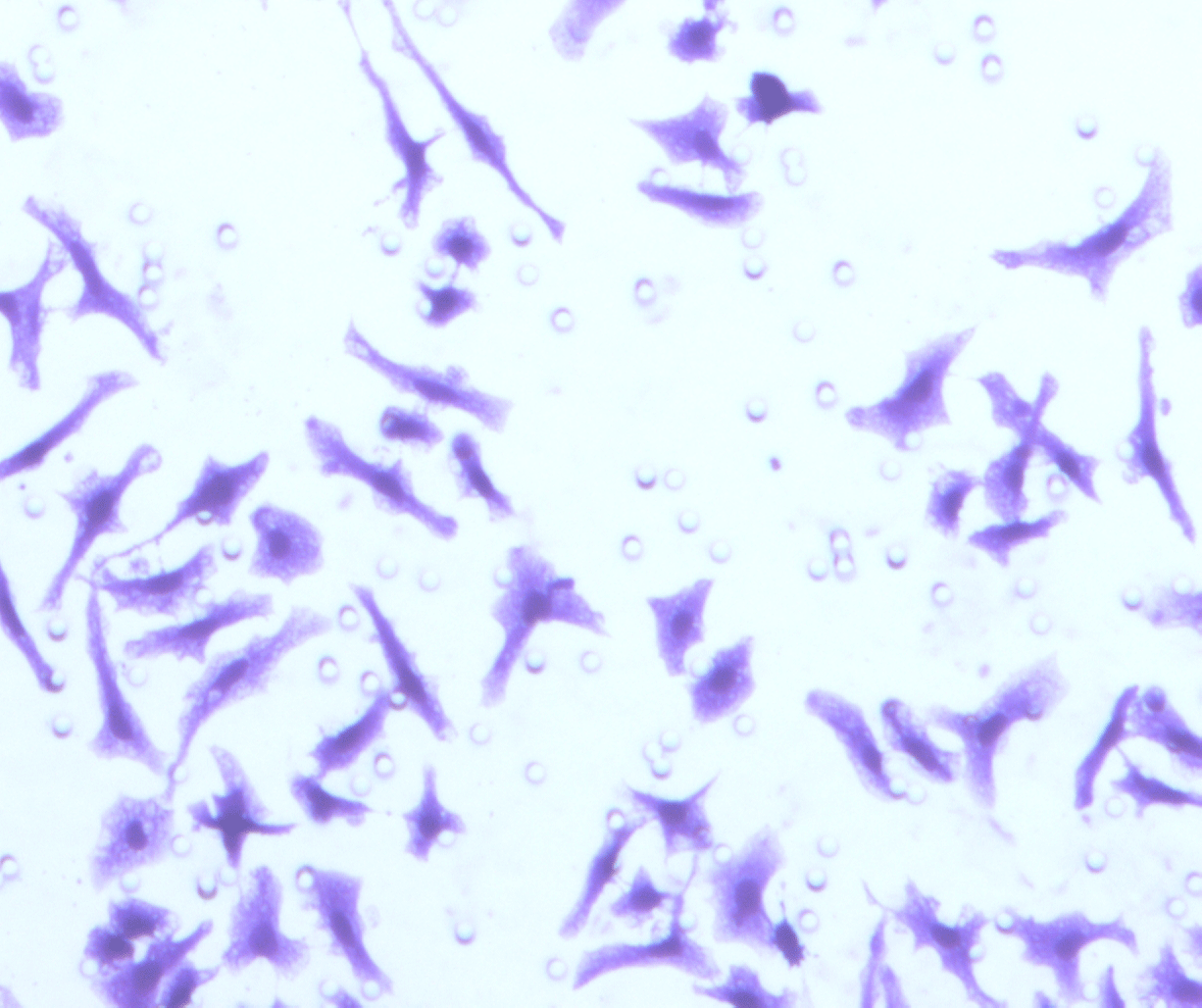

Supplement: Supplementary file 7 [file DataSheet_6.zip › Figure 7D, 7E transwell/Figure 7E invasion/MKN45/circPTK2.gif]

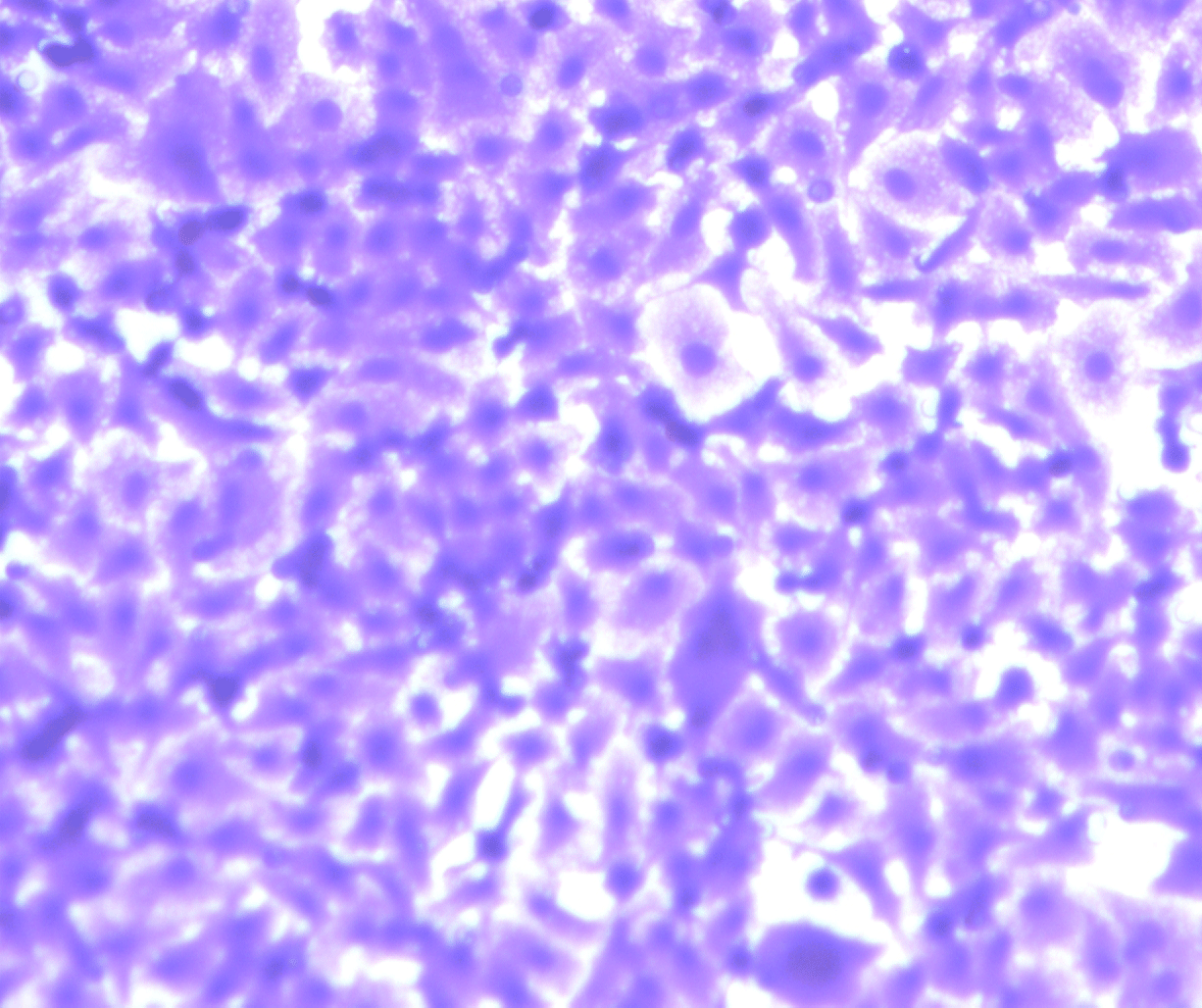

Supplement: Supplementary file 7 [file DataSheet_6.zip › Figure 7D, 7E transwell/Figure 7E invasion/MKN45/control.gif]

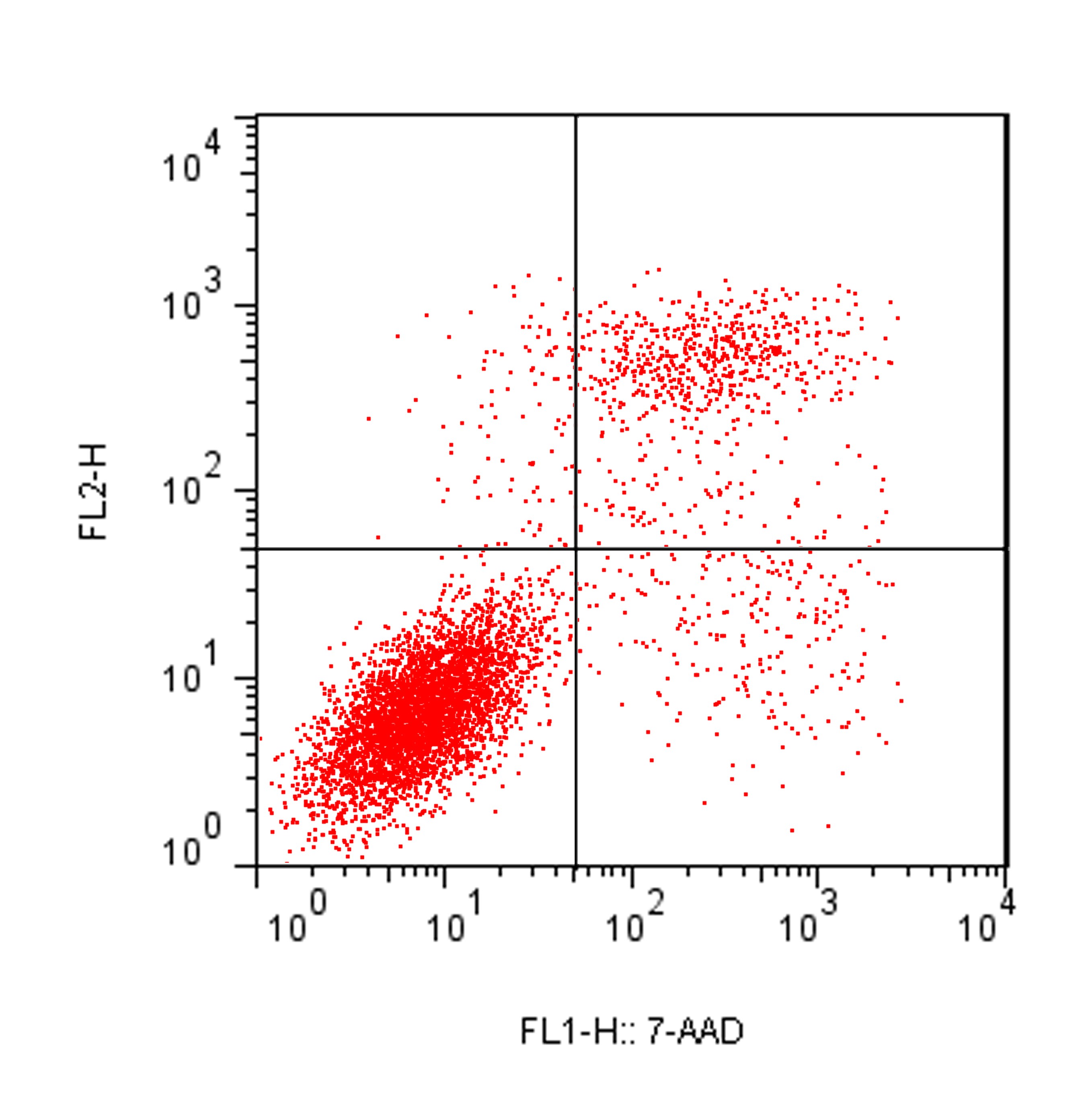

Supplement: Supplementary file 8 [file DataSheet_7.zip › Figure 7F flow cytometry/AGS/circPTK2+AATK shRNA.jpg]

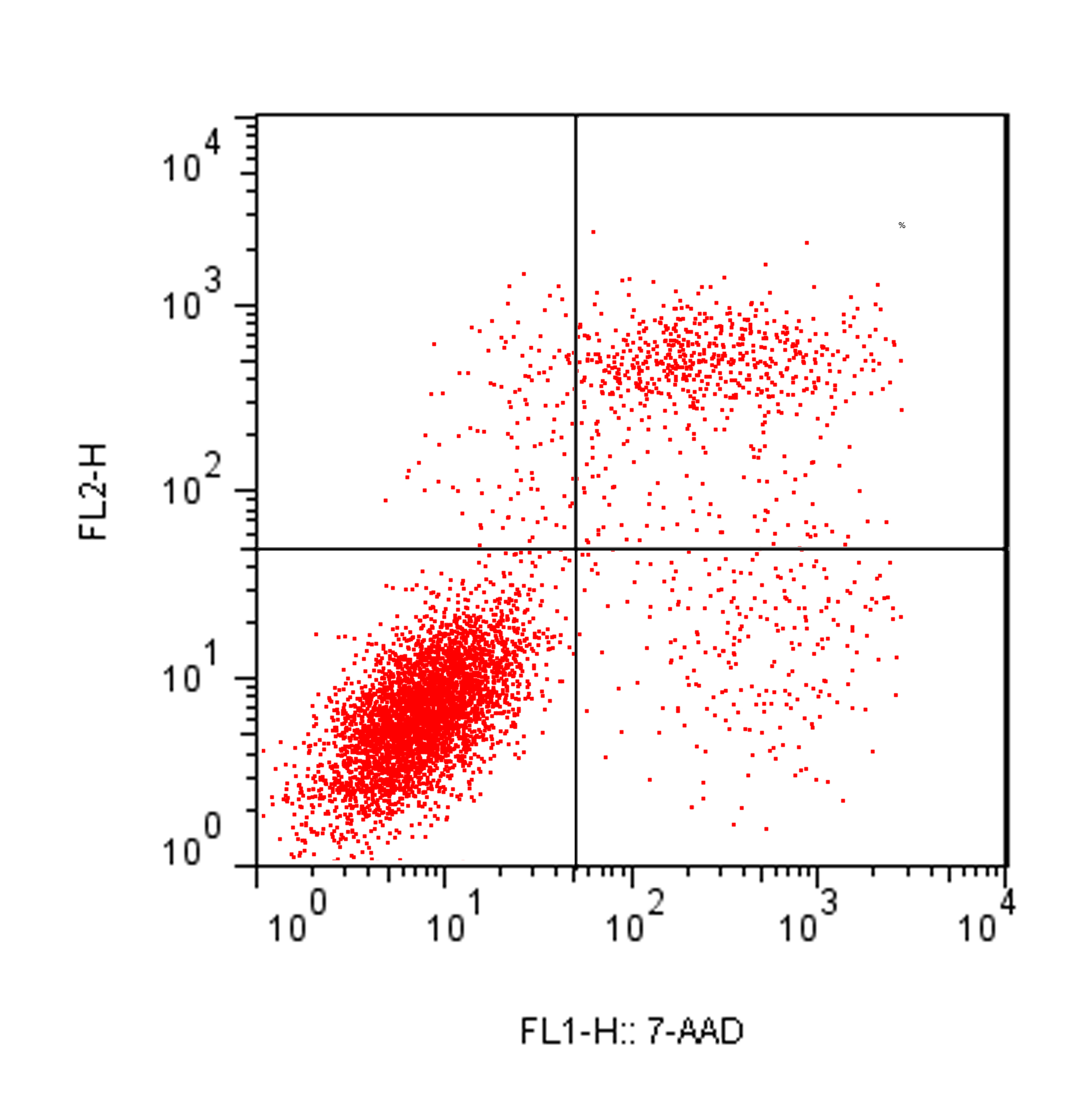

Supplement: Supplementary file 8 [file DataSheet_7.zip › Figure 7F flow cytometry/AGS/circPTK2+miR-196a-3p.jpg]

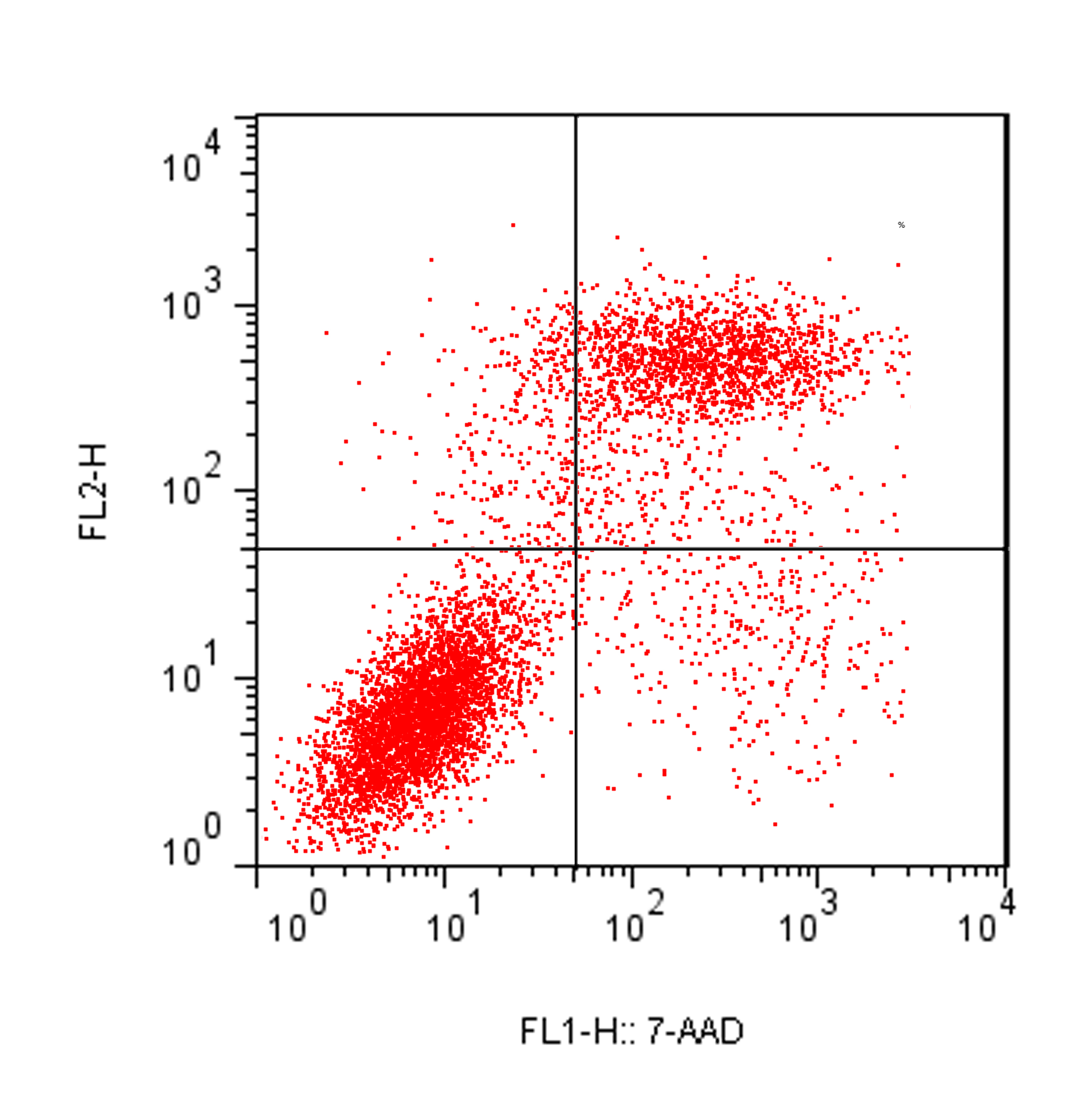

Supplement: Supplementary file 8 [file DataSheet_7.zip › Figure 7F flow cytometry/AGS/circPTK2+mimics NC.jpg]

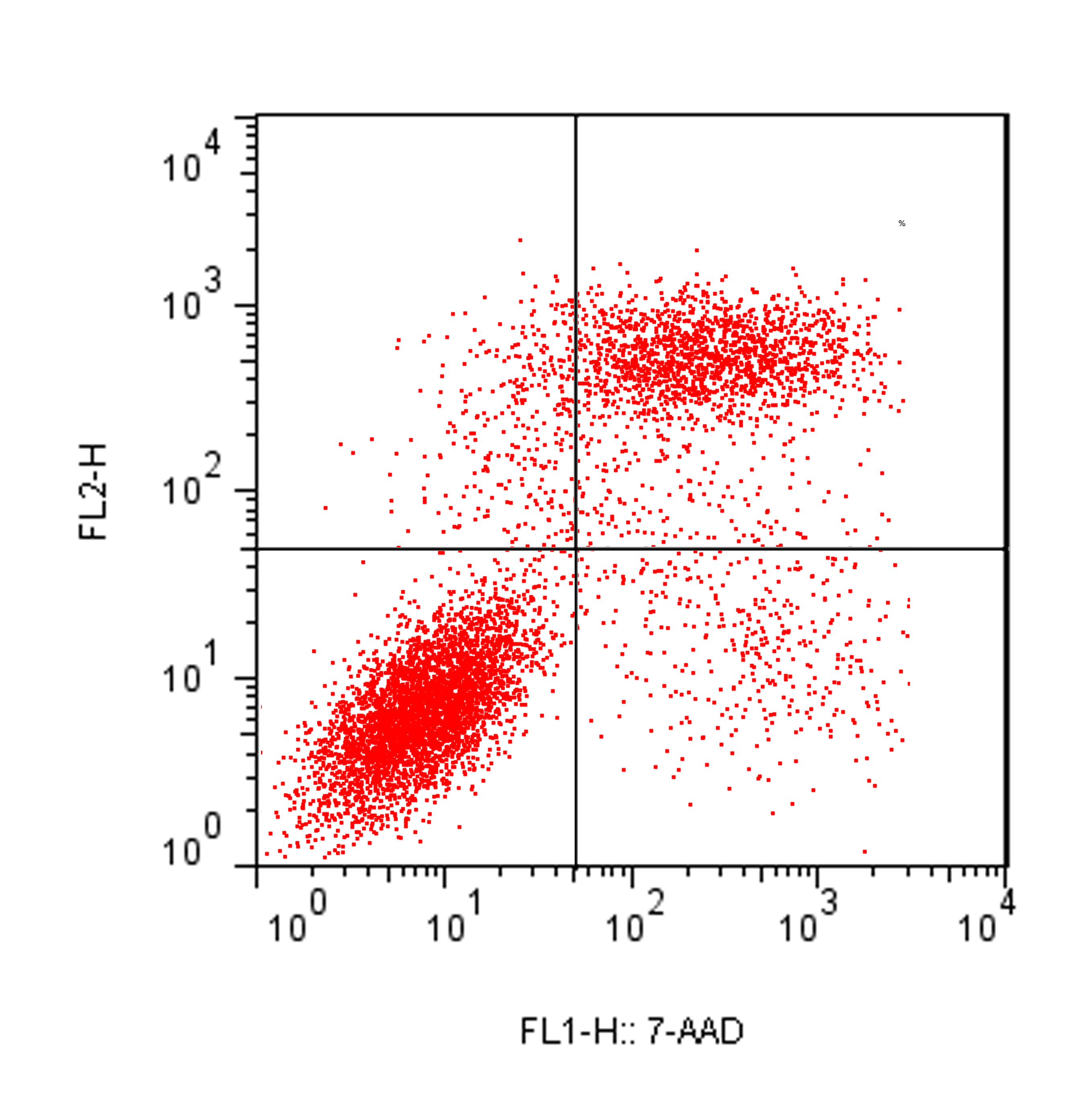

Supplement: Supplementary file 8 [file DataSheet_7.zip › Figure 7F flow cytometry/AGS/circPTK2+shRNA NC.jpg]

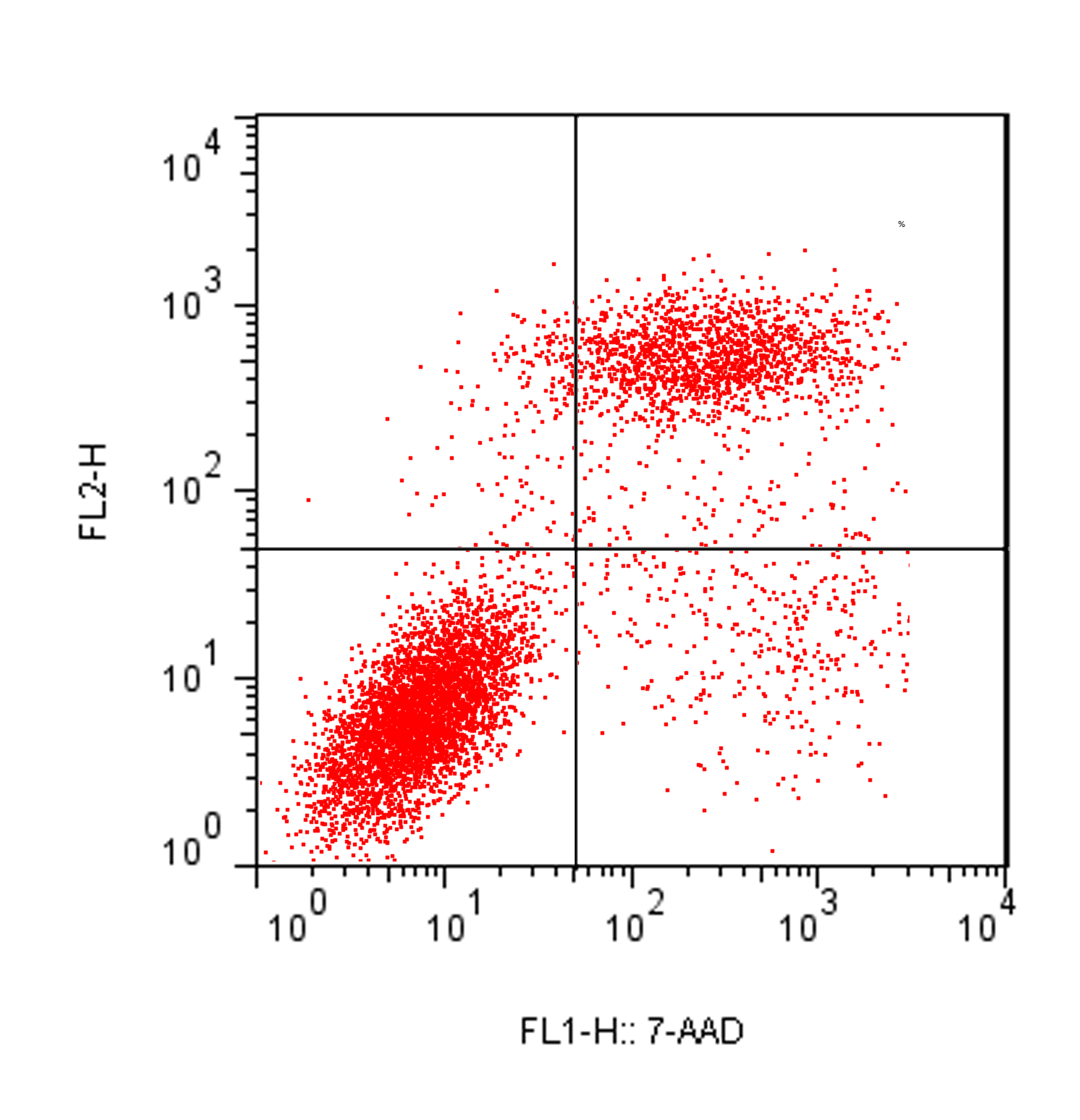

Supplement: Supplementary file 8 [file DataSheet_7.zip › Figure 7F flow cytometry/AGS/circPTK2.jpg]

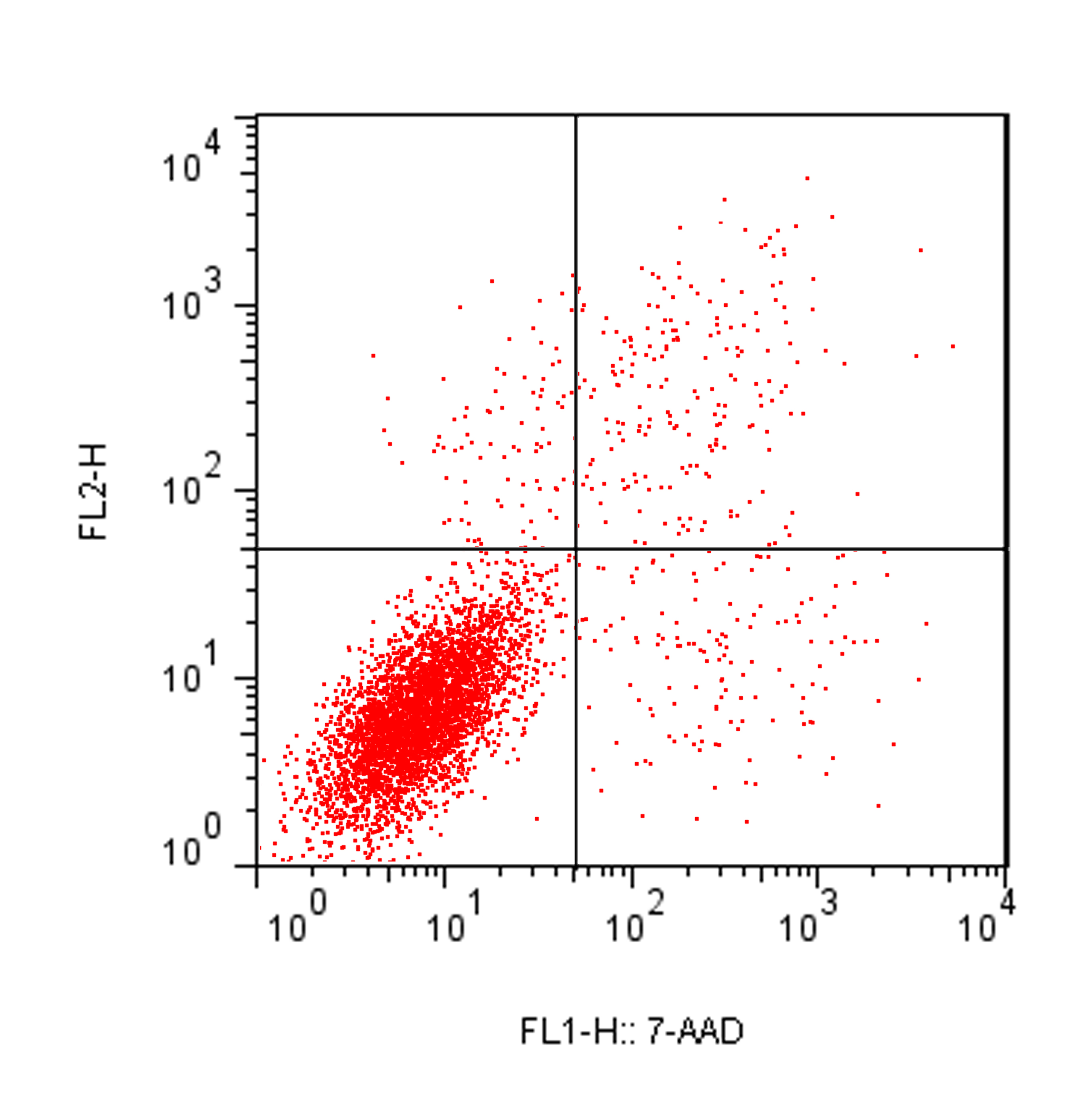

Supplement: Supplementary file 8 [file DataSheet_7.zip › Figure 7F flow cytometry/AGS/control.jpg]

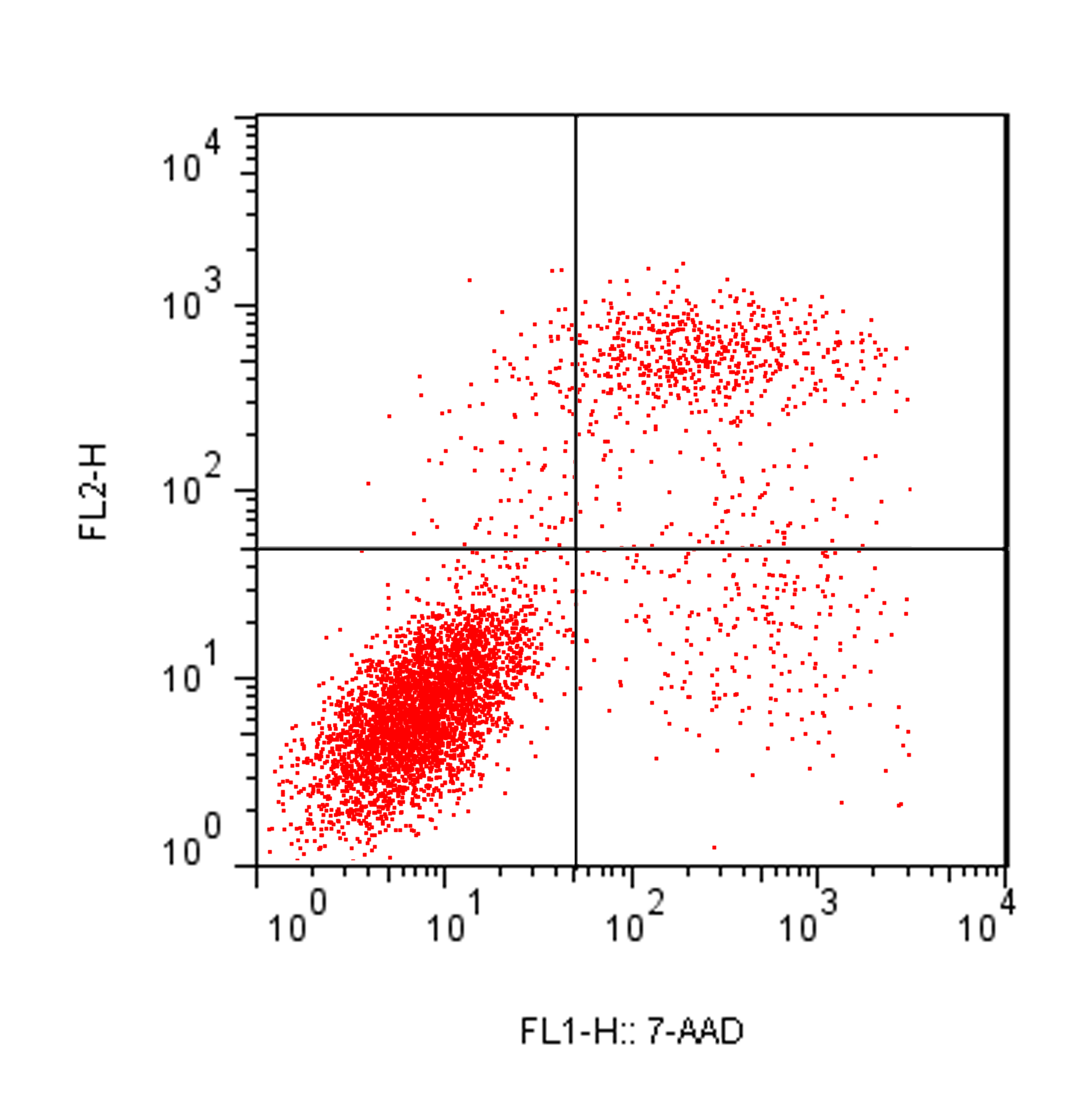

Supplement: Supplementary file 8 [file DataSheet_7.zip › Figure 7F flow cytometry/MKN45/circPTK2+AATK shRNA.jpg]

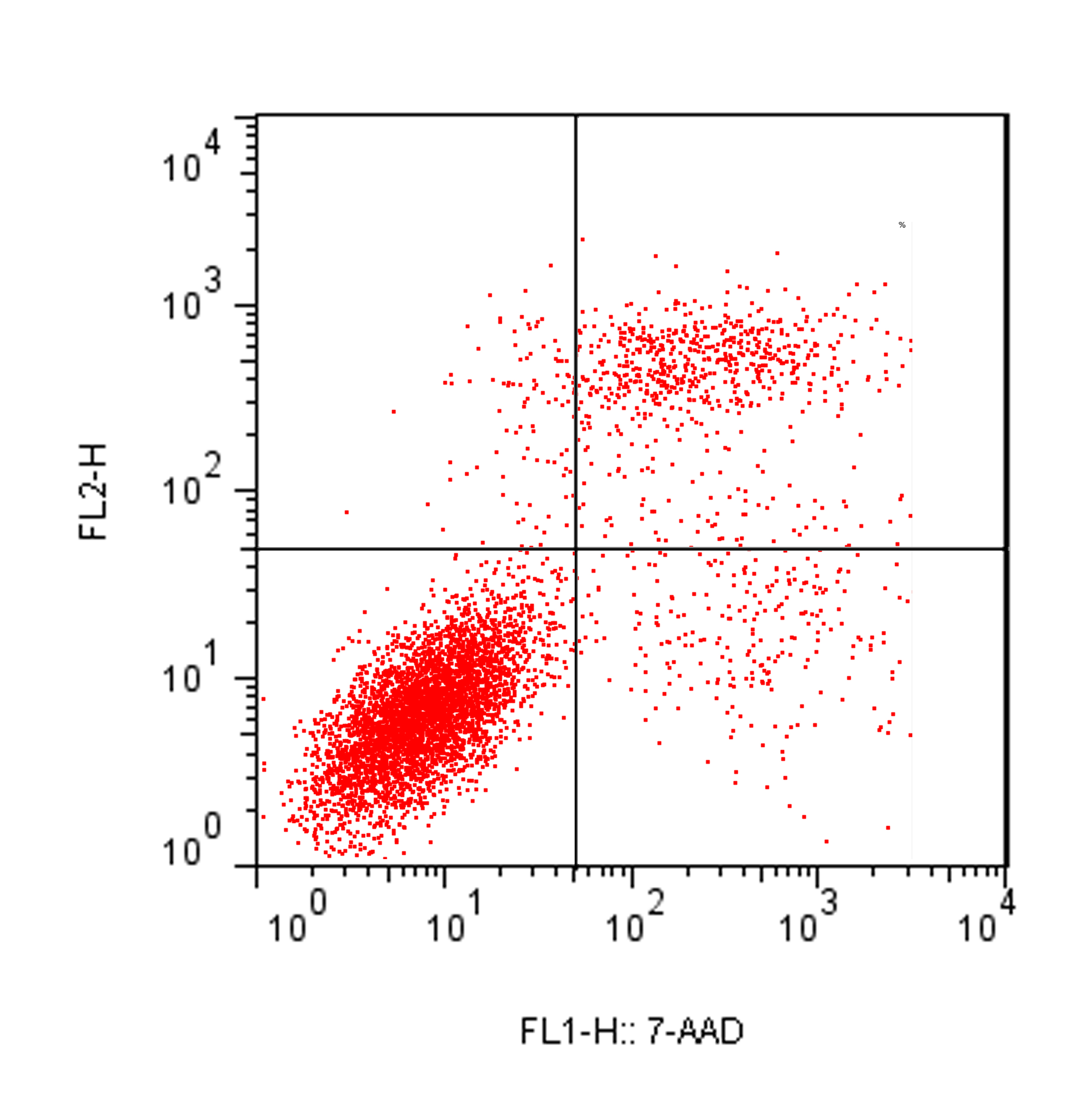

Supplement: Supplementary file 8 [file DataSheet_7.zip › Figure 7F flow cytometry/MKN45/circPTK2+miR-196a-3p.jpg]

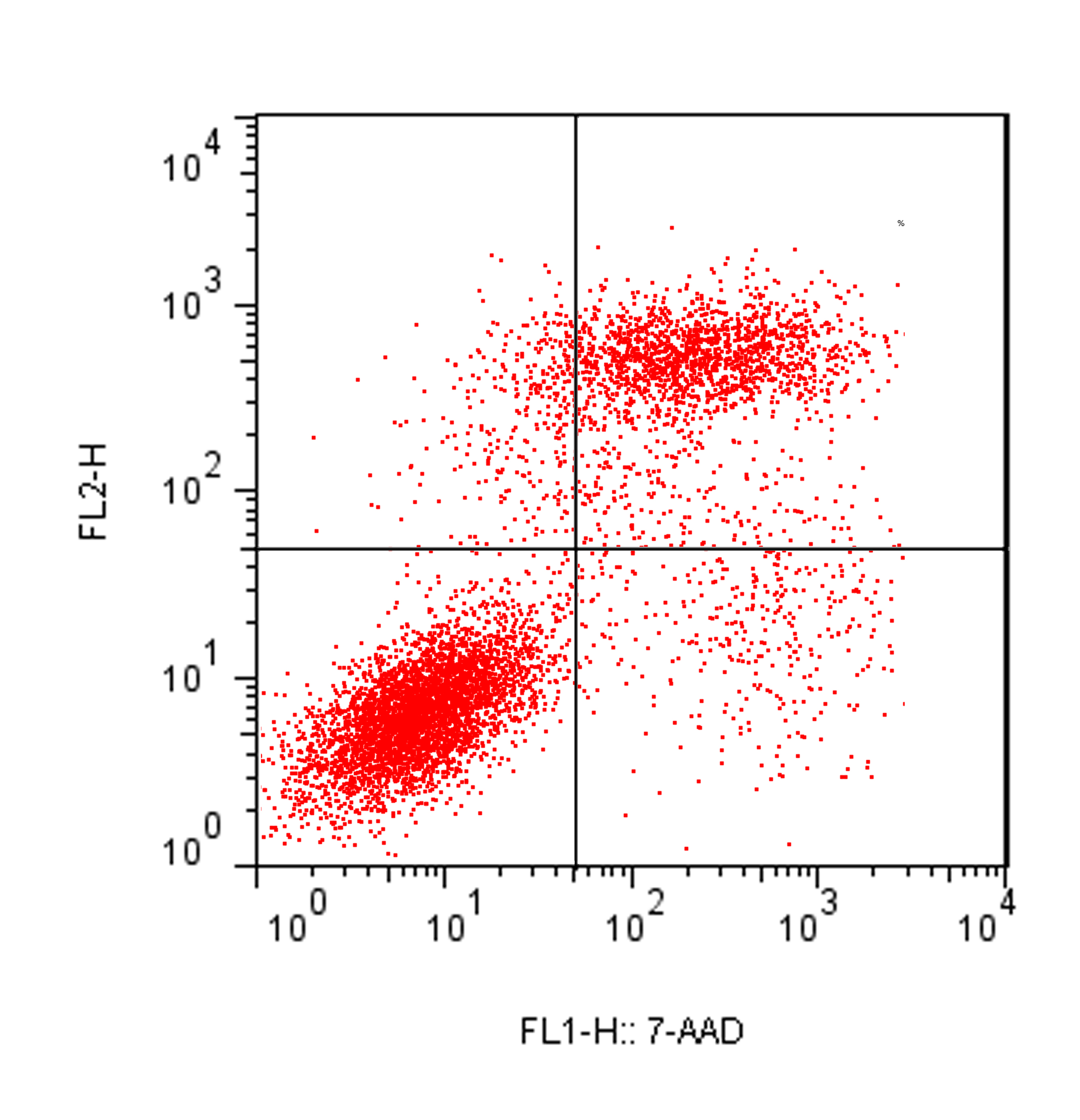

Supplement: Supplementary file 8 [file DataSheet_7.zip › Figure 7F flow cytometry/MKN45/circPTK2+mimics NC.jpg]

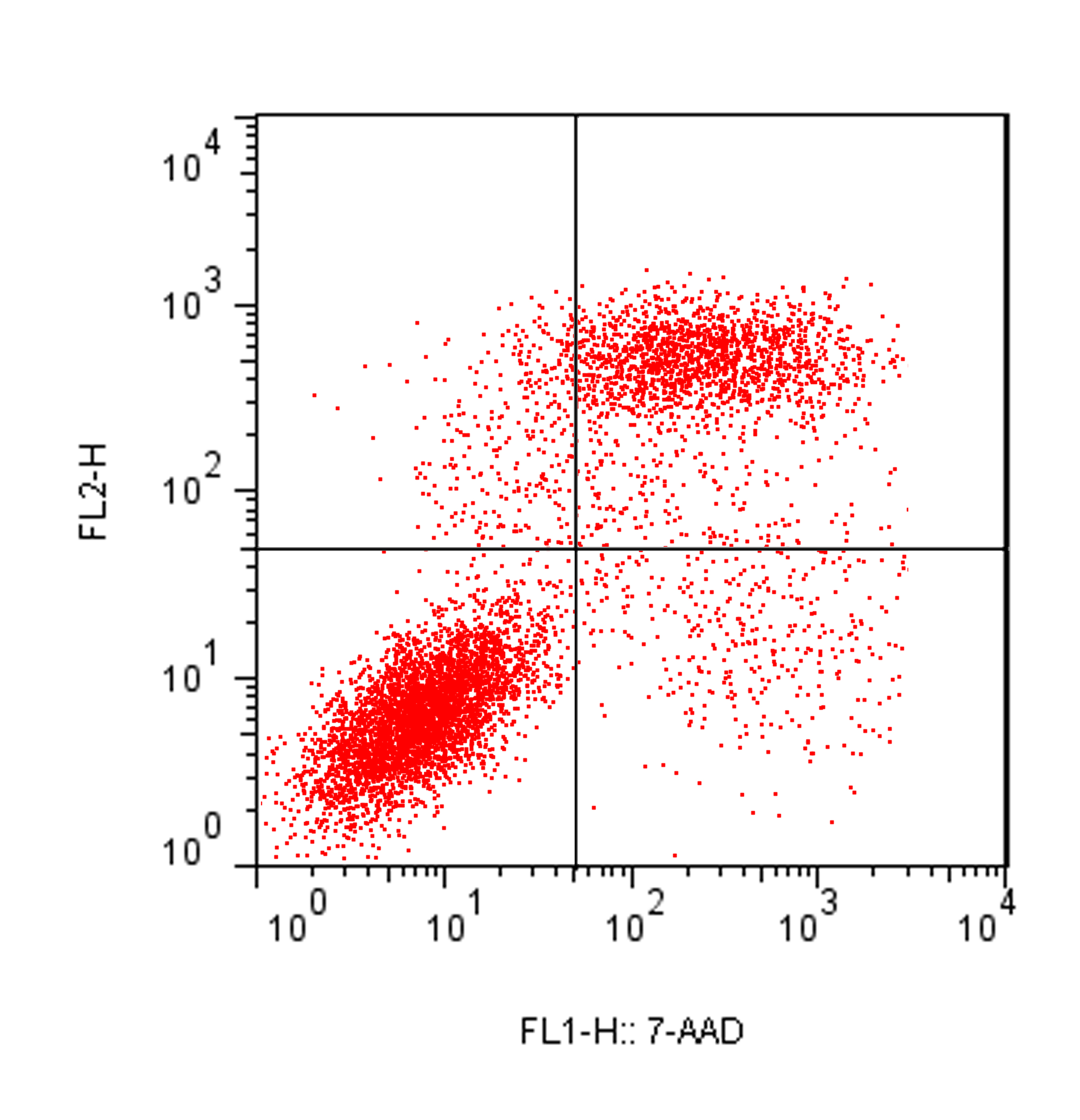

Supplement: Supplementary file 8 [file DataSheet_7.zip › Figure 7F flow cytometry/MKN45/circPTK2+shRNA NC.jpg]

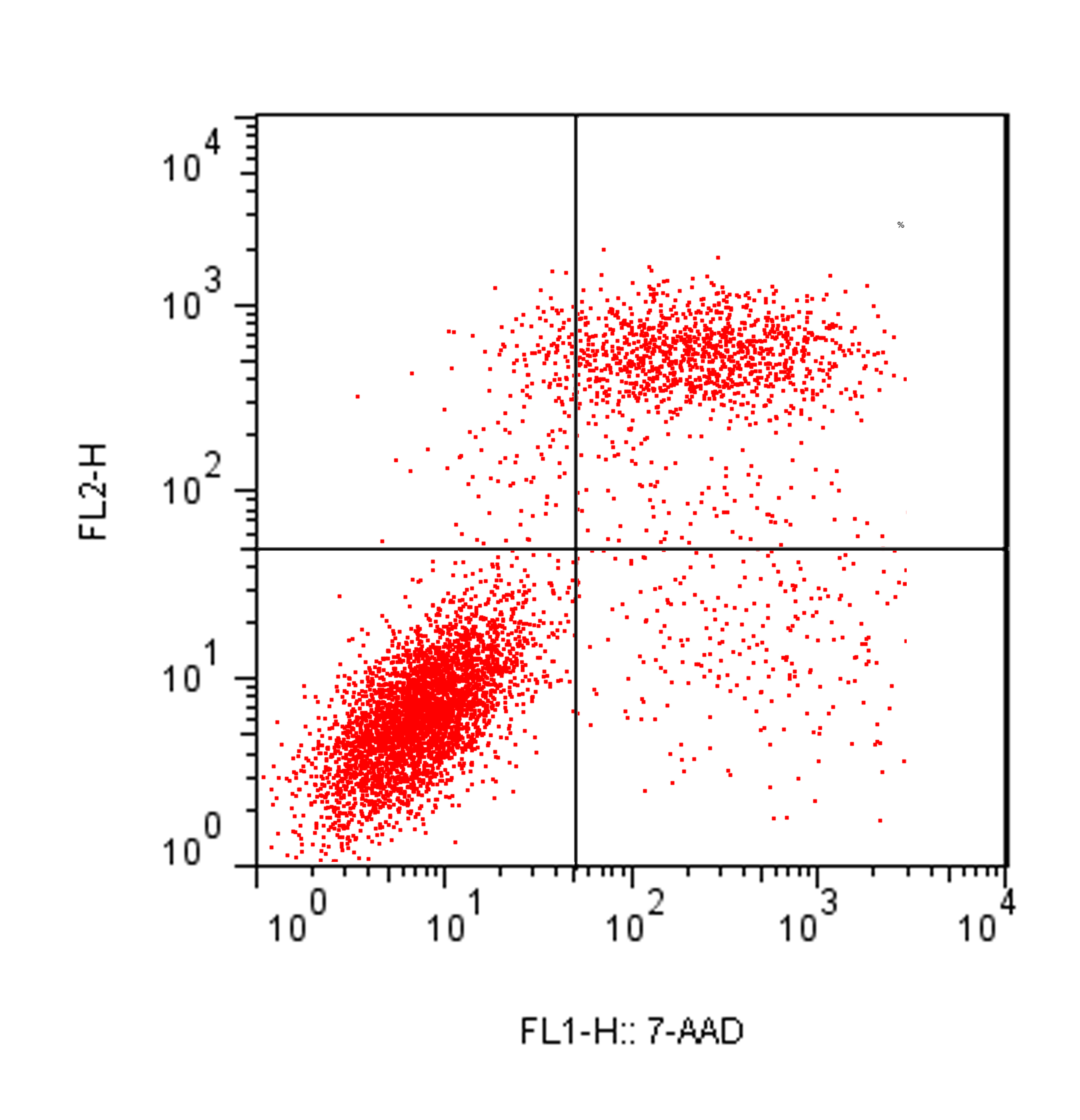

Supplement: Supplementary file 8 [file DataSheet_7.zip › Figure 7F flow cytometry/MKN45/circPTK2.jpg]

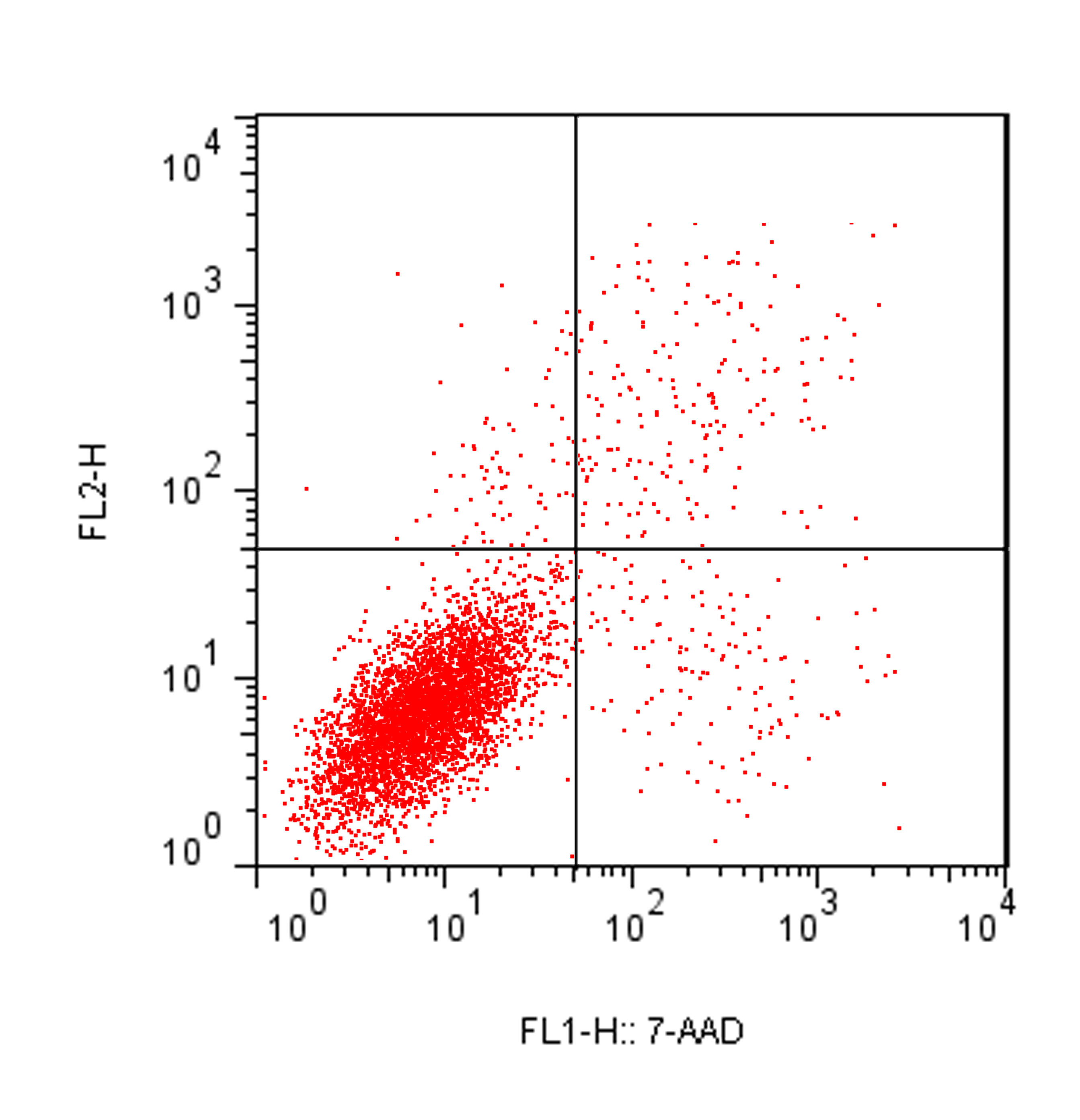

Supplement: Supplementary file 8 [file DataSheet_7.zip › Figure 7F flow cytometry/MKN45/control.jpg]

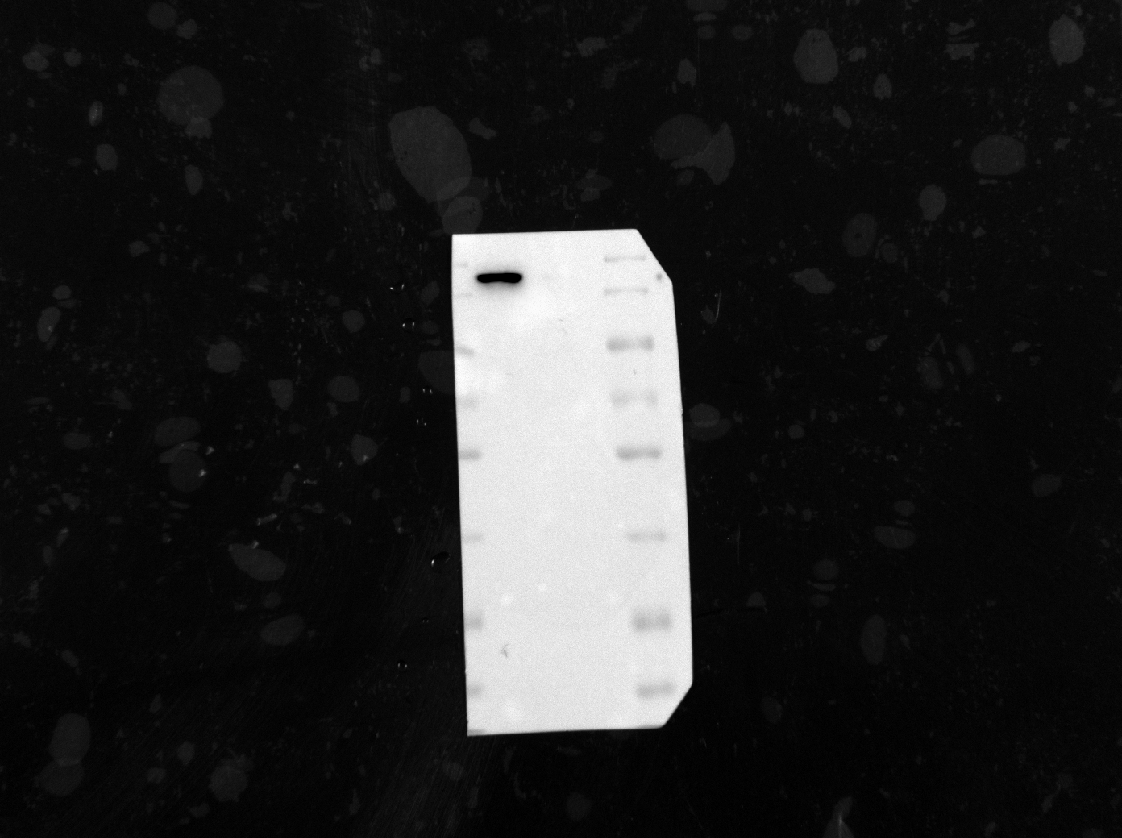

Supplement: Supplementary file 9 [file DataSheet_8.zip › Figure 8B/IP AATK-left.tif]

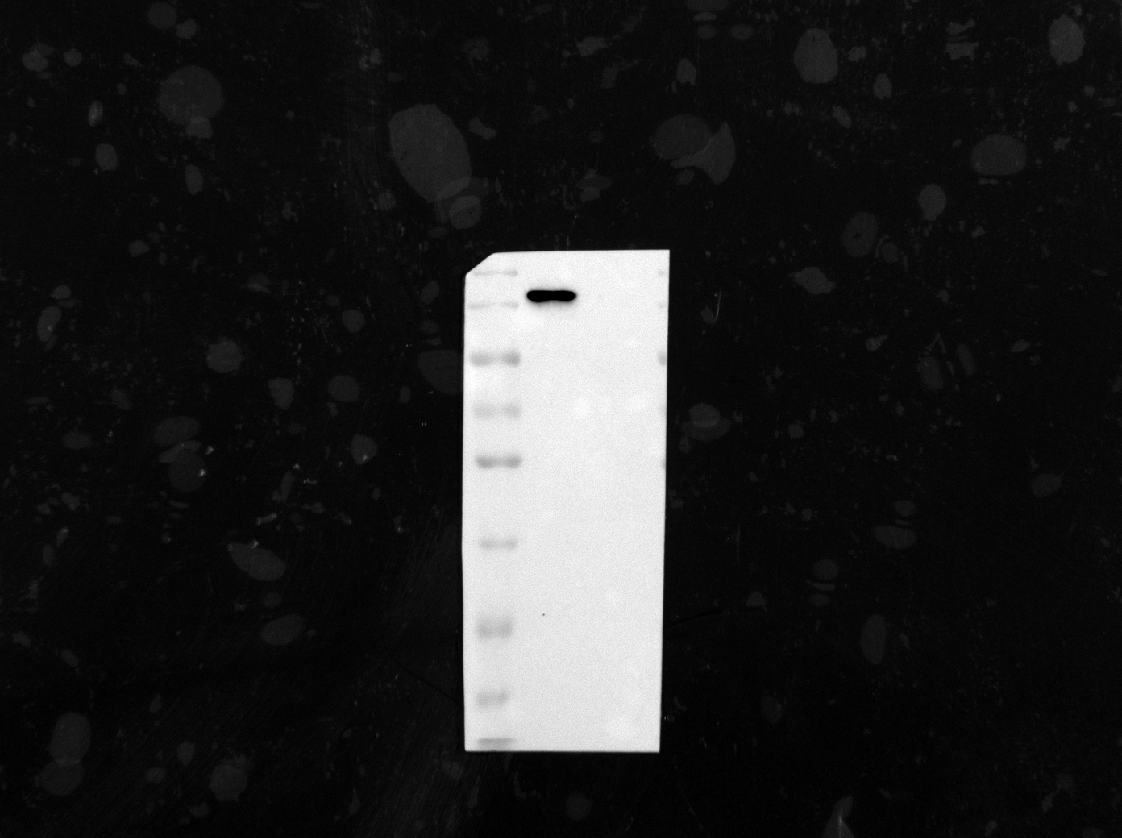

Supplement: Supplementary file 9 [file DataSheet_8.zip › Figure 8B/IP AATK-right.tif]

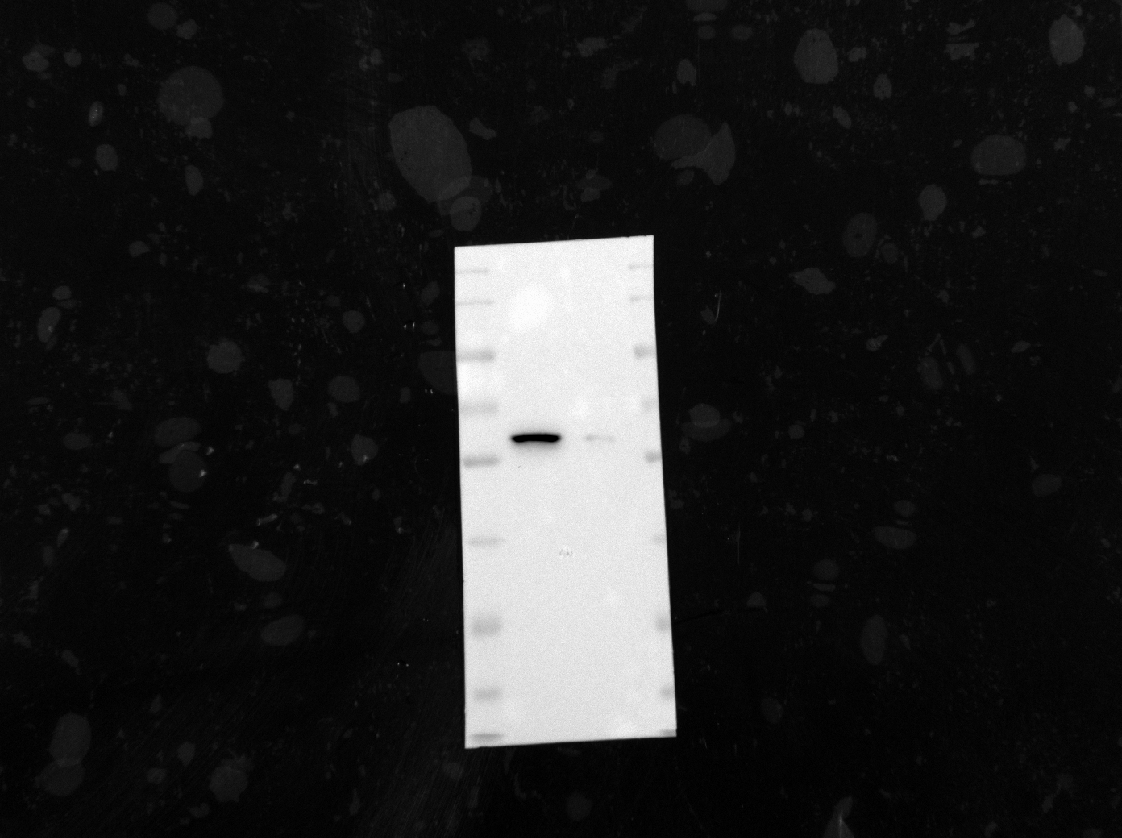

Supplement: Supplementary file 9 [file DataSheet_8.zip › Figure 8B/IP STK39-left.tif]

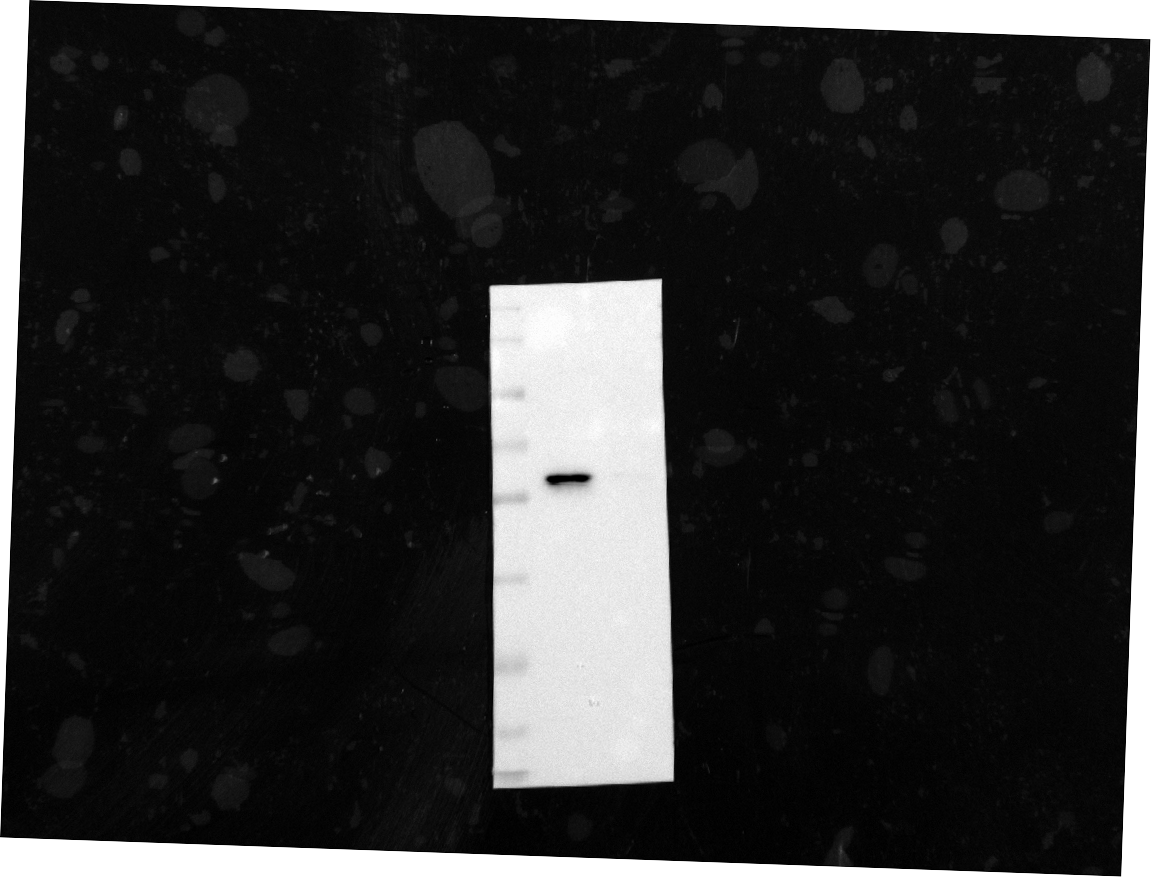

Supplement: Supplementary file 9 [file DataSheet_8.zip › Figure 8B/IP STK39-right.tif]

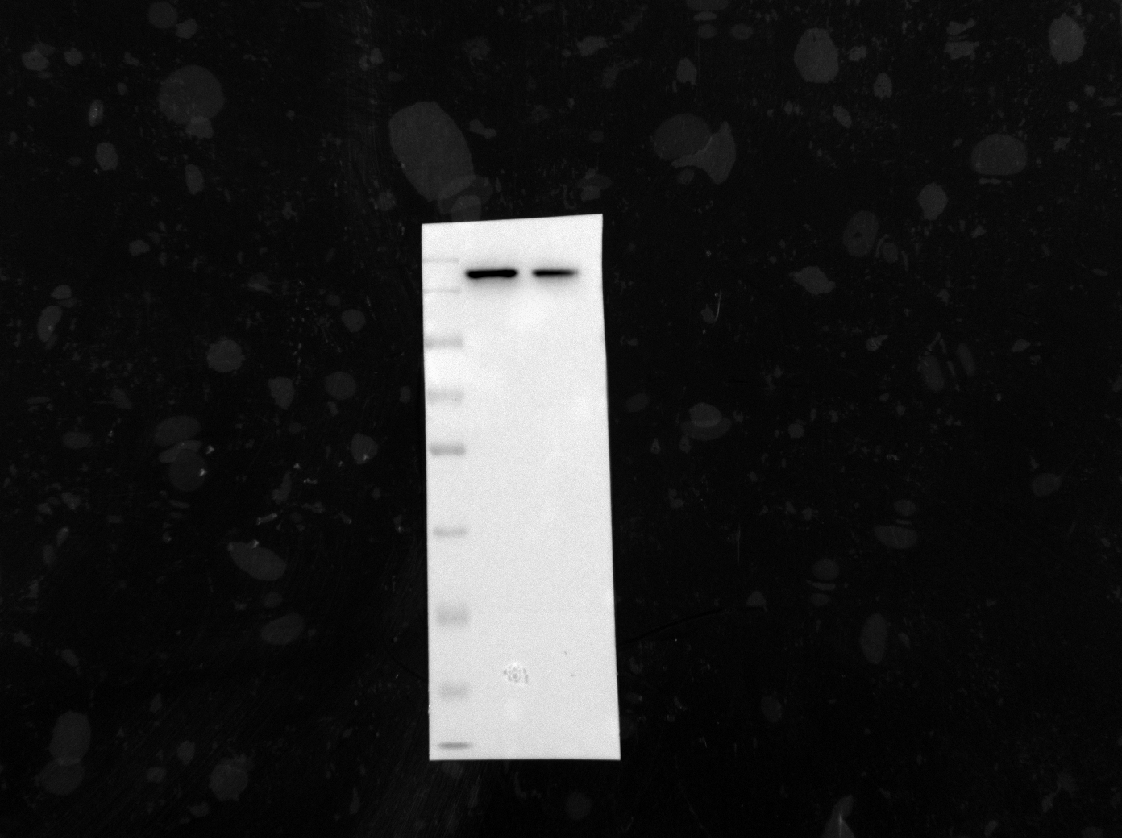

Supplement: Supplementary file 9 [file DataSheet_8.zip › Figure 8B/Input AATK-left.tif]

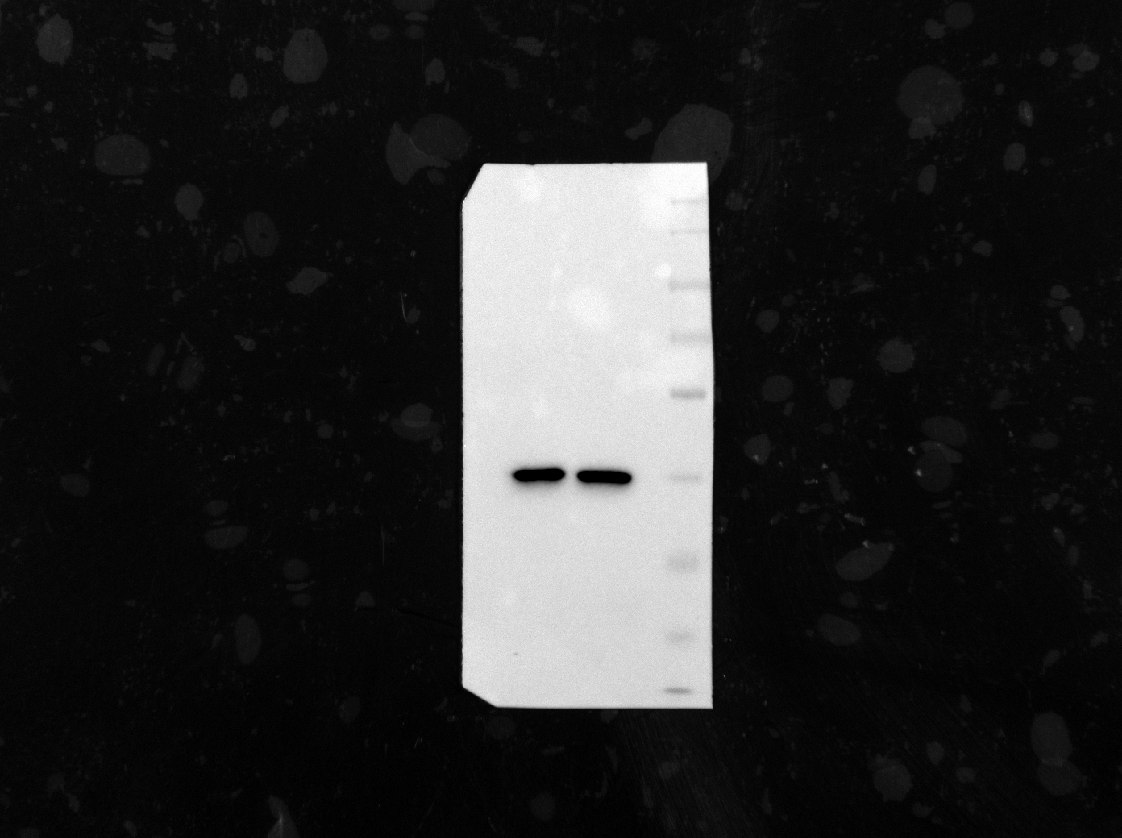

Supplement: Supplementary file 9 [file DataSheet_8.zip › Figure 8B/Input GAPDH-left.tif]

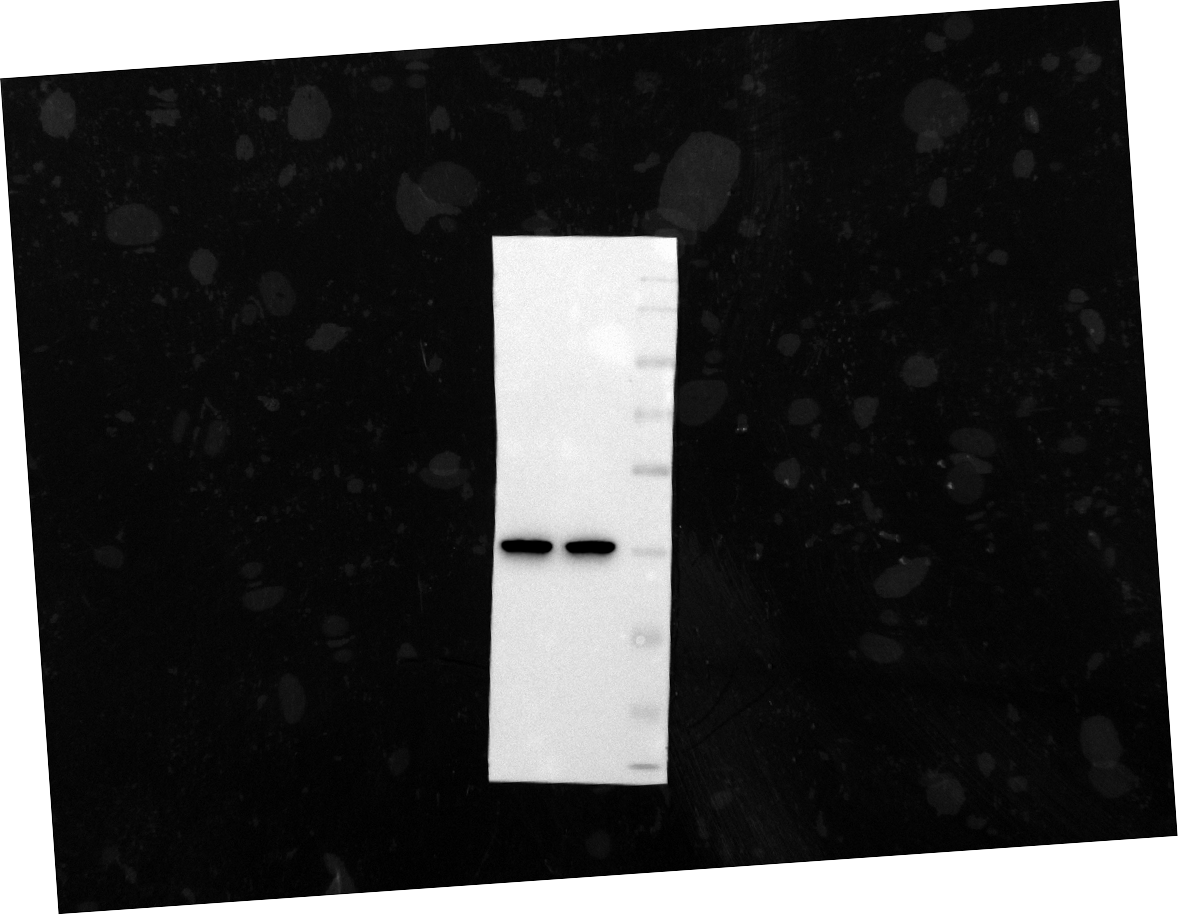

Supplement: Supplementary file 9 [file DataSheet_8.zip › Figure 8B/Input GAPDH-right.tif]

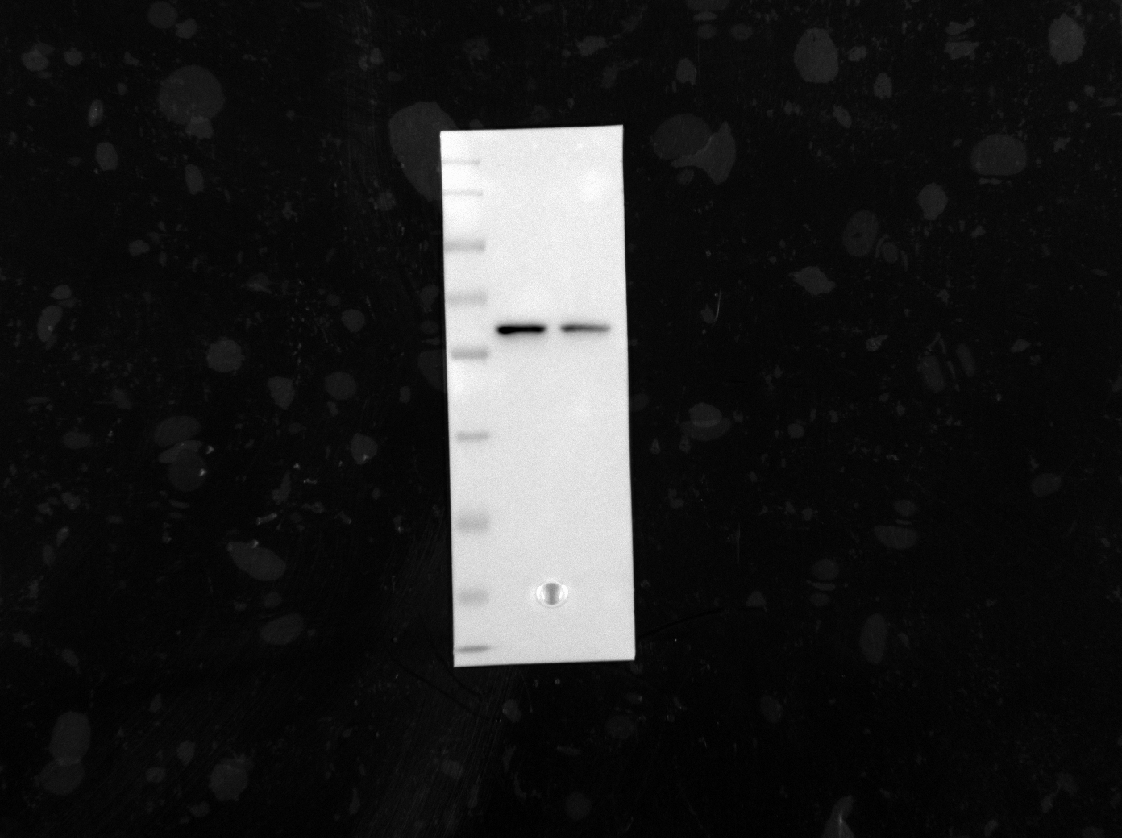

Supplement: Supplementary file 9 [file DataSheet_8.zip › Figure 8B/Input STK39-right.tif]

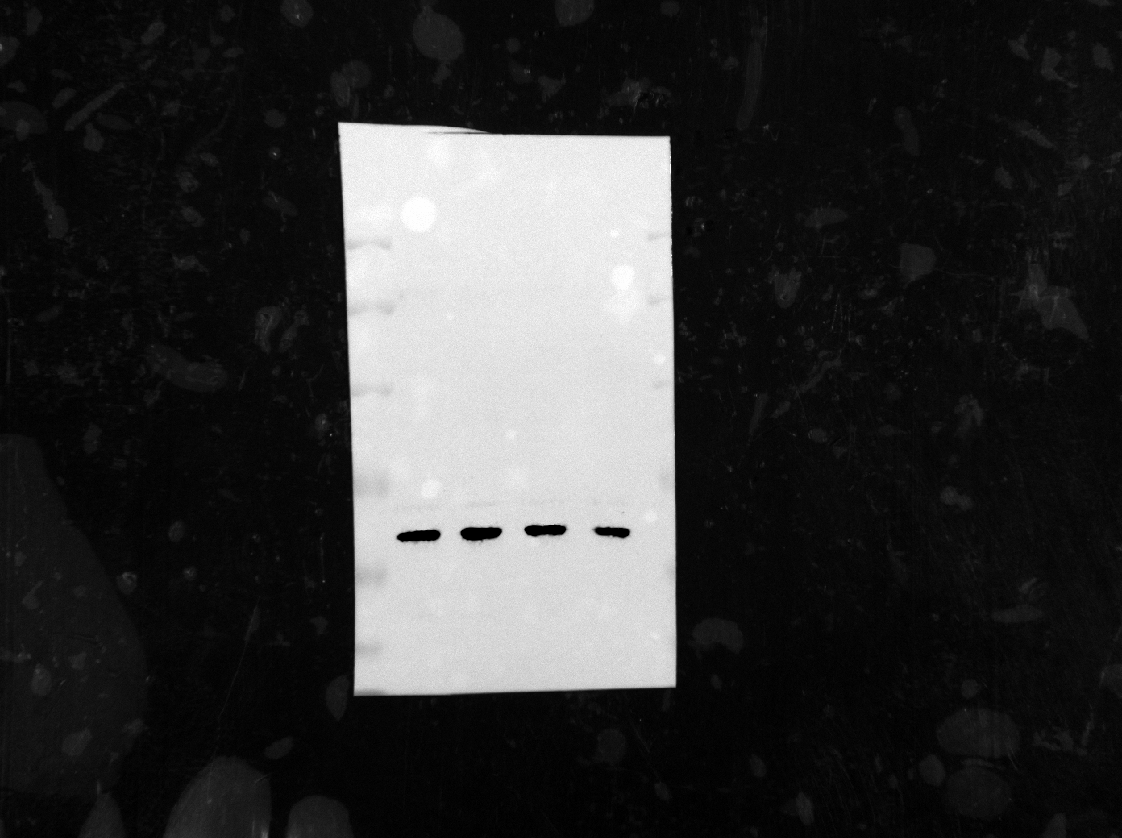

Supplement: Supplementary file 10 [file DataSheet_9.zip › Figure 8C/STK39-left.tif]

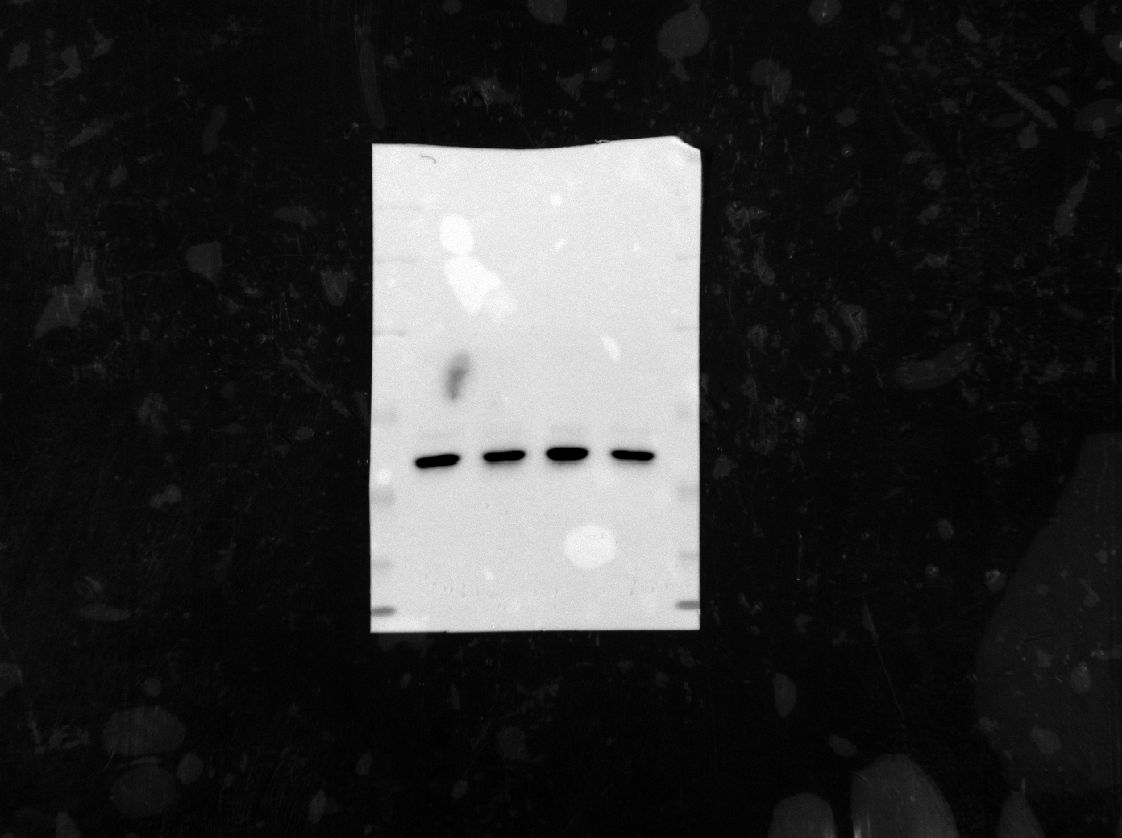

Supplement: Supplementary file 10 [file DataSheet_9.zip › Figure 8C/STK39-right.tif]

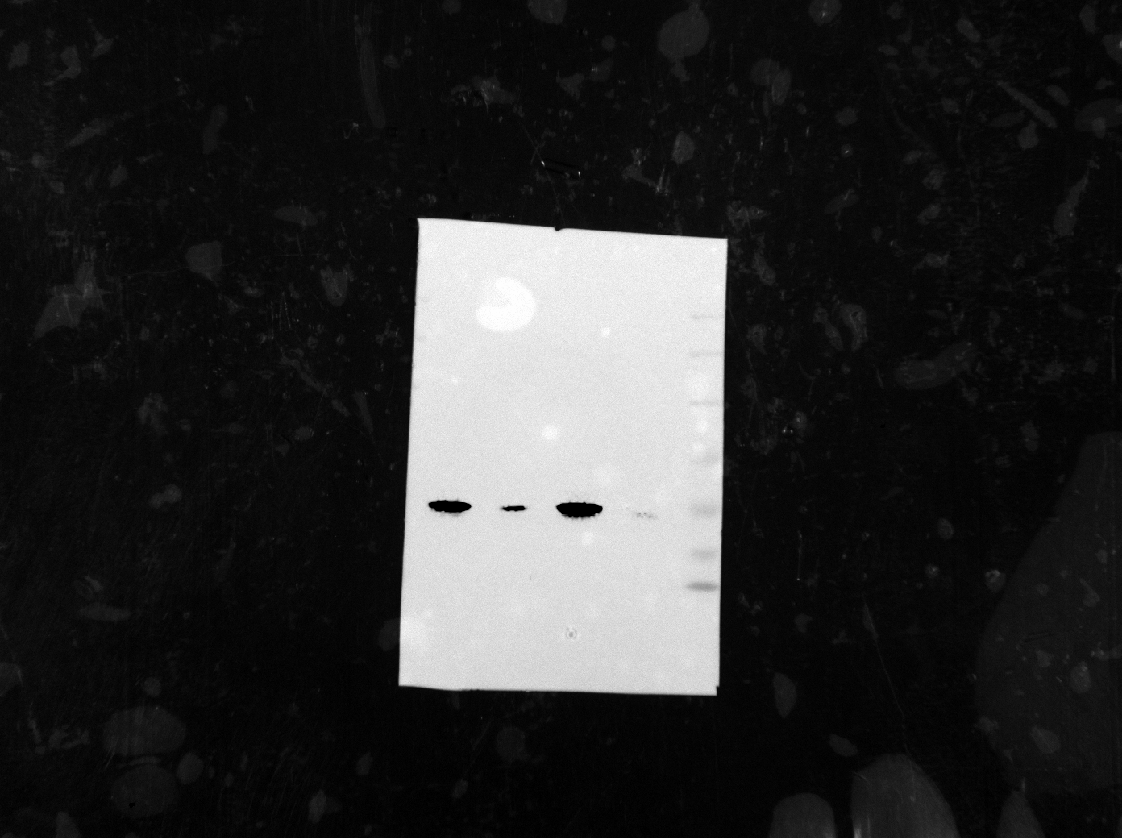

Supplement: Supplementary file 10 [file DataSheet_9.zip › Figure 8C/p-STK39-left.tif]

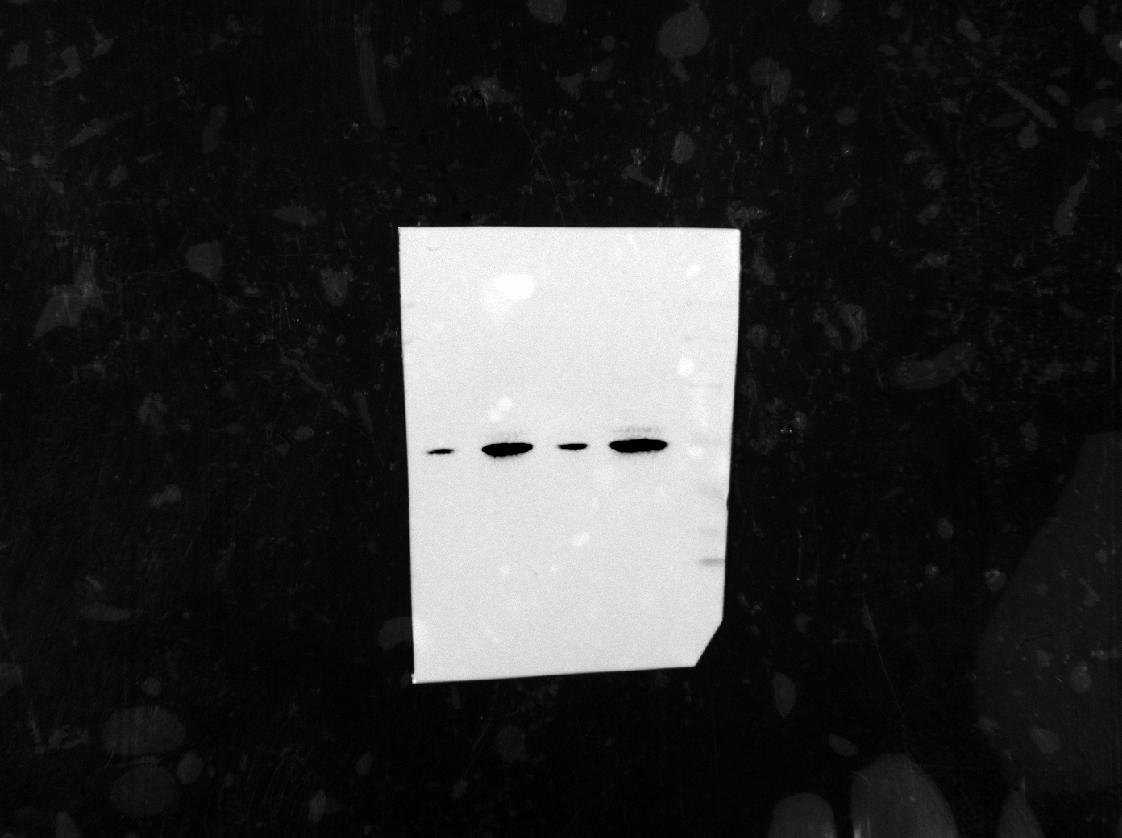

Supplement: Supplementary file 10 [file DataSheet_9.zip › Figure 8C/p-STK39-right.tif]

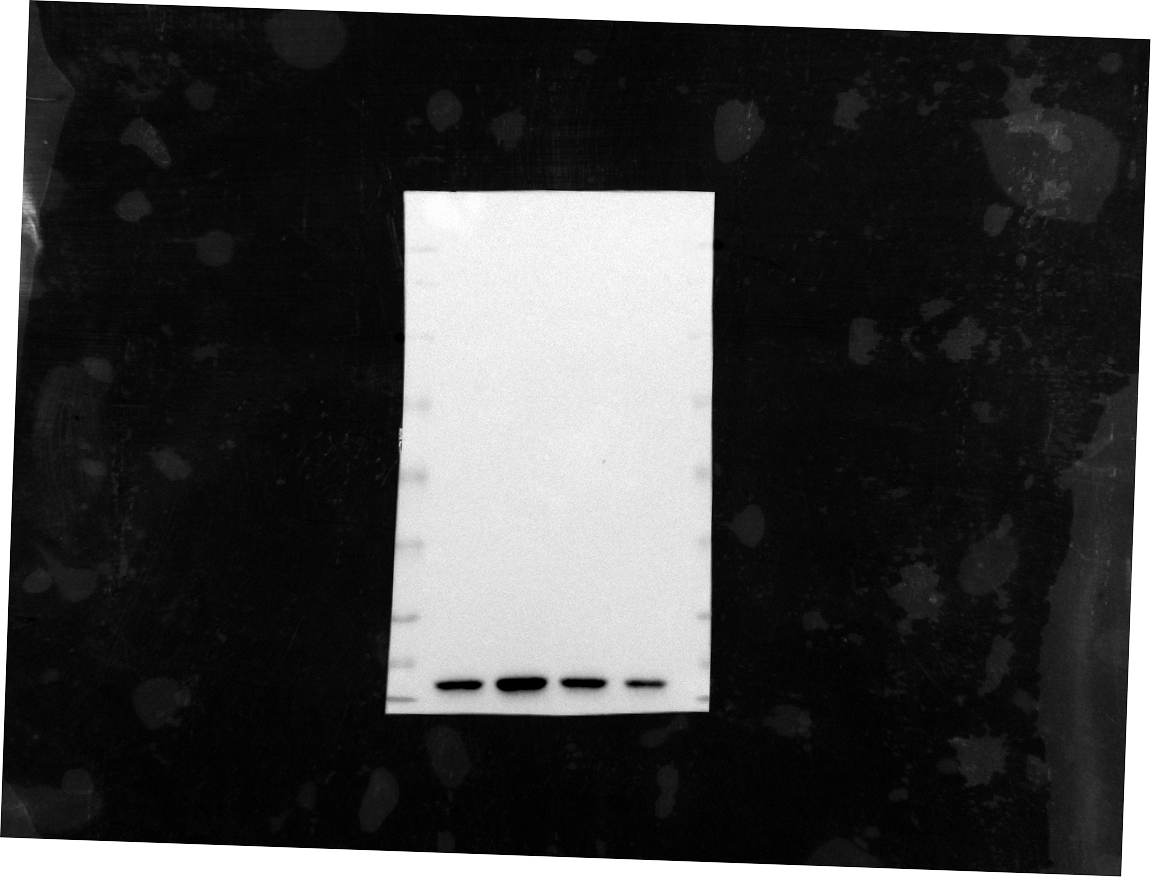

Supplement: Supplementary file 11 [file DataSheet_10.zip › Figure 8D/Bax-AGS.tif]

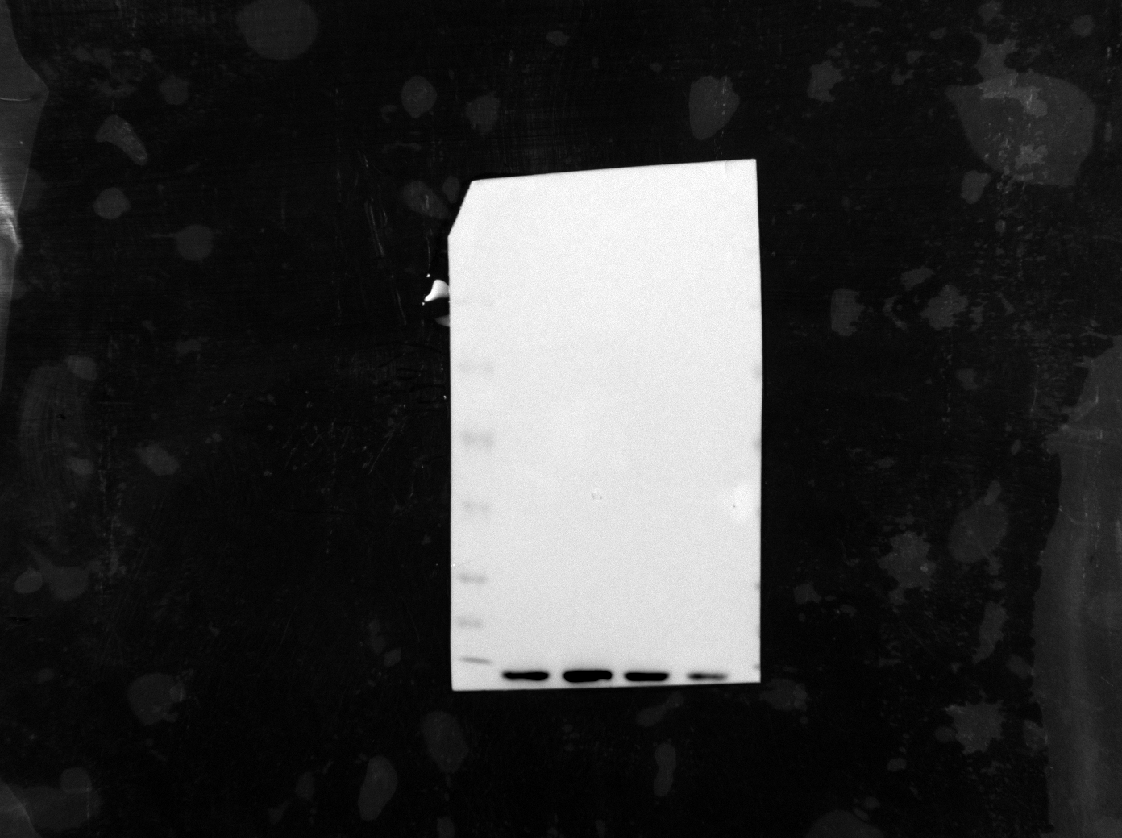

Supplement: Supplementary file 11 [file DataSheet_10.zip › Figure 8D/Bax-MKN45.tif]

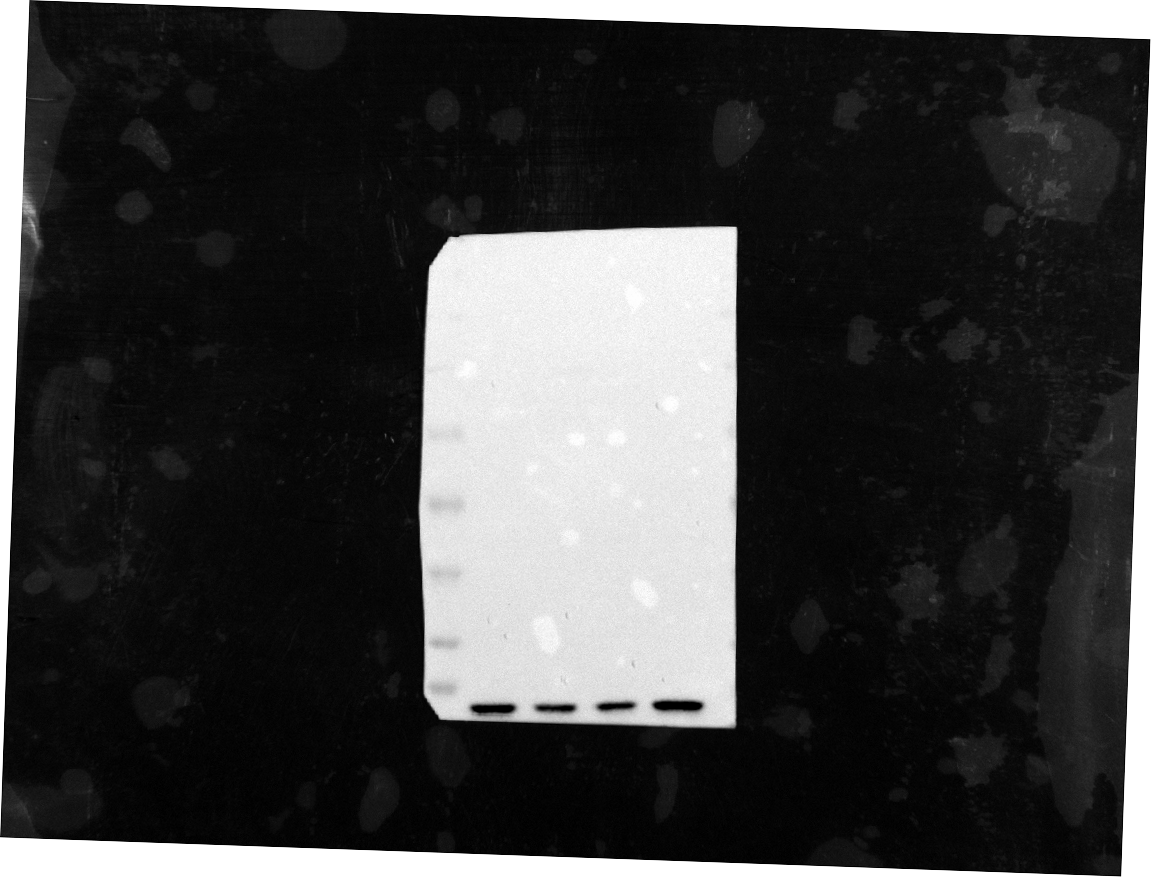

Supplement: Supplementary file 11 [file DataSheet_10.zip › Figure 8D/Bcl-2-AGS.tif]
