# Supplementary figures and images for: CircPTK2 Suppresses the Progression of Gastric Cancer by Targeting the MiR-196a-3p/AATK Axis (part 2 of 2)
Source: Front Oncol. 2021 Sep 15;11:706415. doi: 10.3389/fonc.2021.706415 (PMC8479173; doi:10.3389/fonc.2021.706415)

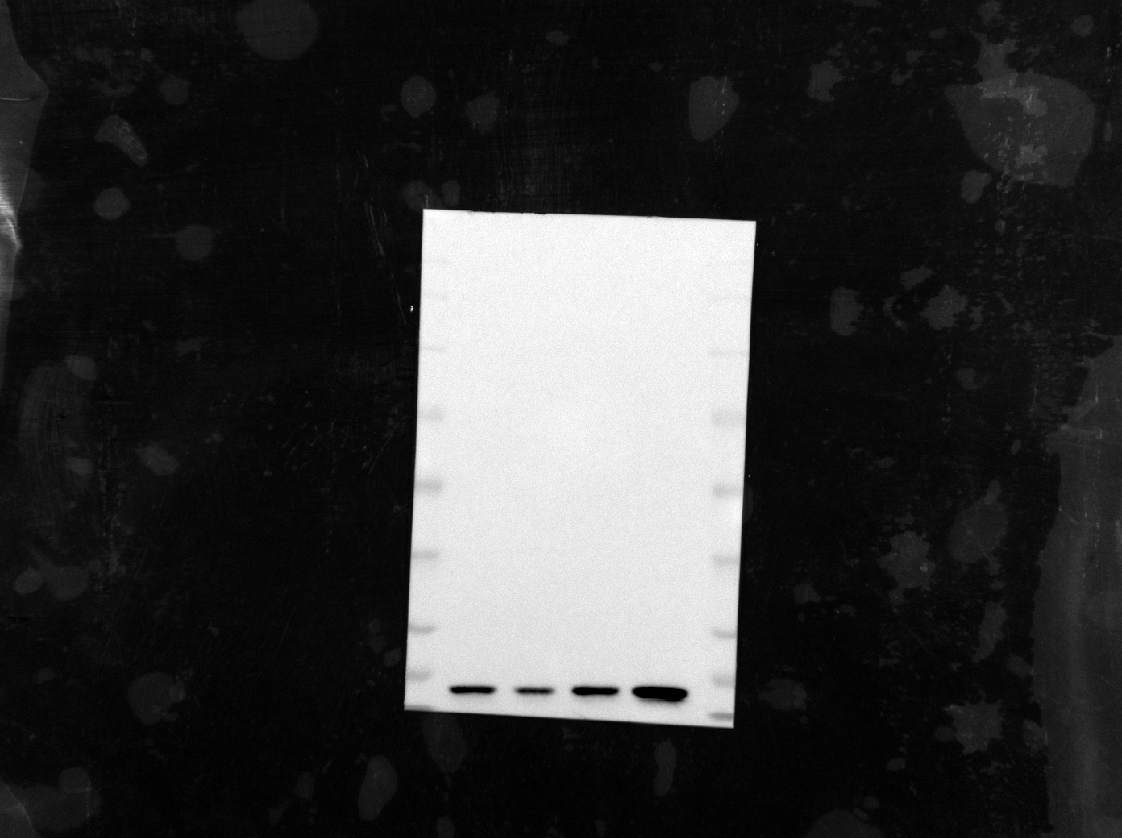

Supplement: Supplementary file 11 [file DataSheet_10.zip › Figure 8D/Bcl-2-MKN45.tif]

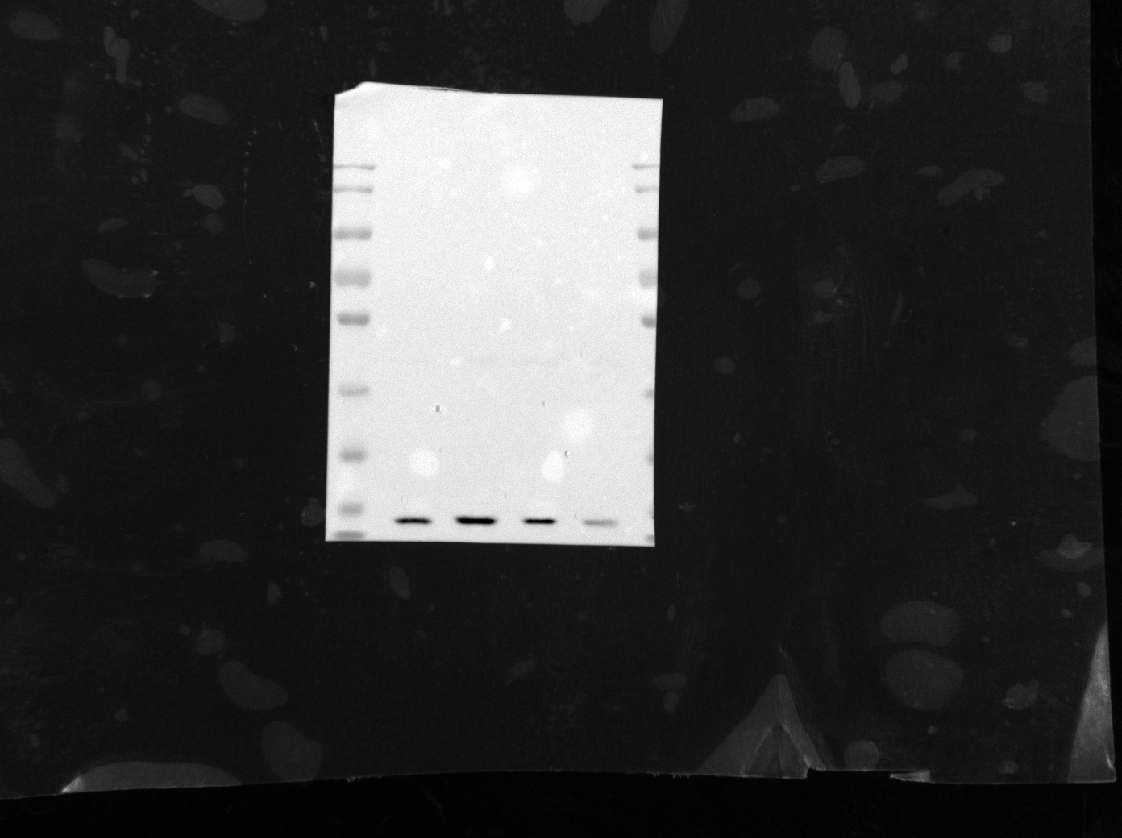

Supplement: Supplementary file 11 [file DataSheet_10.zip › Figure 8D/Cleaved caspase3-MKN45.tif]

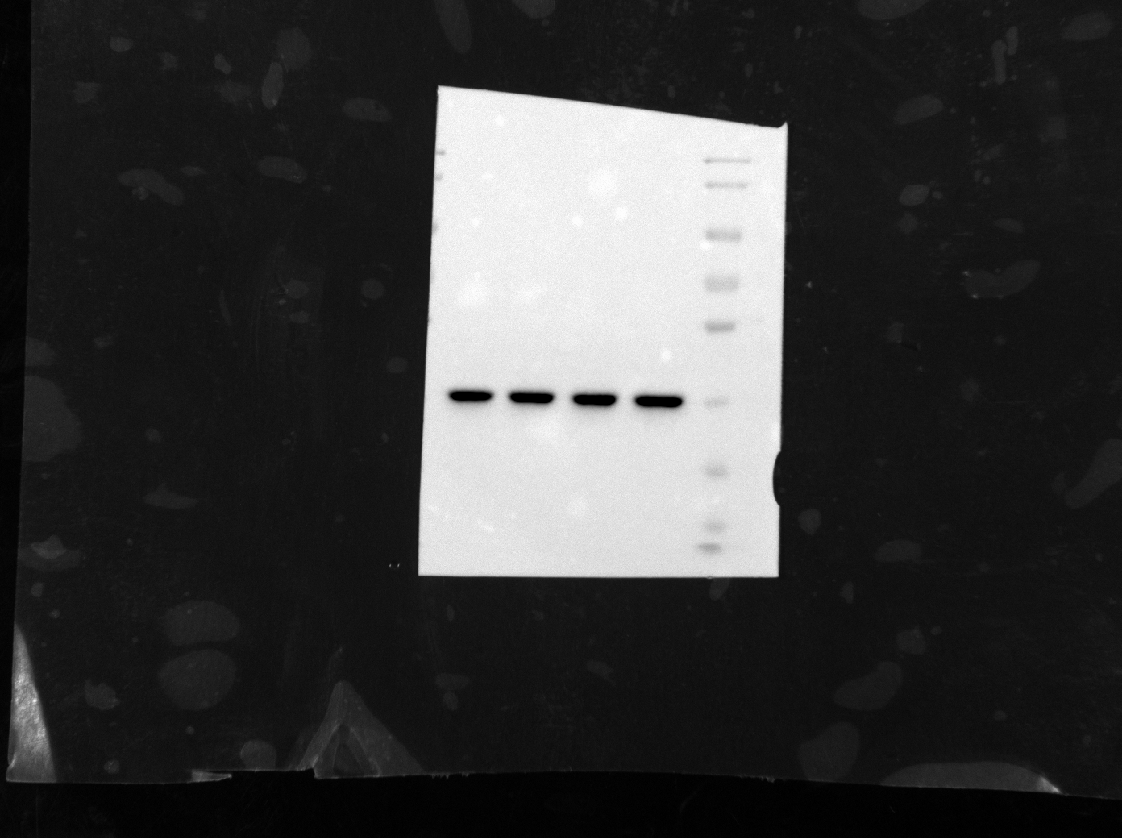

Supplement: Supplementary file 11 [file DataSheet_10.zip › Figure 8D/GAPDH-AGS.tif]

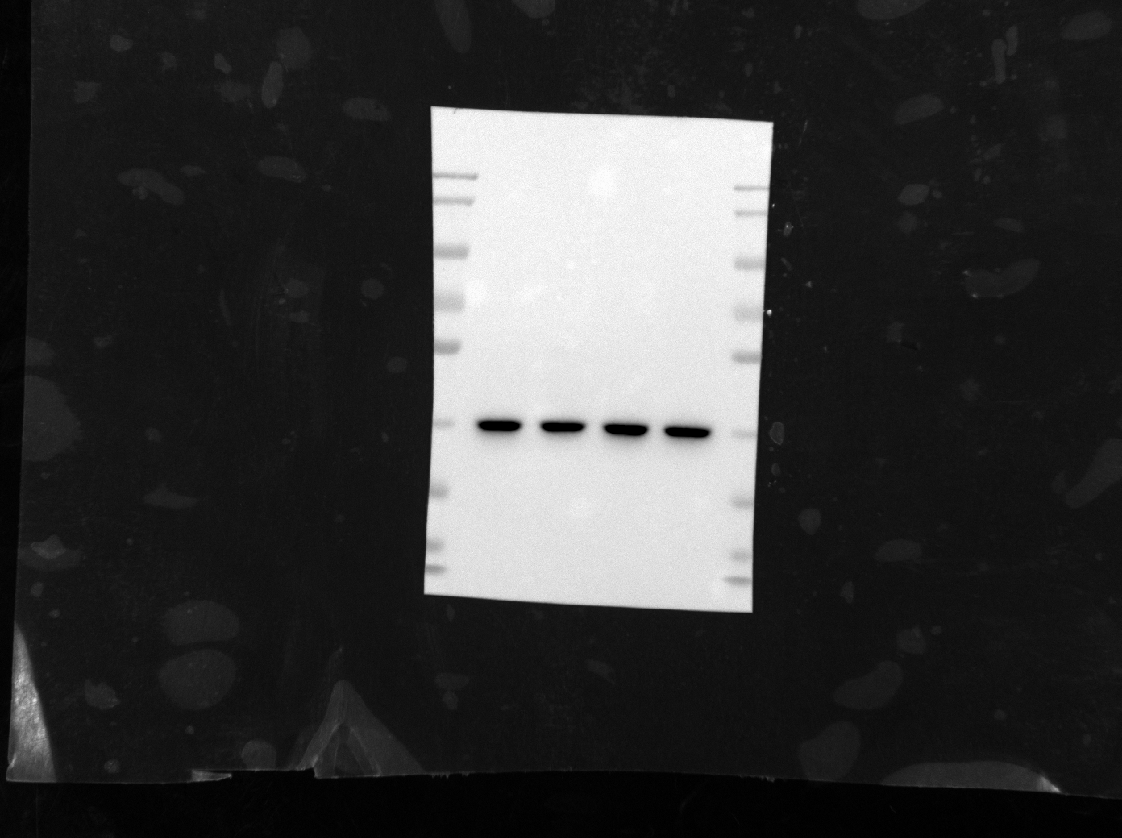

Supplement: Supplementary file 11 [file DataSheet_10.zip › Figure 8D/GAPDH-MKN45.tif]

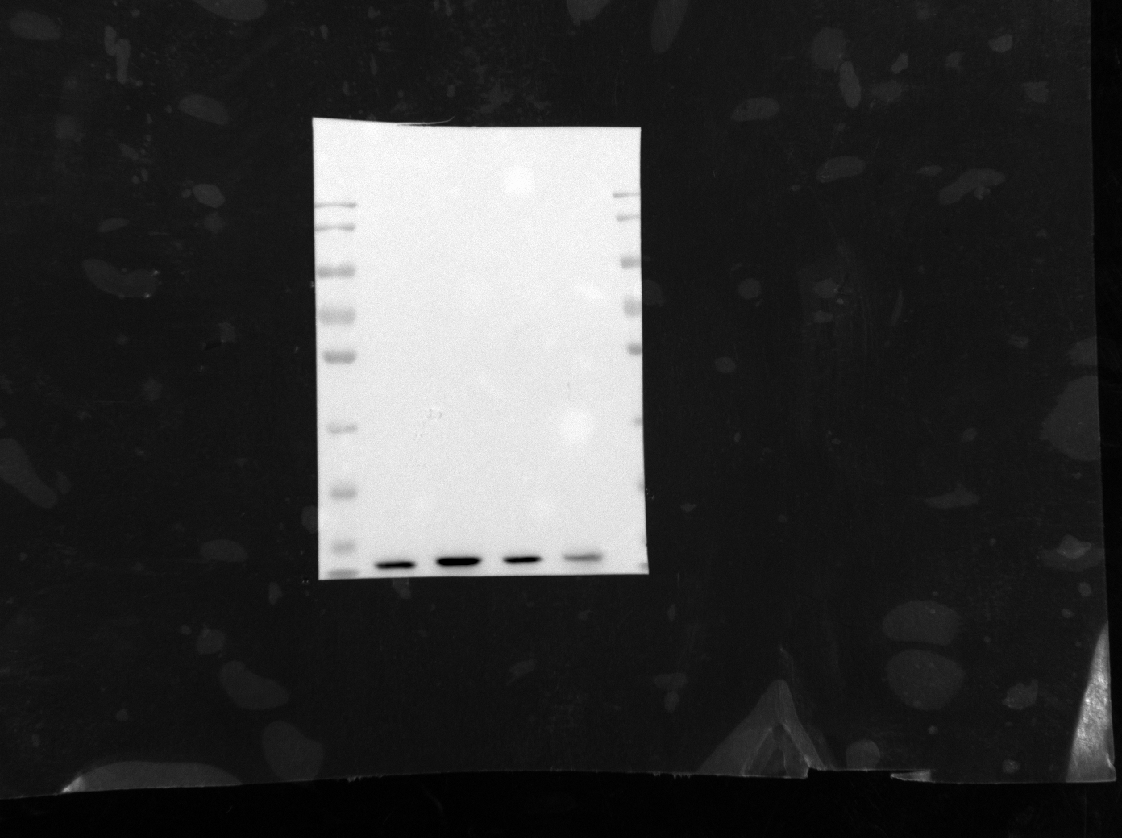

Supplement: Supplementary file 11 [file DataSheet_10.zip › Figure 8D/cleaved caspase3-AGS.tif]

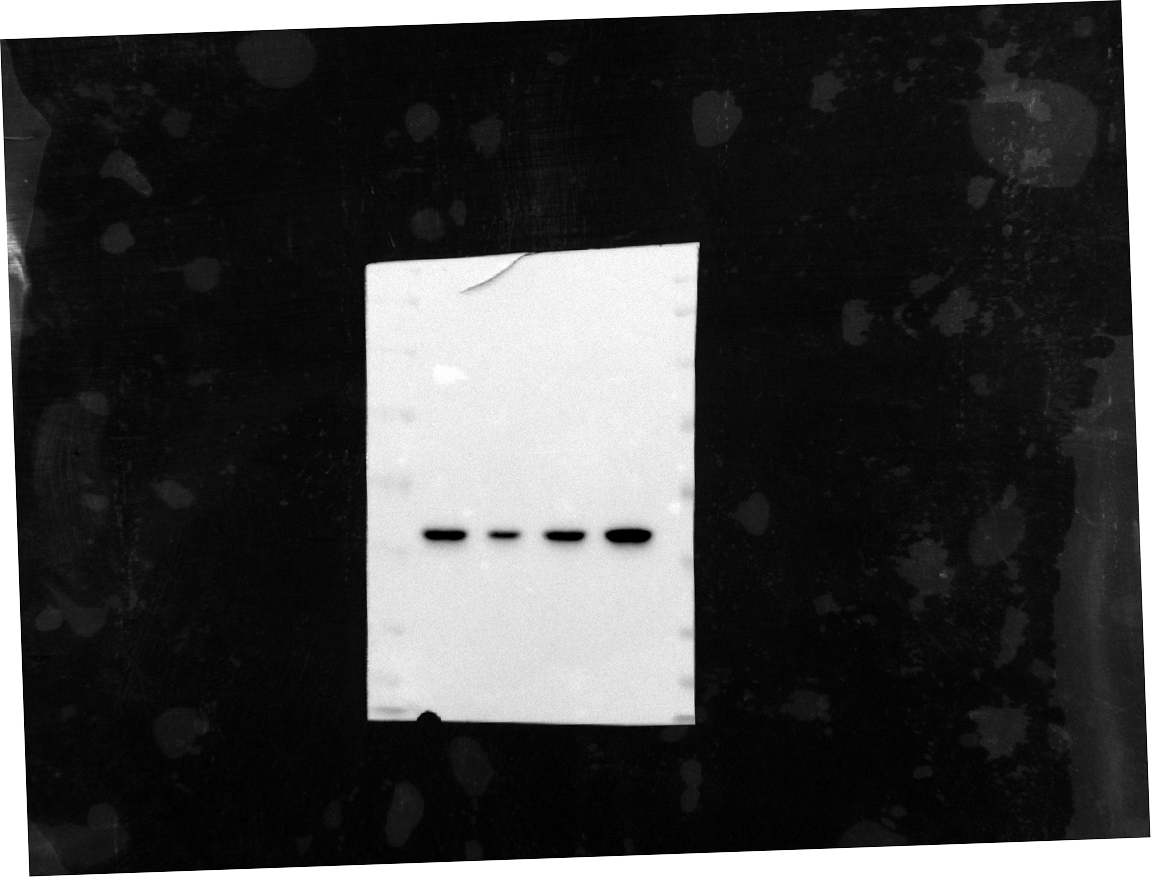

Supplement: Supplementary file 11 [file DataSheet_10.zip › Figure 8D/p-p38-AGS.tif]

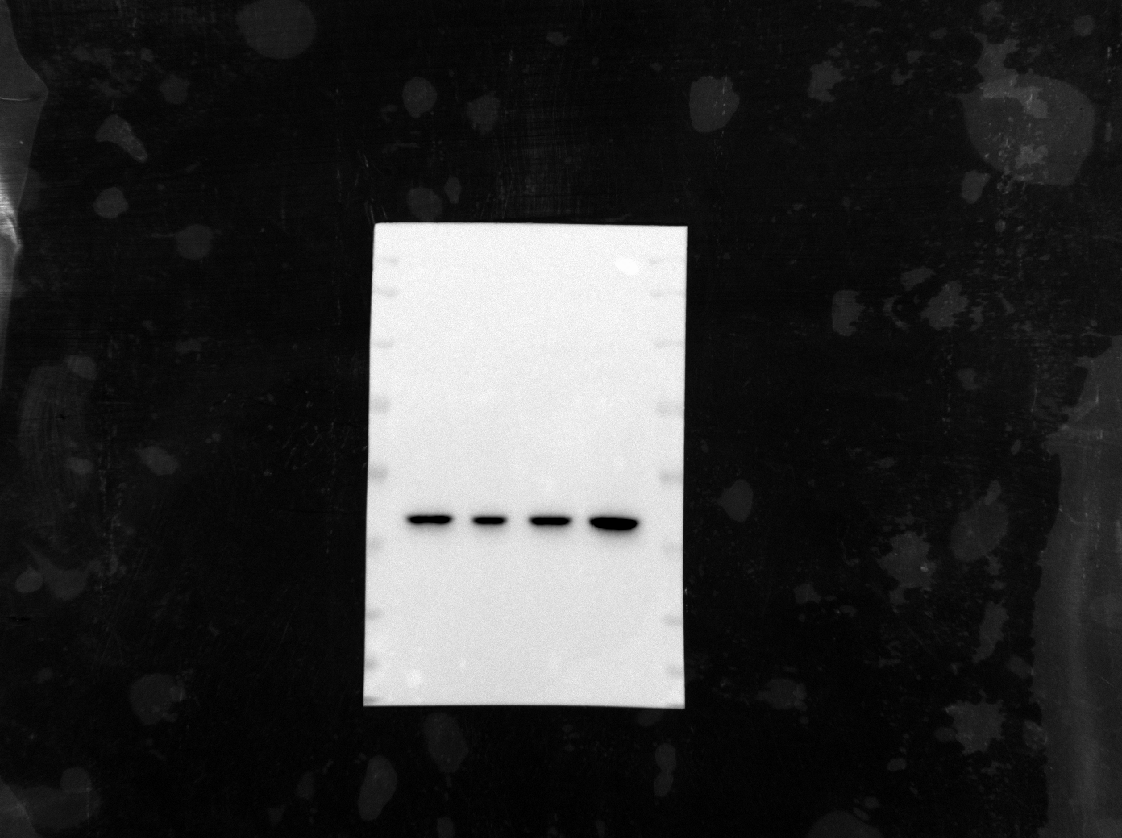

Supplement: Supplementary file 11 [file DataSheet_10.zip › Figure 8D/p-p38-MKN45.tif]

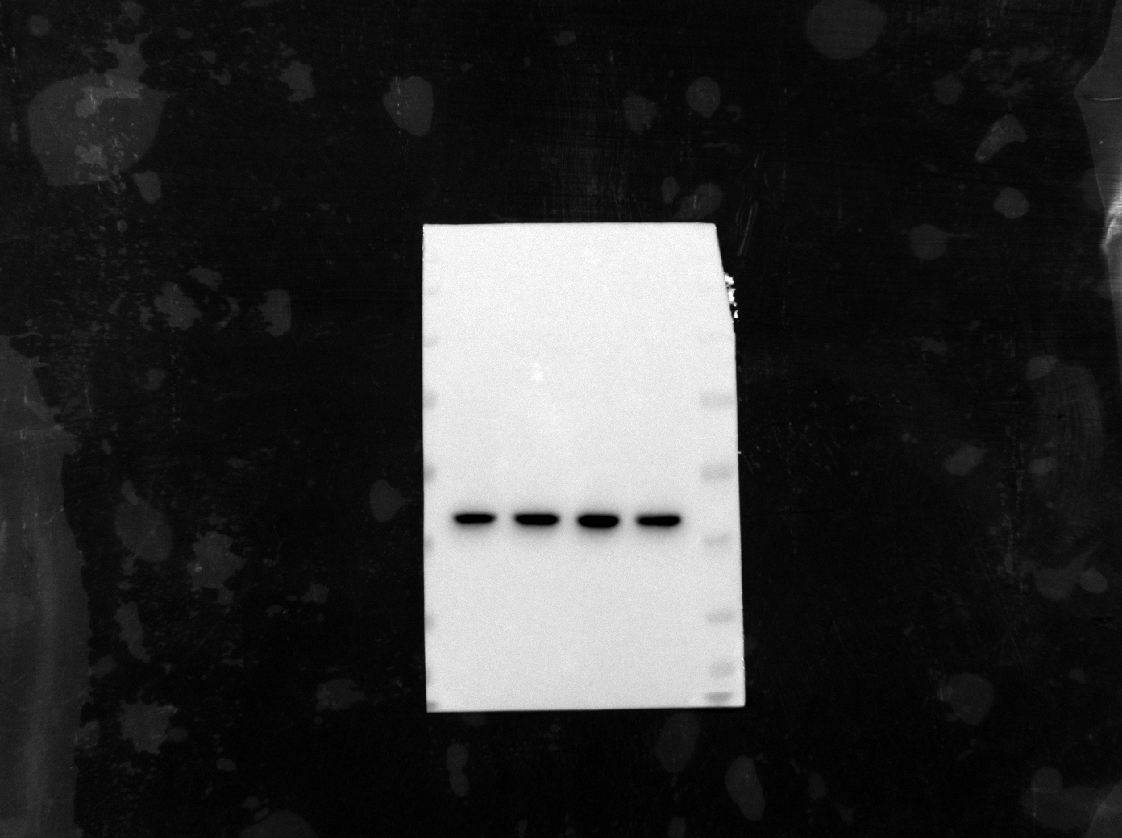

Supplement: Supplementary file 11 [file DataSheet_10.zip › Figure 8D/p38-AGS.tif]

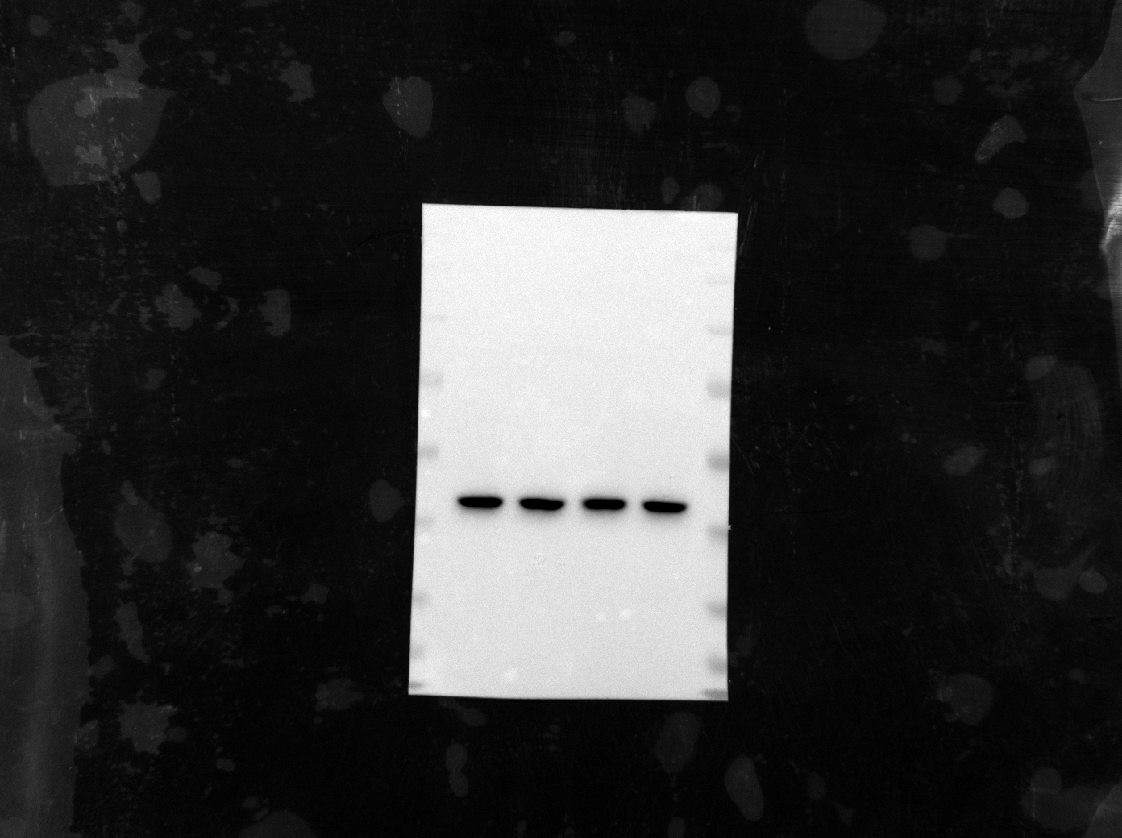

Supplement: Supplementary file 11 [file DataSheet_10.zip › Figure 8D/p38-MKN45.tif]
